# Unsupervised multiscale clustering of single-cell transcriptomes to identify hierarchical structures of cell subtypes

**DOI:** 10.1093/gigascience/giaf111

**Published:** 2025-10-09

**Authors:** Won-Min Song, Chen Ming, Christian V Forst, Bin Zhang

**Affiliations:** Department of Genetics and Genomic Sciences, Icahn School of Medicine at Mount Sinai, New York, NY 10029, USA; Mount Sinai Center for Transformative Disease Modeling, Icahn School of Medicine at Mount Sinai, New York, NY 10029, USA; Department of Genetics and Genomic Sciences, Icahn School of Medicine at Mount Sinai, New York, NY 10029, USA; Mount Sinai Center for Transformative Disease Modeling, Icahn School of Medicine at Mount Sinai, New York, NY 10029, USA; Faculty of Health Sciences, University of Macau, Avenida da Universidade, Taipa, Macau, China; Department of Genetics and Genomic Sciences, Icahn School of Medicine at Mount Sinai, New York, NY 10029, USA; Mount Sinai Center for Transformative Disease Modeling, Icahn School of Medicine at Mount Sinai, New York, NY 10029, USA; Department of Microbiology, Icahn School of Medicine at Mount Sinai, New York, NY 10029, USA; Department of Genetics and Genomic Sciences, Icahn School of Medicine at Mount Sinai, New York, NY 10029, USA; Mount Sinai Center for Transformative Disease Modeling, Icahn School of Medicine at Mount Sinai, New York, NY 10029, USA

**Keywords:** multiscale clustering, scRNA-seq, bioinformatics, similarity network

## Abstract

**Background:**

Cell clustering is an essential step in uncovering cellular architectures in single-cell RNA sequencing (scRNA-seq) data. However, the existing cell clustering approaches are not well designed to dissect complex structures of cellular landscapes at a finer resolution.

**Results:**

Here, we develop a multiscale clustering (MSC) approach to construct a sparse cell–cell correlation network for unsupervised identification of *de novo* cell types and subtypes across multiple resolutions. Based upon simulated silver- and gold-standard data as well as real scRNA-seq data in diseases, MSC demonstrates significantly improved performance compared to established benchmark methods and reveals a biologically meaningful cell hierarchy to facilitate the discovery of novel disease-associated cell subtypes and mechanisms.

**Conclusions:**

We present MSC as a new single-cell multiscale clustering framework as a powerful tool for advancing discoveries in disease-associated cell populations using single-cell sequencing data.

## Background

Single-cell sequencing enables the extraction of molecular features at the cellular resolution to elucidate heterogeneous cellular landscapes in various tissues under different conditions (e.g., development and disease). Cellular heterogeneity often manifests as distinct subtypes within certain cell types, and some of these are associated with certain conditions under a study. For example, previous studies have identified expanded inflammatory monocytes in patients with COVID-19[1], a microglia subtype associated with Alzheimer’s disease [2, 3], and exclusion of cytotoxic T cells in tumors [4]. Unsupervised cell clustering analysis is crucial to capturing these heterogeneous cellular landscapes in various conditions, especially to identify novel cell populations [5, 6].

Graph-theoretic approaches have been popular for understanding clustering structures in single-cell RNA sequencing (scRNA-seq) to identify meaningful subpopulation architectures. These graph-theoretic approaches often utilize the k-nearest neighbor (kNN) network and its variant, shared nearest neighbor (SNN) networks, to construct the cell similarity networks [7–9], followed by the search for closely connected subnetworks by Reichardt–Bornholdt (RB) modularity (*Q_RB_*) optimization. *Q_RB_* is a variant of Newman’s modularity (*Q_N_*) modularity to quantify close connections within a subnetwork, compared to randomly connected subnetworks as the null reference [10]. A unique feature of *Q_RB_* is the resolution parameter (*γ*) to control the resolution of the optimal clustering solutions [11], and *Q_RB_* is defined as


\begin{eqnarray*}
{Q}_{RB}\left( \gamma \right) = \frac{1}{{2{m}_o}}\mathop \sum \limits_c \left( {{e}_c - \gamma \frac{{K_c^2}}{{2{m}_o}}} \right)
\end{eqnarray*}

where *γ* > 0 is the clustering resolution parameter, *m_o_* is the total number of links, *e_c_* is the number of links in cluster *c*, and *K_c_* is the sum of the degree of nodes in cluster *c*. By choosing various *γ*, it allows the natural adaptation of the multiscale detection of cell clusters [5, 8, 12].

However, the multiscale cell-type architectures have been primarily explored by supervised approaches and thus guided by prior knowledge and user bias. These are exemplified by user-guided selection of several crucial parameters such as kNN and γ. These parameters often take default values such as kNN = 20 and γ = 1 or are determined through visual inspection of the clustering results across different parameter values via Uniform Manifold Approximation and Projection (UMAP) or t-distributed stochastic neighbor embedding (tSNE) embedding [5, 12]. Also, the searches for cell subtypes are often hypothesis-driven. Based on prior knowledge, supervised subclustering is performed on cell types of interest to identify subtypes at finer resolutions [2, 4, 13], but it could also shadow discovery for novel subtypes with little or no prior knowledge.

Further, *Q_RB_* suffers from the inherent resolution limit that fundamentally restricts the detection of fine clustering structures in a network. Within a network with *m* links, the resolution limit dictates the detection of closely connected subnetworks with an internal number of links, *e_c_*, only up to *e_c_ = √2 m_o_* [14], and the resolution limit persists regardless of γ [15]. The dependency of resolution limit on *m* exacerbates in many kNN networks, which often yield densely connected cell networks/subnetworks (i.e., *m_o_ ~ N_o_^2^*), and these could shadow rare but distinct cell subtypes present in the tissues.

Herein, we introduce an unsupervised multiscale clustering (MSC) approach for single-cell transcriptome analysis to resolve the issues in supervised clustering approaches and the resolution limit. Within MSC, we have developed a new cell similarity network method, the locally embedded network (LEN), to construct sparse and clustered cell networks and improve the sparsity-driven resolution limit in the modularity optimization problem. We have also implemented a new top-down clustering approach to iteratively split a parent network into more coherent and compact subnetworks, as well as eventually construct a cell hierarchy as the data-driven model of cell types and subtypes to facilitate the novel cell population discovery.

We systematically evaluated MSC’s performances. First, we comparatively tested LEN’s performance to capture ground-truth clusters under various noise sources in simulated scRNA-seq data. Then, we evaluated clustering performances by MSC on simulated data with hierarchical structures, gold-standard data with known ground-truth clusters, and cross-platform peripheral blood mononuclear cell (PBMC) data as silver-standard data to check robust performances across different sequencing platforms. Ground-truth clusters allow an objective performance comparison of MSC with widely used benchmark single-cell clustering methods, such as SNN-based Louvain clustering approaches with varying *γ* in Seurat [8], SC3 [16], and CIDR [17], which have been identified as among the best-performing single-cell clustering methods [18]. In addition, we have included the latest methods across different categories for comparisons, including adaptive kNN graph-based aKNNO [19], RaceID3 (designed for rare cell-type identification) [20], and neural network–based scCAN [21]. Then, we apply MSC to several disease scRNA-seq datasets from different tissue types to demonstrate its capacity to identify novel cell subpopulations and biological mechanisms. Overall, we present MSC as a valuable unsupervised single-cell transcriptome clustering method to understand complex cell architectures.

## Results

### Overview of MSC analysis framework

**Figure 1:**
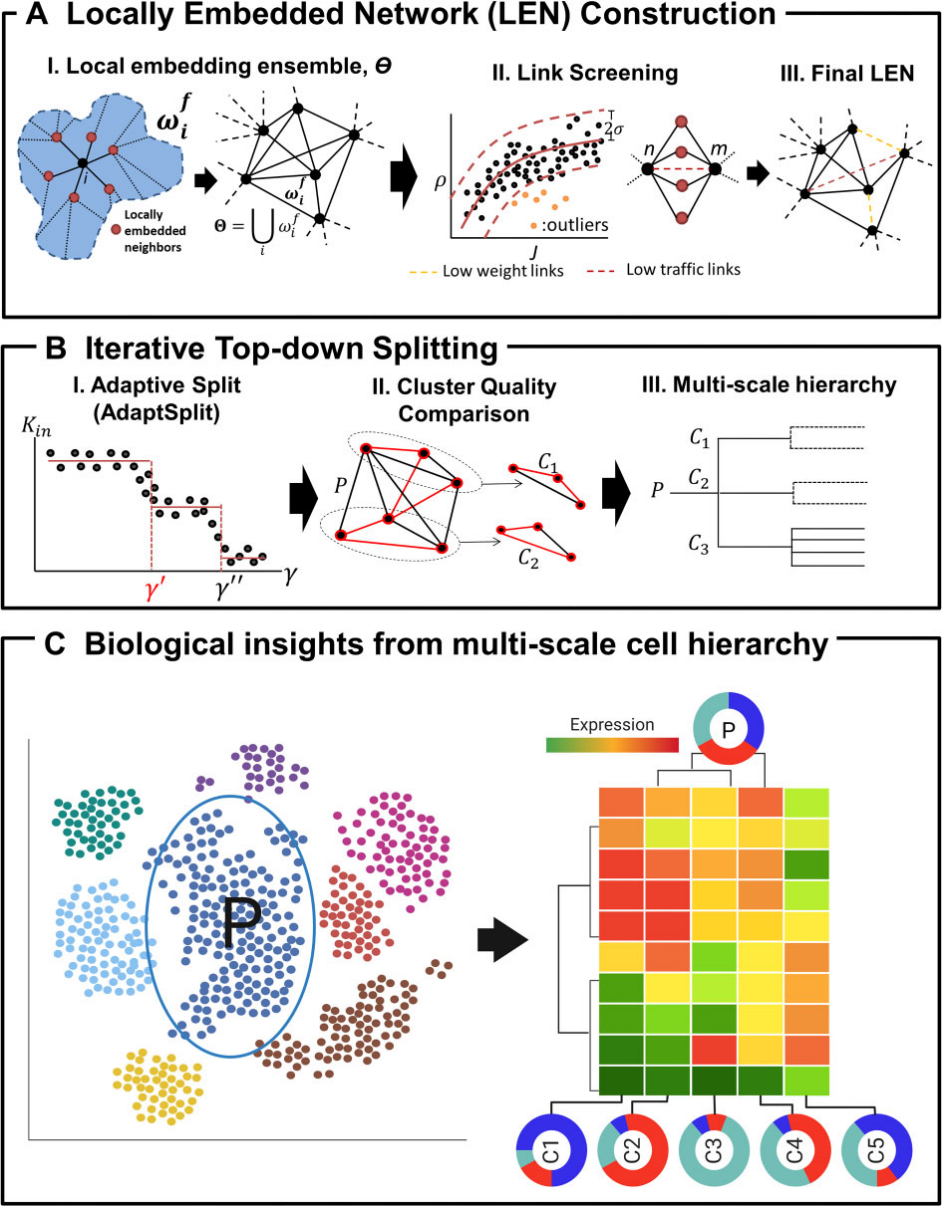
MSC workflow. (A) Locally embedded network (LEN) construction. (I) Cell-wise local embedding, ${\boldsymbol{\omega }}_{\boldsymbol{i}}^{\boldsymbol{f}}$ (left), is combined into the ensemble, ϴ (right). (II) Low-quality cell links are screened as outliers (marked orange, left) in the curve of the cell–cell correlation coefficient (ρ) vs. mutually shared gene expressions by the Jaccard index (J) and redundant links with no improvements in the mutual neighbor ratio, *M_nm_*, after link removal (marked brown, right). The filtered links (marked in brown and orange) are discarded to obtain the final LEN. (B) Iterative top-down splitting. (I) For each split, the clustering resolution parameter, γ, is tuned to detect the first break point, *γ′* (marked red), in *γ* vs. *K_in_* curve. (II) The parent cluster (P) is compared to its child clusters (C_1_ & C_2_) by cluster compactness and intracluster connectivity improvements. (III) Upon termination, MSC yields a multiscale cluster hierarchy of parents and its more compact child clusters. (C) Identification of multiscale cell subsets and cluster markers by MSC. Conditioned on each parent cluster (P, marked in the schematic tSNE plot on the left), the child clusters (C1, C2, …, C5) are compared among them to evaluate heterogeneous cell group compositions (marked by schematic pie charts) and marker genes with distinct expressions in each child cluster (illustrated by the schematic heatmap). Figure 1C created in BioRender. Song, W. (2025) https://BioRender.com/4schgiu.

MSC consists of 2 major steps, including construction of the cell similarity (also termed cell–cell interaction) network (CSN) and top-down cell clustering on the CSN (Fig. 1). First, MSC employs a novel LEN method to construct a sparse cell network without the needs to specify the kNN (Fig. 1A). For a similarity (or dissimilarity) metric of choice, LEN utilizes a graph embedding technique on a topological sphere [22] to deterministically identify the nearest neighbors (NNs) for each cell. These locally embedded nearest neighbors (eNNs) are identified by searching for high-similarity cell pairs among the cell and its eNNs without edge crossing when drawn on a sphere. In turn, the ensemble of eNNs for all cells constitutes the locally embedded neighbor network (LEN; Fig. 1A–I), followed by low-quality edge filtering through evaluating low similarity and edge centrality (Fig. 1A-II, III) (see Methods for details of LEN construction).

Then, MSC employs a top-down clustering approach, iteratively splitting a parent cell network into more coherent and compact subnetworks to produce a cell hierarchical structure of cells. While different clustering solutions may emerge at different resolutions, we aim to identify the most granular clustering solution at each split, exploring cell subpopulations at progressively finer resolutions with each resolution. Specifically, we have developed *AdaptSplit*, an adaptive clustering method to search for the most granular clustering solution at each split. The child clusters from the split are compared to the parent for assessment of improvements in compactness ($\upsilon $) and intracluster connectivity (λ) (Fig. 1B-II; see Methods for details). The iterative top-down split continues until no child cluster shows improved cluster qualities over its predecessors, completing the search for the cell hierarchy (Fig. 1B-III). The cell hierarchy then informs data-driven biological insights into the cell subsets with distinct molecular characteristics (Fig. 1C).

### Evaluation of LEN to capture cell clusters under various noises in scRNA-seq

**Figure 2:**
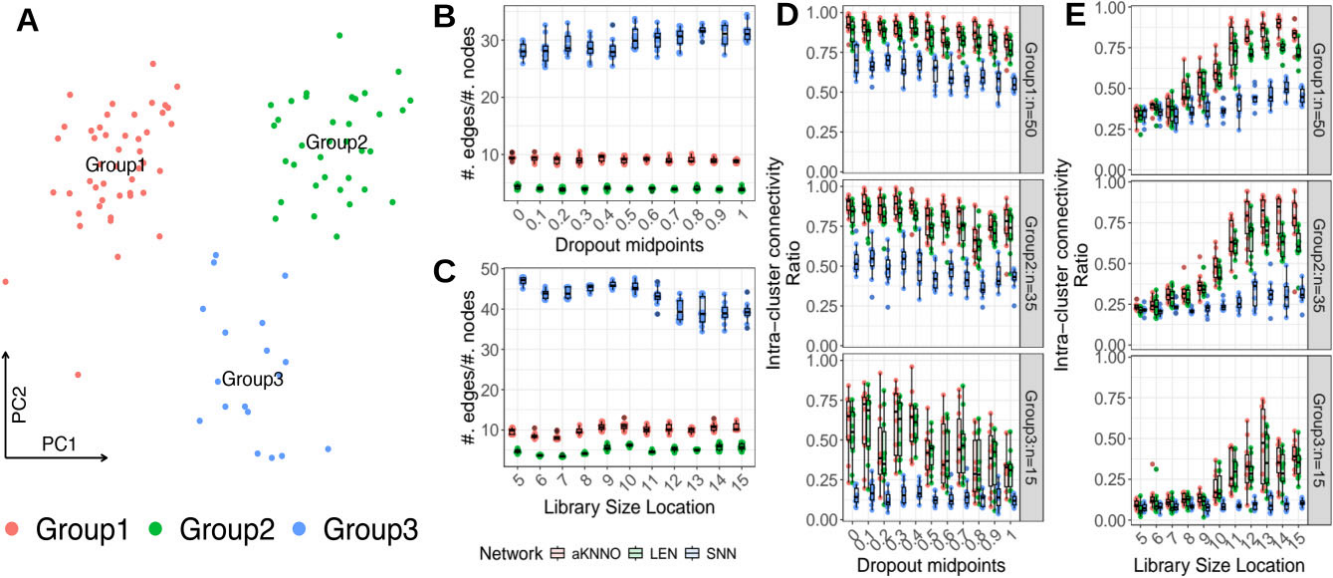
Comparative evaluation of locally embedded network (LEN) against noises in scRNA-seq. (A) Principal components (PCs) plot for first 2 PCs for an exemplary scRNA-seq data generated by splatter [24] workflow. Three clusters (groups 1, 2, and 3) of varying sizes have been generated to evaluate the impact of varying noises in various clusters. (B, C) Sparsity of various similarity networks (aKNNO: red, LEN: green, SNN: blue) across varying dropout rates (B) and library sizes (C). x-axis: dropout midpoints to define the dropout rates (in B) or library size locations to define the overall cellwise library sizes (in C) in the simulated data. y-axis: ratio of numbers of edges and nodes in each network as the measure of sparsity. (D, E) Intracluster connectivity of various similarity networks for the 3 clusters across varying dropouts (D) and library sizes (E). The intracluster connectivity is defined as the ratio of the number of within-cluster edges and the number of between-cluster edges for each cluster.

scRNA-seq data are often noisy and suffer from dropout reads and low library sizes to interfere with the underlying cellular landscapes [12, 23]. Subsequently, these noises disrupt the cell–cell connections in similarity networks and limit their capacity to capture the meaningful cell types and subtypes. Herein, we systematically evaluated the impacts of these noises on LENs and other established benchmark similarity networks, sSNN [8] and aKNNO [19], through simulated scRNA-seq data. We utilized the *Splatter* framework [24] to generate scRNA-seq data of 3 clusters of 50, 35, and 15 cells, across varying degrees of dropout rates and cellwise library sizes (Fig. 2A). Specifically, Splatter is a model-based scRNA-seq simulation framework to allow control over expected library sizes through the library size location parameter and dropout probabilities through the dropout midpoints parameter (see Methods) [24]. We varied dropout midpoints in [0,1] to adjust dropout rates and library size locations in [6, 16] to adjust the overall read depths for the simulated data.

First, we evaluated the impacts of the noises on the resulting network sparsity, as the ratio of numbers of edges and nodes. Sparsity directly impacts the inherent resolution limits to detect clusters in networks [14], and we observed that LEN consistently produced the sparsest networks across all ranges of the noise parameters (Fig. 2B, C).

In tandem, we observed that LEN consistently captured the true clusters with varying sizes across broad windows of the noise parameters. Using intracluster connectivity (the ratio between within-cluster and between-cluster edges) as the measure of preserving the true clustering structures in these networks [25], we observed that aKNNOs and LENs showed comparable performances and outperformed SNNs across all parameter ranges (Fig. 2D, E). We also observed that the smaller cluster (i.e., group 3 in Fig. 2A) was more severely penalized by increasing noise levels in all networks. Particularly, the impacts of library sizes were more visible than the dropout rates where library size location >10 served as the transition point to mark the detection limits for the true clusters (Fig. 2E).

Overall, we observed that LEN is the sparsest similarity network that can effectively capture the true clustering structures across a broad spectrum of noises in scRNA-seq. We also remark that aKNNO has been effective in capturing the true clustering structures, but at the expense of higher edge densities that are 5- to 10-fold greater than LEN.

### Performance evaluation on simulated data with cluster hierarchies

Simulated data are useful to evaluate performances of clustering methods by providing the ground-truth clusters and gain insights on how these methods behave under different scenarios by varying noises, cluster sizes, and hierarchies [26]. However, there are currently no tools to simulate single-cell sequencing data with careful controls over hierarchical structures and noise parameters. To mitigate this, we utilized the multivariate Gaussian model, $X = N( {\mu ,\Sigma } )$, with Gaussian noises, $\epsilon $, as the stochastic data generator, $X^{\prime} = X + \epsilon $. This framework allows us to instill various clustering structures, including hierarchies, by specifying the covariance matrix ($\Sigma $) with a higher intracluster covariance than the intercluster covariance, and they have been successfully utilized in our previous study [26].

**Figure 3:**
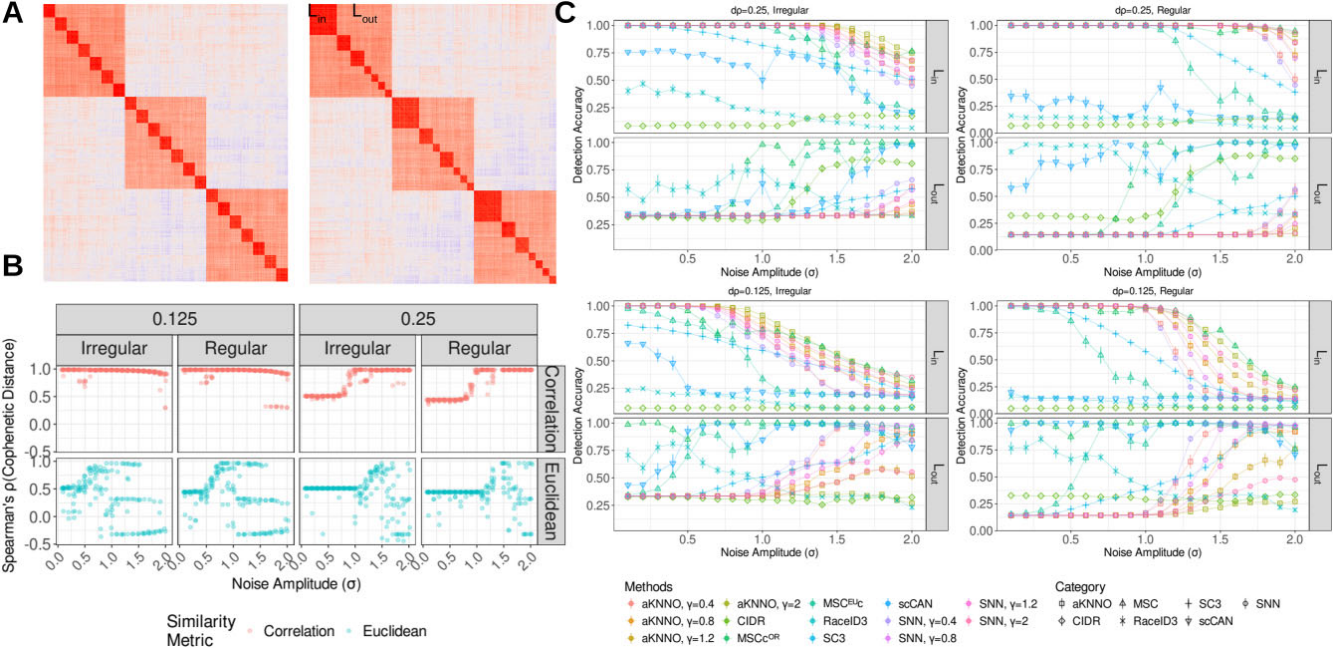
Evaluation of hierarchy detection in simulated datasets. (A) Heatmaps of correlation coefficients among the cells from the simulated data. These reflect the ground-truth hierarchies for regular (left) and irregular (right) size clusters. The inner layer of coherent clusters (L_in_) and the outer layer of less coherent clusters (L_out_) are labeled respectively. (B) Cophenetic distance between ground-truth hierarchy and MSC-inferred hierarchy using Pearson’s correlations and Euclidean distances. (C) Detection accuracy to identify clusters in L_in_ and L_out_ in different scenarios. Different clustering methods are marked by unique colors and categories by shapes.

Utilizing $X^{\prime}$, we simulated stochastic data with a 2-layer hierarchical structure in which the more correlated inner layer (L_in_) is nested in the less correlated outer layer (L_out_) (Fig. 3A). Two structural scenarios were considered: (i) a 2-layer clustering structure with regular cluster sizes to mimic cluster hierarchy (left, Fig. 3A) and (ii) a 2-layer clustering structure with irregular cluster sizes (right, Fig. 3A). The data were simulated with varying noises amplitudes (σ) and intracluster correlations at different increments (Δρ = ρ_in_ − ρ_out_) at Δρ = 0.125 and 0.25 as the factors shadowing the true clustering structures (see Methods for details). Then, we performed MSC with Pearson’s correlations across the variable genes (MSC^COR^) and Euclidean distances in variable PCs (MSC^EUC^) along with other benchmark methods.

**Figure 4:**
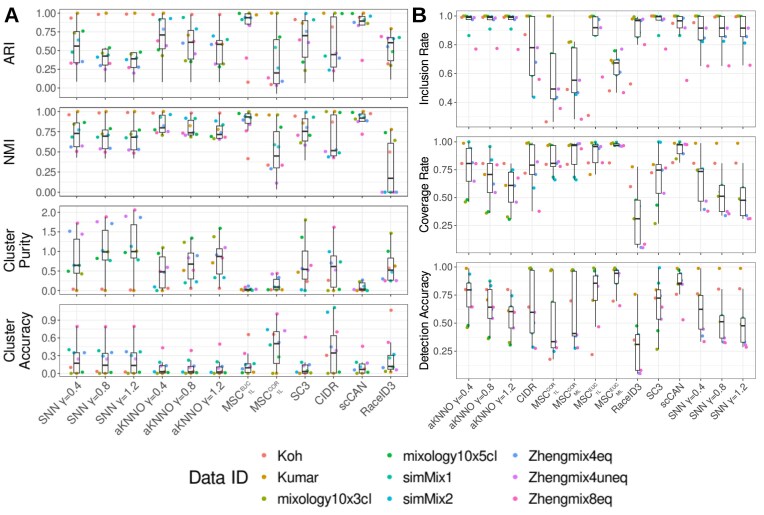
Evaluation of various single-cell clustering methods to detect ground-truth clusters in the pipeComp dataset. (A) Evaluation of agreements between the discrete clusters from various methods (on the x-axis) and the ground-truth clusters (labeled in different colors, see legend below) by Adjusted Rand Index (ARI), Normalized Mutual Information (NMI), cluster purity, and cluster accuracy. (B) Evaluation of individual clusters from different clustering methods to reproduce the ground-truth clusters by inclusion rate, coverage rate, and detection accuracy. Each dot is a ground-truth cluster; different colors remark different datasets.

**Figure 5:**
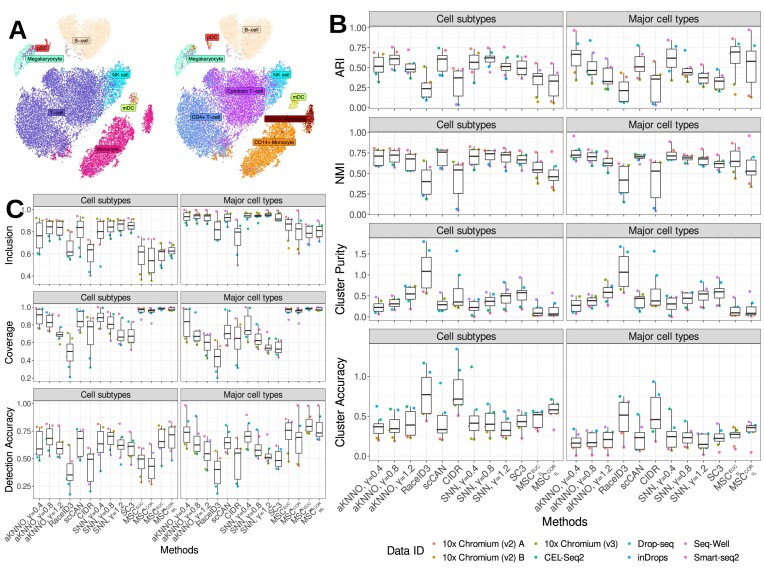
Evaluation of clustering performances in PBMC scRNA-seq across different single-cell RNA sequencing platforms from Ding et al. [27]. (A) tSNE plots of the Harmony-integrated [28] PBMC single-cell transcriptome across different sequencing technologies (10x Chromium (v2/v3), CEL-Seq2, Drop-seq, inDrops, Seq-Well, and Smart-seq2) and technical replicates (10x Chromium (v2), A and B). Major cell types (left) and subtypes (right) are shown. (B) Performance evaluations of single-cell clustering methods yielding non-overlapping discrete partitions to predict major cell types and subtypes in A. Different colors correspond to different sequencing technologies and technical replicates. (C) Performance evaluations of the single-cell clustering methods yielding overlapping and non-overlapping solutions.

Comparing MSC^COR^ to MSC^EUC^, MSC^COR^ outperformed MSC^EUC^ with higher cophenetic correlations and detection accuracies to identify the ground-truth hierarchies (Fig. 3B, C). Regardless of Δρ, one distinctive difference between the similarity metrics is the low cophenetic correlations for higher noises (σ ≥ 0.75) for the results from MSC^EUC^, compared to the results from MSC^COR^. We remark that this is in contrast to the outstanding performance of MSC^EUC^ over MSC^COR^ from other gold-standard scRNA-seq data in the later sections (Figs. 4, 5). Knowing that Pearson’s correlation directly estimates the underlying covariance structure in the multivariate Gaussian *X′*, we suspect that this has been beneficial to the outstanding performance of Pearson’s correlation in the simulated datasets.

With the right choice of the similarity metric, we observed that MSC was able to capture the full hierarchy at different noise levels. Utilizing cophenetic correlations between MSC-inferred and ground-truth hierarchies to evaluate the concordances at individual cell levels (see Methods), MSC^COR^ showed outstanding performances to detect the full hierarchy across all noise levels at the low Δρ = 0.125 and across higher noises (σ ≥ 1) at the high Δρ = 0.25 with high cophenetic correlations over 0.9, compared to MSC^EUC^ (Fig. 3B).

At the cluster level, MSC^COR^ outperformed the other benchmark methods in detecting the ground-truth clusters in both layers simultaneously. We utilized detection accuracy for L_in_ and L_out_ to check the overall detection of ground-truth clusters at different layers separately. For data generated with Δρ = 0.125, all clustering methods captured the full hierarchies at lower noise levels, followed by missing the detection of clusters at L_in_ at higher noise levels (bottom, Fig. 3C). These suggest that the higher noises disrupts the ground-truth hierarchy to blend the smaller clusters at L_in_ into the larger clusters at L_out_, and this pattern was commonly observed for all clustering methods. Nevertheless, MSC^COR^ was among the methods that captured the most clusters at L_in_ while detecting almost all clusters at L_out_ across all noise levels. These yielded the noise window, 0 ≤ σ ≤ 0.75, that MSC^COR^ could detect the clusters at both of L_in_ and L_out_, and MSC^COR^ was the only method that can detect the full hierarchy.

For data generated with Δρ = 0.25, it revealed another unique pattern that the clustering methods only detect ground-truth clusters at L_in_ at lower noises, followed by detecting both layers at the higher noises (top, Fig. 3C). Of them, MSC^COR^ was among the methods that detected the most ground-truth clusters at L_in_ while managing to detect meaningful ground-truth clusters at L_out_ for σ ≥ 1.25. We observed similar patterns of shifting noise windows to identify the ground-truth clusters at both levels by Δρ through evaluating the clusterability of MSC-inferred clusters with the Phiclust framework [29] (see Methods; Supplementary Fig. S1). Overall, these suggest that MSC^COR^ missed the higher-order structure in L_out_ at low noises when a more distinctive hierarchical structure is present with larger Δρ = 0.25, and in this case, the larger noises facilitated the realization of the higher-order structure. Together, we observed that MSC^COR^ could detect the clusters at both of L_in_ and L_out_ in 0.9 ≤ σ ≤ 1.2, and MSC^COR^ was the only method to detect the full hierarchy at some noise windows.

Also, regularities of ground-truth clusters significantly affected the hierarchy detection. For most clustering methods, regular cluster sizes in L_in_ expanded the noise windows under which they are accurately detected (Fig. 1C), compared to the irregular cluster sizes.

On the other hand, none of the resolution-based clustering with aKNNO or SNN graphs was capable of detecting both layers simultaneously. Regardless of different γ values, these methods were not able to capture the clusters at L_out_ for σ ≤ 1 (Fig. 1C). Rather, higher γ imposed lower detection accuracies for clusters at L_out_ for σ > 1, and similar results were observed for ∆ρ = 0.125 and 0.25.

We observed similar qualitative results from a benchmark scRNA-seq of 8,381 PBMCs from a healthy donor from 10x website (see Availability of Source Code and Requirements). Using the annotated cell types as silver-standard ground-truth clusters, the multiscale clustering results, $MSC_{ML}^{COR}$and $MSC_{ML}^{EUC}$, consistently captured the most similar clusters at all hierarchical levels and within different major immune types, compared to other methods (Supplementary Figs. S2, S3). In contrast, the SNN- and aKNNO-based clustering with various resolutions emphasized detection of cell subtypes at the third level and failed to realize more granular structures despite the varying resolutions (see Supplementary Results for details).

Overall, the simulated study allowed exploring various scenarios across varying noises, cluster coherence, and presence of hierarchical structures. The results demonstrate the advantages in MSC for improved detection of clusters and hierarchy compared to benchmark methods. The simulation study also outlines several clear limitations. At certain noise windows, MSC failed to detect the hierarchical structure. When noise levels are relatively low (σ ≤ 1), all clustering methods, including MSC, tend to detect the more correlated inner clusters at L_in_. On the other hand, larger noise levels (σ ≤ 1) tend to favor the detection of the less correlated outer cluster at L_out_. These suggest the roles of noises in determining detectable clusters and warrant further studies.

### Cluster compactness, *υ*(*α*), is instrumental to probe subclusters

We observed that cluster compactness effectively facilitates identification of meaningful subcluster structures in the simulated datasets. Using the Phiclust framework [29], we checked the clusterability of the parent clusters in L_out_. If detected, these parent clusters should yield a significant Phiclust score with larger compactness compared to the child clusters in L_in_. Testing this for simulated data from Δρ = 0.25 with regular clusters, it indeed showed that the detected parent clusters by MSC^COR^ showed larger compactness with significant clusterability (i.e., $\phi $> 0.9)[29] than the respective child clusters with unsignificant clusterability with $\phi $~ 0 (Supplementary Fig. S4).

Within MSC, we utilized the cluster compactness measure, $\upsilon ( \alpha ) = \overline {SPD} /log{( {{N}_c} )}^\alpha $, to determine meaningful subcluster structures, where $\overline {SPD} $ is the average of shortest path distances of all cell pairs in a network, *α* is the compactness scaling parameter, and *N_c_* is the number of nodes in cluster *c* [30]. α′ values at which the parent and its child compactness coincides (i.e., *υ_parent_*(*α*′) *= υ_child_*(*α*′)) serve as the break points that, for α < α′, the parent clusters are deemed more compact than the child clusters, and for α > α′, the child clusters are more compact than the parent clusters [30]. Utilizing the simulated data with different cluster sizes and hierarchies, we observed that these breakpoints varied across different cluster coherence and structures. More coherent and regular cluster sizes yielded higher α′ (see Supplementary Fig. S5; see Supplementary Results for details). Overall, we observed that α′ reflected structural characteristics in underlying clusters.

### Performance evaluation with gold-standard data

We collected a number of gold-standard datasets generated from independent studies, whose ground-truth clusters are known through model simulation under various scenarios, fluorescence-activated cell sorting (FACS) cell populations, and a different ratio of mRNA mixtures from distinct cell lines [31, 32] (Table 1). Using the ground-truth clusters, we sought to evaluate if the first split by MSC can effectively distinguish the major ground-truth clusters. To this end, we identified the first split clustering by MSC using Pearson’s correlations on the variable features ($MSC_{1L}^{COR}$) or Euclidean distances on the principal components ($MSC_{1L}^{EUC}$) and compared these splits to the ground-truth clusters by various cluster quality metrics. We utilized Adjusted Rand Index (ARI) [33] and Normalized Mutual Information (NMI) [34], measuring the similarity between the ground-truth clusters and computed clusters as discrete partitions (upper panels, Fig. 4A).

**Table 1:** List of gold- and silver-standard datasets with known clustering structures

Dataset	# features	# cells	Protocol	Description
Koh	33,922	531	SMARTer	9 FACS purified differentiation stages
Kumar	41,930	246	SMARTer	Mouse embryonic stem cells (ESC) cultured in 3 different conditions
Zhengmix4eq	10,434	3,994	10x	Mixtures of FACS-purified PBMCs
Zhengmix4uneq	11,369	6,498	10x	Mixtures of FACS-purified PBMCs
Zhengmix8eq	10,600	3,994	10x	Mixtures of FACS-purified PBMCs
mixology10×3cl	16,208	902	10x	Mixture of 3 cancer cell lines from CellBench
mixology10×5cl	11,786	3,918	10x	Mixture of 5 cancer cell lines from CellBench
simMix1	3,696	2,500	10x-based	Simulation of 10 human cell subpopulations
simMix2	8,893	3,000	10x-based	Simulation of 9 mouse cell subpopulations

With the perfect agreements corresponding to 1 in these measures, ARI and NMI showed that $MSC_{1L}^{EUC}$ and neural learning–based scCAN were the top-performing methods to capture the ground-truth partitions in these data, followed by aKNNO-based Louvain clusters at different resolutions. We have calculated entropy-based measures such as cluster purity and accuracy [35] (lower panels, Fig. 4A) to evaluate if the computed clusters are composed of unique ground-truth clusters (i.e., purity) or if the ground-truth clusters are composed of unique computed clusters (i.e., accuracy). While the optimal clusters correspond to 0 in the entropy-based measures, we observed that $MSC_{1L}^{EUC}$ and scCAN were the top-performing methods again. We remark that $MSC_{1L}^{COR}$ exhibited some of the best cluster purity with poor accuracy, indicative of overclustering. Conversely, SC3, SNN, and aKNNO clusters exhibited some of the best accuracy with poor purity, indicative of underclustering.

Given that MSC multiscale clustering yields overlapping clusters, we adopted performance metrics capable of handling the overlaps (Fig. 4B). To this end, we adopted the inclusion rate (IR), equivalent to the precision measure showing correctly classified cells in an inferred cluster; coverage rate (CR), equivalent to the recall measure showing correctly classified cells in a ground-truth cluster; and detection accuracy (DA), equivalent to the accuracy measure, to identify the best match between a ground-truth cluster and a inferred cluster [36] (see Methods for details). Overall, the multiscale clustering results from the Euclidean distances ($MSC_{ML}^{EUC}$) were among the best-performing methods with improved DA and CR over the first split, $MSC_{1L}^{EUC}$, while decreasing the IR. These imply that multiscale clustering identifies more accurate and correct clusters close to the ground-truth clusters, while the decreased IR is attributed to the increased numbers of parental clusters, including members of multiple ground-truth clusters.

In contrast to the simulated data by the Gaussian multivariate generator, the correlation-based MSC results, $MSC_{1L}^{COR}$ and $MSC_{ML}^{COR}$, underperformed in comparison to the Euclidean-based MSC results. While the correlations were calculated across the variable genes over the cells, the Euclidean distances were calculated within the top 20 principal components from the variable genes. These imply that the dimension reduction through PCA is the more effective approach to cluster the cells, avoiding negative impacts by single-cell specific noises. On the other hand, the correlation-based results were prone to these noises.

Further, we observed LENs were consistently sparse across all gold-standard datasets. The sparsity of a network can be formulated by the relationship, *m = c_s_N_o_*, where *m* is the total number of links, *N_o_* is the number of cells, and *c_s_* is a scaling factor to define the network sparsity. From the gold-standard datasets, LENs showed 3 ≤ *c_s_* ≤ 5. On the contrary, SNN networks showed 28 ≤ *c_s_* ≤ 40, indicating that LENs are substantially sparser than the SNN networks to facilitate the small yet meaningful cluster detection (Supplementary Fig. S6).

### Performance evaluation with silver-standard data in PMBC datasets across different sequencing platforms

We comparatively evaluated MSC with other single-cell clustering methods to identify meaningful cell types and subtypes from different sequencing technologies. We utilized the single-cell transcriptomes of PBMCs across different sequencing platforms, including 10x Chromium (v2 and v3), CEL-Seq2, Drop-seq, inDrops, Seq-Well, and Smart-seq2, across technical replicates from 10x Chromium (v2) from Ding et al. [27] (Fig. 5A, B). We performed the clustering analyses for each platform per the technical/biological replicate to test if MSC and other clustering methods can robustly detect different cell types and subtypes. First, we tested the first layer split in MSC from Euclidean distances and Pearson’s correlations ($MSC_{1L}^{EUC}$, $MSC_{1L}^{COR}$) in comparison to the other clustering methods (Fig. 5C).

As expected, $MSC_{1L}^{EUC}$ and $MSC_{1L}^{COR}$ tend to detect the major cell types better than the subtypes, and they demonstrate that the first split in MSC detects the coarse-grained clustering solutions in the data across different platforms. Also, we observed slightly better performance of $MSC_{1L}^{EUC}$ over $MSC_{1L}^{COR}$. Compared to other benchmark methods, we observed $MSC_{1L}^{EUC}$ and $MSC_{1L}^{COR}$ were among the best-performing methods to detect the major cell types, while the cell subtype detections were suboptimal and showed similar performances to aKNNO- or SNN-based clustering at low resolution (γ = 0.4).

To evaluate the multiscale clusters in MSC ($MSC_{ML}^{EUC}$,$MSC_{ML}^{COR}$), we employed the DA, CR, and IR metrics capable of handling non-overlapping clusters (Fig. 5D). Compared to the first splits in the multiscale clusters in MSC, multiscale clustering improved the detection accuracy of the major cell types and subtypes in both metrics (bottom, Fig. 5D), indicating the multiscale search strategy succeeds in discovering more ground-truth clusters. These are also indicated in the high coverage rates from the MSC clusters (middle, Fig. 5D), indicating that the ground-truth clusters were correctly classified into unique clusters. On the other hand, the inclusion rates were suboptimal for MSC to indicate the computed clusters contain different ground-truth clusters (top, Fig. 5D). This is expected for MSC as the coarse-grained, parent clusters in the multiscale search inevitably include the larger clusters housing multiple ground-truth clusters.

Overall, these trends were robustly observed across different platforms and replicates for all clustering methods, including MSC. These indicate that MSC can robustly detect the multiscale cell-type landscapes in different experimental and technical settings.

### Applications to influenza- and COVID-19–infected PBMC scRNA-seq: MSC identifies novel *CRBN/RBX1*-high platelet subpopulations in severe COVID-19

To assess the utility of MSC to study cellular landscapes in infectious diseases, we processed and analyzed a single-cell transcriptome of 62,301 cells from 20 PBMC samples, comprising 5 influenza-infected patients, 11 COVID-19–infected patients with varying range of severity, and 4 healthy controls from Lee *et al*. [1] (see Methods for data processing details).

**Figure 6:**
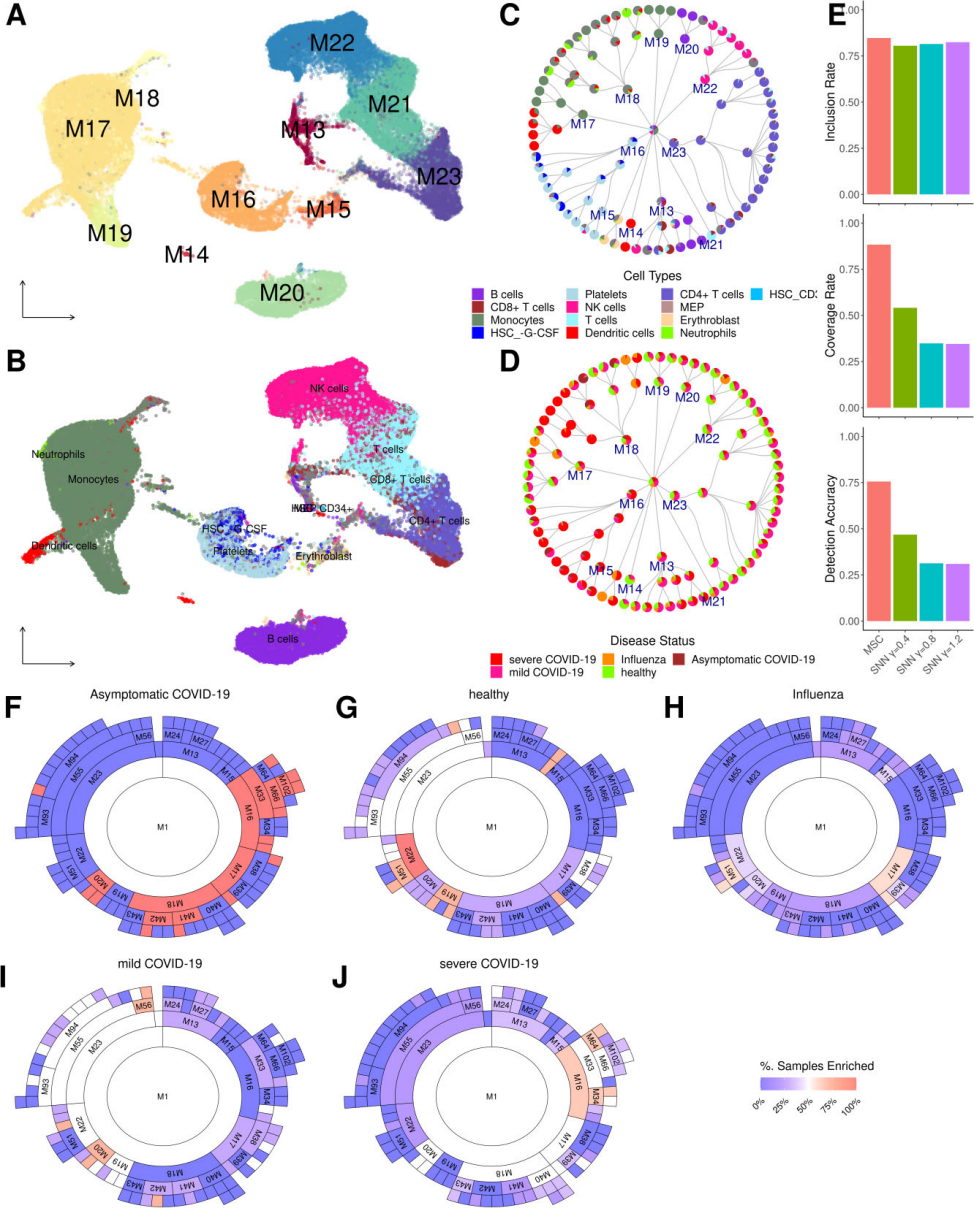
Application of MSC to scRNA-seq of PBMCs from influenza-infected, COVID-19–infected, and healthy control samples. (A, B) UMAP plots showing the first split clusters by MSC (A) and inferred cell types (B). The cell-type colors are specified in the legend in C. (C, D) MSC cluster hierarchy plots: each node shows inferred cell-type composition (C) or sample compositions (D). (E) Performance evaluation of MSC- and SNN-based clustering at different resolutions. Top: inclusion rate. Middle: coverage rate. Bottom: detection accuracy. (F–J) Sunburst plots showing MSC cluster branches enriched for asymptomatic patients with COVID-19 (F), healthy controls (G), patients with influenza (H), patients with mild COVID-19 (I), and patients with severe COVID-19 (J).

MSC clusters systematically identified several branches of immune/blood cell types associated with influenza and COVID-19 infections. Using the finalized cell-type annotations (Fig. 6B; see Methods for cell-type annotations; Supplementary Data S1A), the MSC cluster hierarchy (Supplementary Data S1B, C) captured most of the major cell types in the clusters at the first split, and the child clusters subsequently compartmentalized into more distinct immune cell subtypes (Fig. 6A–C), characterized by enrichments of different disease conditions (Fig. 6D). In particular, MSC outperformed SNN-based Louvain clustering at varying resolutions in detecting the annotated cell types and subtypes with greater IR, CR, and DA (Fig. 5E; Supplementary Fig. S11). We note that other benchmark methods were not successfully executed due to the requirements for large computational resources by these methods and hence were omitted in the comparisons.

Several unique cell subtypes identified by MSC were associated with severe COVID-19 samples. Many cell clusters showed preferential enrichments for individuals from specific disease conditions (Fig. 5F–J; Supplementary Data S1D). One example is the expansion of platelets in severe COVID-19 samples (Fig. 5J), comprising *CRBN/RBX1*-high (M33) and *IFITM3*-high (M34) subpopulations (Supplementary Fig. S12). Recently, lenalidomide, a CRBN/RBX1 inhibitor, has shown protective roles in multiple COVID-19–infected myeloma patients against progression into severe infections [37], which suggests the emergence of this particular platelet subpopulation may drive the disease severity in COVID-19 infection. On the contrary, *IFITM3* is an interferon (IFN)–induced antiviral protein, and its expression is shared with monocytes/macrophages. Polymorphism in IFITM3 has been associated with COVID-19 and severity [38], and its expression inhibits COVID-19 infection [38]. This suggests that M34 is a protective platelet subtype under proinflammatory environments. Overall, the MSC identified distinct platelet subtypes with functionally distinct characteristics, and these warrant further investigations for novel COVID-19 therapeutics.

### Applications to a breast cancer single-cell atlas: MSC identifies a novel protective endothelial subset in breast cancer

We expanded MSC applications to a large-scale study of breast cancer single-cell transcriptomes to explore heterogeneous tumor microenvironments and novel cell subtypes in solid tumors. Specifically, we performed MSC on a single-cell transcriptome atlas of breast cancer by Wu *et al*. [39], encompassing 26 breast cancer primary tumors of diverse subtypes by hormonal status (estrogen receptor [ER], progesterone receptor [PR] status), Her2 signaling status (Her2 amplification/deletion), and molecular PAM50 subtyping [39]. This study has identified major cell types and the subsets through adapting supervised approaches to infer known cell types by xCell [40] and subclusters within known major cell types by SNN-based Louvain clustering in Seurat (Supplementary Data S2A).

**Figure 7:**
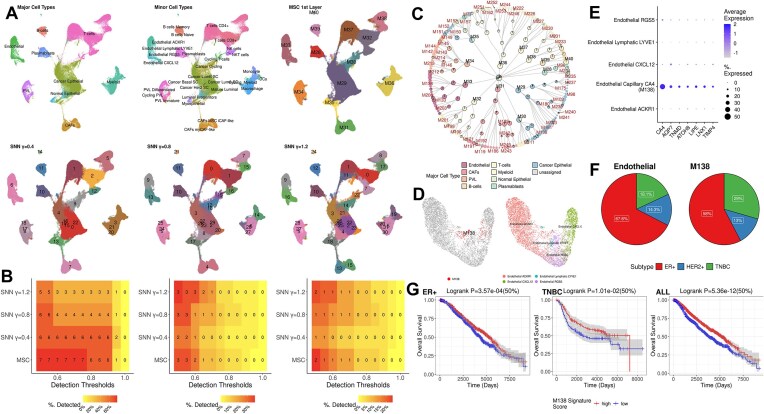
Unsupervised multiscale clustering of breast cancer single-cell transcriptome atlas from Wu et al. [39]. (A) UMAP plots to show major cell types (top left), minor cell types (top middle), first layer clustering by MSC (top right), and SNN-based Louvain clustering at γ = 0.4 (bottom left), 0.8 (bottom middle), and 1.2 (bottom right). (B) Number of detected cell types at different resolutions (left: major cell types, middle: minor cell types, right: cell subsets by supervised subclustering) by unsupervised clustering approaches (y-axis) at different detection accuracy thresholds (x-axis). (C) Hierarchy of cell clusters and subsets identified by MSC. Each pie chart shows major cell-type composition of an individual cluster, as annotated by Wu et al. [39], and the central pie chart summarizes the overall major cell-type composition in the whole dataset. MSC-unique clusters showing Jaccard index <10% with the annotated cell types and subsets, as well as clusters by SNN-based Louvain clustering at different resolutions, are labeled with red. (D) MSC identifies M138 as a unique endothelial subset (UMAP on left), compared to the annotated subsets by Wu et al. [39] (UMAP on right). (E) Dotplot of M138-specific marker genes in endothelial cells. (F) Composition of breast cancer subtypes by ER, Her2, or triple-negative breast cancer (TNBC) status in whole endothelial cells (left) and M138 (right). (G) Kaplan–Meier plots of METABRIC patients with breast cancer of different subtypes (left: ER^+^, middle: TNBC, right: the whole METABRIC cohort), stratified by the median ssGSEA score of M138-specific markers in individual transcriptome samples.

After quality control (QC; see Methods for data processing details), we processed 92,232 cells, as well as analyzed and enumerated distinct cell populations. First, we performed MSC- and SNN-based clustering at varying resolutions (γ = 0.4, 0.8, and 1.2) (Fig. 7A, B) and compared the clustering results to the annotated major cell types and subsets from the published study as the silver-standard ground-truth clusters (Supplementary Data S2B–D). Many benchmark methods could not be carried out due to their excessive memory requirements. The first-split cell clusters from MSC readily captured the major cell types without supervision, while SNN-based clustering required the fine-tuning of the resolution (Fig. 7A). Further, MSC consistently detected higher numbers of the ground-truth clusters of major cell types and subtypes, compared to the SNN-based Louvain clustering (Fig. 7B).

As the cell types and subtypes identified by Wu *et al*. [39] are primarily by supervised approaches, we anticipated that unsupervised clustering results by MSC could potentially identify novel cell subtypes, which were overlooked in the supervised approaches, and provide insights to the breast cancer biology. To this end, we leveraged the Jaccard index (JI) as a normalized overlap metric to assess MSC-unique clusters with low overlaps against the annotated cell types/subsets and the SNN-based Louvain clusters at different resolutions with JI <10% (Supplementary Data S2E, F). These yielded a large number of MSC-unique clusters, primarily as subtypes within major cell types in the cell hierarchy (Fig. 7C).

Among these, M138 captured a unique endothelial subset that was overlooked in the previous study (Fig. 6D). While the previous study identified the subsets characterized by *ACKR1, LYVE1, CXCL12* and *RGS5* (right, Fig. 6D), M138 is a unique subset of capillary endothelial cells (ECs) characterized by *CA4* expression (Fig. 7E) [41, 42] and is present in ER^+^, Her2^+^, and triple-negative breast cancer (TNBC) subtypes, with enrichment of cells from TNBC, compared to the pool of all ECs (Fig. 7F; Fisher’s exact test [FET] *P* = 8.71E-5, Enrichment fold change (EFC) = 1.62).

We observed that the presence of the M138 EC subset in breast cancers is robustly predictive of a good prognosis. To estimate the relative abundance of the M138 EC subset, we identified M138-specific marker expressions (Fig. 7E; Supplementary Fig. S13; see Methods for marker identification) and performed single-sample gene set enrichment analysis (ssGSEA) [43] as the proxy for the relative abundance of M138 ECs in the METABRIC bulk transcriptome cohort [44] (see Methods for METABRIC data processing). Stratifying patients by median M138 ssGSEA scores, stronger enrichments of M138 cells were significantly associated with a good prognosis in ER^+^, TNBC and all METABRIC cohorts with a log-rank *P* < 0.05 (Fig. 7G). We also observed that higher expressions of several M138 marker genes were significantly associated with better relapse-free survival in independent breast cancer transcriptomes from previously published studies [45] (Supplementary Fig. S14). Reported functions of the marker genes in the literature are also supportive of the protective roles of the capillary ECs against breast cancer. These include *TIMP4* (an inhibitor of capillary EC invasion [46]), *TNMD* (an angiogenesis inhibitor), *ATOH8* (transcription factor to regulate endothelial cell proliferation [47]), *AQP7* [48], and *LIPE* [49] (regulators of fatty acid metabolism).

Overall, these results demonstrate that MSC can effectively facilitate the discovery of novel cell subsets in exploratory studies, as exemplified by M138. M138 signifies a unique capillary endothelial subset characterized by *CA4* overexpression, and its presence is robustly predictive of a good prognosis in breast cancer.

### Computational complexity of MSC

We analyzed the overall computational complexity, $O( n ) \sim {n}^\eta $ (*η* is the scaling factor), of different methods through measuring the runtimes of MSC and the benchmark method scales across data with varying sizes (*n*). We curated a set of publicly available scRNA-seq data whose sizes vary from small-sized cohorts (<10,000 cells) to atlas-sized cohorts (>100,000 cells). We utilized parallel computations with 8 cores for methods with available parallel functionalities (SC3 and MSC) and assigned 8 GB of memory for each core. Overall, MSC is a scalable clustering method that analyzes small- to atlas-sized single-cell cohorts with feasible computational resources on personal machines. MSC- and SNN-based clustering were among the most scalable methods showing *η* ~ 1.3, while SC3 showed *η* ~ 2 and CIDR showed *η* ~ 2.7 (Supplementary Fig. S7A).

The memory usage was also a crucial factor for applicability. While memory usages by MSC- and SNN-based clustering scaled similarly across different datasets with tractable <50-GB usages, CIDR and SC3 failed to perform due to excessive memory usage for >10,000 cells (Supplementary Fig. S7B). With access to high-performance computing, MSC can be further parallelized to improve the overall runtime (see Supplementary Results for detailed analysis).

## Discussion

In this study, we have developed a new MSC approach. First, we introduced a novel method for constructing a cell similarity network, named LEN. LEN is a deterministic method that does not require user-defined parameters such as kNN and guarantees the generation of sparse cell networks owing to the utilization of embedding the nearest neighbors on a topological sphere, which imposes a hard upper bound on the number of links in the locally embedded network, *m_local_*, by Euler’s relation, where *m_local_* ≤ 3(*N_local_* − 2) for such embedded networks [22]. This upper bound implies the local sparsity (c*_s_^local^*) is restricted up to 3, and this translated to the global sparsity in 3 ≤ *c_s_* ≤ 5.

Such sparsity can inherently improve the cluster detection resolution limit via lowering the overall number of links (*m_o_*), restricting the detection of cell clusters with the number of internal links, *e_c_ =√2 m_o_* [14].

We also introduced a new MSC algorithm, which detects a meaningful cell cluster hierarchy in a LEN and improves detection accuracy of the underlying clustering structures in the single-cell transcriptome data. The performance of MSC was evaluated in simulated data by multivariate Gaussian models with noise. Overall, MSC outperformed other benchmark single-cell clustering methods by detecting the true clusters with greater accuracy under various scenarios simulating the presence of a cluster hierarchy, varying noise amplitudes, and irregular cluster sizes (Fig. 3).

Interestingly, MSC was the only method capable of simultaneously detecting clusters at different hierarchical layers (Fig. 3B, C). The top-down iterative clustering approach allowed detection of the nested, inner layer clusters at L_in_ after successfully detecting the outer layer clusters at L_out_. However, depending on the cluster size regularity, different windows of noise amplitudes allowed the simultaneous detection of clusters at both layers. This is in contrast to the kNN-based clustering results detecting only 1 layer of clusters, regardless of the varying cluster resolution parameter, γ. Rather, the noise amplitudes were the main determinants of the kNN-based clustering results. The lower noise amplitudes favored detection of the inner layer clusters at L_in_, and higher noise amplitudes favored the outer layer clusters at L_out_. These translated to detecting major immune cell types and subtypes in scRNA-seq of 8,381 PBMC cells, in which MSC captured the immune cell types at different hierarchy levels most accurately among the clustering methods (Supplementary Fig. S3). Overall, these exemplify the benefits of multiscale cluster detection in MSC by the top-down approach; otherwise, controlling for γ alone is not capable of exploring the cluster hierarchy.

Further, we showed that MSC consistently outperformed other benchmark single-cell clustering methods across different scRNA-seq platforms. MSC showed greater detection accuracy and concordance to the ground-truth clusters in gold-standard benchmark datasets from FACS or mRNA mixtures from different cell lines from different scRNA-seq platforms (Fig. 4), as well as PBMC scRNA-seq from different sequencing platforms (Fig. 5).

The superior performance of MSC is evident when applied to detect cell types in real-world scRNA-seq data from various diseases and tissues. Using inferred cell types as the silver standard, MSC detected the highest number of major cell types and their subtypes in PBMCs from influenza- and COVID-19– infected patients (Fig. 6) and patients with breast cancer (Fig. 7). We demonstrated that MSC is capable of identifying novel cell populations associated with various disease etiologies. From the PBMCs of influenza- and COVID-19–infected patients, MSC identified 2 platelet subpopulations expanded in patients with severe COVID-19—namely, *CRBN/RBX1*-high (M33) and *IFITM3*-high (M34) cells. Particularly, the overexpression of *CRBN/RBX1* exemplified the potential therapeutic implication of lenalidomide, a CRBN/RBX1 inhibitor, in patients with severe COVID-19, where the CRBN/RBX1 inhibitor was reported as protective against severe COVID-19 in several patients with myeloma whose standard of care included lenalidomide [37].

MSC also facilitated detection of novel cell subtypes in breast cancers. While the supervised subclustering of the endothelial cells in the published study had 4 subsets characterized by *ACKR1, LYVE1, CXCL12*, and *RGS5* expression, MSC readily identified another distinct capillary EC subset, characterized by *CA4* expression. Enrichment of the capillary EC subset was robustly associated with a good prognosis in multiple breast cancer bulk transcriptome cohorts, demonstrating the utility of MSC for novel cell subset discovery in diseased tissues.

## Conclusions

We have presented MSC as a new single-cell multiscale clustering framework that integrates an innovative algorithm for constructing cell–cell similarity networks with a multiscale clustering strategy. MSC shows superior performance over several state-of-the-art single-cell clustering methods through an objective evaluation using a broad spectrum of simulated and real-world data with ground-truth clusters. MSC is a powerful tool for advancing discoveries in disease associated cell populations using single-cell sequencing data.

## Methods

### Overview of MSC

MSC is a 2-step process consisting of cell–cell similarity network construction by LEN, followed by iterative top-down splits of the cell network to realize a hierarchy of parent and child clusters (Fig. 1).

- **LEN construction**: In many complex real-world networks, the network topologies among a node and its immediate neighbors are often planar, such as star graphs and wheel graphs [50]. Further, planarity networks are sparse networks due to the topologically enforced upper limit on the number of links, $m = 3( {N - 2} )$, where *N* = number of nodes, by Euler’s relation [50]. Taken together, this implies that the planarity constraint could be sufficient to realize the true interacting neighbors for a node and guarantee sparsity in the resulting local network. Indeed, we have translated the planarity constraint to construct gene interaction networks [30], and these networks have been validated to capture true gene interactions and facilitated discoveries of novel regulators of disease pathways such as cancers [51–53], asthma [54], neurodegenerative diseases [55–57], and infectious diseases [58]. Herein, we sought to translate the utility of the planar network to effectively construct clustered and sparse cell similarity networks.
  - Search for locally embedded neighbors for individual cells: We leveraged the planarity constraint to determine the nearest neighboring cells to construct sparse and clustered cell similarity networks. Using a cell similarity of choice, *S*, LEN first searches for *k* most similar cells (*NN_k_^i^*), $NN_k^i = {\mathrm{\{ }}j{\mathrm{|}}S( {i,j} ) \le {S}_k( i )\} $, where *S_k_*(*i*) = *k*th nearest similarity from each cell, *i*. Then, a planar maximally filtered graph (PMFG) among the cells in *NN_k_^i^* is constructed to identify a planar graph, $P_k^i$, with the maximal number of links, 3(*NN_k_^i^*-2), that maximize the overall similarity among the connected cells [22] (Fig. 1A–I). As we gradually increase *k* in $[ {3,\sqrt {{N}_o} } ]$ (*N_o_* = number of cells in the dataset), the neighbors immediately connected to *i* in $P_k^i$ saturate to a plateau at *k′* to yield the finalized nearest neighbors, $NN_{k^{\prime}}^i = N{N}^i$, as the locally embedded neighbors. In practice, we find $k^{\prime} \sim log( {{N}_o} )$ to reach the plateau. Finally, the locally embedded network of each cell, $P{^{\prime}}^i$, is realized by connecting to its embedded neighbors, *NN^i^*, and the overall locally embedded network is constructed through the ensemble across all cells, $G^{\prime} = {U}_iP{^{\prime}}^i$.
  - Low-quality link screening: As the local embedding explores directly linked cells (i.e., the first-order connections), the higher-order network structures, such as local clustering and node centralities, are overlooked in the initial network, and as a result, low-quality links to shadow the higher structures can be introduced in $G^{\prime}$. Further, scRNA-seq is often noisy and may result in introducing low-quality cell–cell links to further shadow the network topology. To mitigate these, we have implemented link screening steps to filter out links with low similarities and low centralities:

- *Low similarity screen*: The sparsity of the single-cell transcriptome is a major source of noise and is detrimental to inferring the cell clustering structure [5]. To this end, we observed the single-cell transcriptome sparsity manifested in the varying number of commonly expressed genes between 2 cells across a broad range, and this affected the pairwise cell similarities, *S_ij_*, to vary dependently on the size of commonly expressed genes (Supplementary Fig. S8). Thus, we modeled the relationship between the number of common genes and the cell–cell similarity with LOESS regression [59], as well as identified the noisy links as the outliers from the fitted curve. Specifically, we calculated the proportion of commonly expressed genes between 2 cells over the union of all expressed genes in both cells, *J_ij_*. Then, we evaluated the relationship between *J_ij_* and *S_ij_* via LOESS regression to identify the sparsity-dependent similarity thresholds as 2 standard deviations away from the fitted mean (left, Fig. 1A-II).
- *Low centrality screen*: The ratio of shared nearest neighbors between 2 cells, *M_ij_*, is a useful second-order centrality measure to evaluate the local clustering structures [60]. We calculate the *M_ij_* for all pairs of connected cells in *G′* and contest the lower quantile cell pairs by the cell–cell similarity for removal. For each contested cell pair, we evaluate if removal of the cell link improves *M_ij_*. If improved, the cell link is removed, and this removal occurs iteratively for all contested cell pairs. The cell link removal iteratively occurs for the similarity-sorted cell links (middle, Fig. 1A-II).

Altogether, the local embedding and link screening yields the finalized locally embedded network (LEN), *G_o_*.

- **Iterative top-down clustering**: The clustering structure in *G_o_* is probed by iteratively splitting parent networks into several child clusters with improved cluster qualities, including connectivity (i.e., coherent clusters) and compactness (i.e., tightly connected clusters). The iterative splits terminate when no further child clusters are discovered with improved cluster qualities and eventually identify a cell hierarchy of parent and child clusters as the data-driven model of cellular architecture in the single-cell transcriptome.

Adaptive network split ( *AdaptSplit* ) to search for granular clustering solutions: Each split purposely searches for the most granular clusters so that the child clusters represent the immediate subtypes of their parent cell type. These granular clusters may be defined at varying resolutions, dependent on the parent network’s topology. To address this, we devised *AdaptSplit* method to adaptively search for the granular clustering solution. Specifically, *AdaptSplit* first identifies clustering solutions in *γ′* ϵ (0,2] on a parent network, *G_o_*(*V_o_,E_o_*), by Leiden’s clustering [61]. The range of *γ′* is purposely set to explore the clustering solutions around the neutral resolution, *γ′* = 1 [10, 11], and include widely used *γ′* ≤ 1.2 in single-cell clustering [8, 12].

We hypothesized that a stable, granular clustering solution should maintain stable intracluster connectivity at low resolutions (i.e., low *γ′* values). To test this, we examined the overall intracluster connectivity, ${K}_{in} = \mathop \sum \limits_{i,j \in {\Theta }_c} {A}_{ij}$, where *A_ij_* = 1 if *i* and *j* are connected for a clustering solution by Louvain clustering at *γ′*,$\Psi ( {\gamma = \gamma ^{\prime}} ) = \{ {\Theta }_c|{\Theta }_c \subseteq {V}_o\} $ with the disjoint conditions (${\Theta }_c \cap {\Theta }_{c^{\prime}} = \emptyset ,c \neq c^{\prime}$ and ${U}_c{\Theta }_c = {V}_o$), to maintain stable values for a range of *γ′* values. Typically, more fragmented and smaller clusters yield smaller *K_in_*, and often, stable clustering solutions manifest as stable *K_in_* across a certain range of $\gamma ^{\prime} \le \gamma \le \gamma ^{\prime\prime}$, at the break points, *γ′* and *γ″* (Fig. 1B–I). The break points are systematically identified by logistic regression to fit step functions incorporating the discrete *K_in_* values at different *γ* regimes with the *rpart* R package (v4.1.19). The first regime, *γ* < *γ′* (highlighted in Fig. 1B–I), is identified as the stable clustering solutions with granular clusters, and the clustering solution with median resolution in the regime, *γ_f_*, is selected as the final clustering result for *AdaptSplit*.

Comparative evaluations of child clusters to their parent clusters for cluster quality improvements: Then, the child clusters are compared to their respective parent cluster for improved cluster qualities. This comparison assumes that the split is meaningful only if it yields more well-defined clusters than the parent cluster, and this rationale serves to determine the termination when no further improved child clusters are detected. Specifically, we utilize (I) compactness and (II) intracluster connectivity as the cluster quality metrics:

- Compactness comparison: We have previously developed Multi-scale Embedded Gene co-Expression Analysis (MEGENA), which utilizes an iterative top-down clustering approach on planar gene networks [30]. Within MEGENA, we established a cluster compactness measure, $\upsilon ( \alpha ) = \overline {SPD} /log{( {{N}_c} )}^\alpha $, where $\overline {SPD} $ is the average of the shortest path distances of all cell pairs in a network, *α* is the compactness scaling parameter, and *N_c_* is the number of nodes in cluster *c*. When comparing compactness of child clusters to the parent cluster, we showed that $\upsilon ( \alpha )$ can effectively identify compact child clusters and detect a biologically meaningful cluster hierarchy of parent and child clusters [30]. However, its direct translation to LEN is limited as α varies in a narrow range for planar networks [30, 62]. To this end, we adapted the compactness measure by fine-tuning *α*. In MSC workflow, *α* serves as the scaling parameter for $\overline {SPD} $ and determines the role of cluster sizes in calculating the compactness. To identify the suitable *α* for a given network, we randomly sample 100 subnetworks by propagating 3-layer neighborhoods of 100 randomly chosen nodes. Standardizing $\upsilon ( {{\alpha }_o} ) = 1$ as the normalized compactness where *α_o_* serves as the reference scaling parameter, we can derive the expression for the reference scaling parameter as ${\alpha }_o = log( {\overline {SPD} } )/log( {log( {{N}_c} )} )$. In an *N_c_* versus *α_o_* plot, *α_o_* converged toward a constant value <2 (See Supplementary Fig. S9) in most cases, and this convergent value was used as the compactness scaling parameter for parent–child cluster comparisons.
- Intracluster connectivity comparison: In addition to the compactness comparison between the parent and child clusters, we evaluated the significance of intracluster density among the child clusters to ensure probing for coherent clustering structures. Within each parent cluster, *p*, the intracluster connectivity of each child cluster, *c*, can be defined as ${\lambda }_c = e_{cc}^p/e_c^p$, where $e_c^p$ is the number of links connected to any cells in *c*, and *e^p^_cc_* is the number of links connecting cells within cluster *c*.

We evaluated the statistical significance of ${\lambda }_c$ by randomly permuting 10% of cells across different child clusters 100 times and calculated the permuted intracluster density $\lambda _{cc}^{\prime}$ as the random reference values to calculate the significant *P* value. With the density *P* < 0.05, the child clusters were identified as significantly coherent.

**scRNA-seq simulation using the Splatter framework:** Splatter is a model-based scRNA-seq simulation framework that allows controlling various noise sources through parametrized models [24]. Within this framework, the library size is modeled through log-normal distribution, $lnN( {{\mu }_i,{\sigma }_i} )$, where *μ_i_* = library size location, and *σ_i_* = library size scale. We varied *μ_i_* in [6, 16] to shift the overall dropout rates while fixing *σ_i_* at the default value of 0.2. We have also experimented with varying dropout rates. Splatter models the dropout probability by a logistic function, ${\pi }_{ij} = 1( {1 + exp( { - k( {ln( {{\lambda }_{ij}} ) - {x}_0} )} )} )$, where *x*_0_ = dropout midpoint, *k* = dropout shape. We varied *x*_0_ in [0,1] while fixing *k* = −1 to control the overall dropout rates in the simulated data. While varying the library size locations, we fixed *x*_0_ at the default value, 0. Likewise, we fixed *μ_i_* at the default value of 11 while varying the dropout rates. For each unique set of parameters, we generated 10 replicates to ensure the robustness of the findings.

### Disease group enrichment analysis

We performed FET to evaluate enrichment of individual cell clusters in individual samples. A sample was deemed enriched for a cell cluster if the respective false discovery rate (FDR)–adjusted FET *P* (FET FDR) < 0.05. Then, for each disease condition and each cell cluster, we calculated the proportion of samples showing the enrichments and labeled cell clusters with at least 50% of samples from a respective disease condition as enriched.

### Data simulation

We generated simulated data using multivariate Gaussian model, $X \sim N( {\mu ,\Sigma } ),X \in {R}^N$, where $\mu = E( X )$ is the N-dimensional mean vector, and ${\Sigma }_{ij} = E( {( {{X}_i - {\mu }_i} )( {{X}_j - {\mu }_j} )} )$ is the covariance between *i*th and *j*th values in *X*. Then, we added data Gaussian noises ($\epsilon \sim N( {\mu ,0} )$) to this model; hence, $X^{\prime} = X + \epsilon $. Throughout the simulations, we also imposed ${\Sigma }_{ii} = 1$ and ${\mu }_i = 0$ for all *i* to ensure the covariance becomes synonymous with the correlation, $\rho $.

In this formulation, we have customized the correlation matrix to impose several clustering scenarios in the simulated data.

Two scenarios include:

- A hierarchical clustering structure of regular cluster sizes (left, Fig. 3A): We defined 2 layers of clustering structures by imposing different correlation strengths at different layers. Specifically, we started by defining 21 seed clusters of size 50, constituting the inner layer clustering structure (L_in_), with an intracluster correlation, ρ_in_. Then, we adjoined 6 seed clusters to construct the outer layer clustering structure (${L}_{out}$), with a weaker intracluster correlation, ${\rho }_{out}$ with ${\rho }_{in} > {\rho }_{out} > 0$. The intercluster coefficients were fixed at 0. We explored 2 different subscenarios by controlling $\Delta \rho = {\rho }_{in} - {\rho }_{out}$ at 0.125 and 0.25, to simulate different definitions in the hierarchy. Having defined the hierarchical correlation matrix, we varied the amplitude of the Gaussian noises via $\sigma \in [ {0.1,2} ]$.
- A hierarchical clustering structure of irregular cluster sizes (right, Fig. 3A): Similar to scenario I, we imposed a 2-layer hierarchy with ${\rho }_{out}$ = 0.125 and 0.25, where the seed clusters were heterogeneous in size at *L_in_*, including 12 clusters of size 25, 6 clusters of size 50, and 3 clusters of size 100. At *L_out_*, we imposed the higher-layer clustering structure by merging 4 seed clusters of size 25, 2 seed clusters of size 50, and 1 seed cluster of size 100 with ${\rho }_{out}$. Similar to scenario I, we generated $\Delta \rho = 0.125,0.25$ with varying Gaussian noise amplitudes, $\sigma \in [ {0.1,2} ]$.

For each set of parameters, we generated 10 random replicates, across 500 features. While each scenario generated data across ~1,000 cells, the number of features was deliberately selected to be much smaller than the number of cells, as observed in many scRNA-seq studies [5]. These simulations were performed using the *MASS* R package (v7.3–57).

### Evaluation metrics

As MSC yields overlapping clusters from its parent–child cluster hierarchy, we evaluated the agreements of clustering results with the true clusters by adopting the evaluation metrics for overlapping clusters. Traditionally, for clustering results, $\Psi ^{\prime} = \{ \Theta _i^{\prime}|i = 1, \cdots ,k^{\prime}\} $, and ground-truth clusters, ${\Psi }^o = \{ \Theta _j^o|j = 1, \cdots ,{k}^o\} $, precision and recall were used to evaluate performances of nonoverlapping cluster results. Precision represents the number of correctly classified cells over the volume of a result cluster (i.e., $P( {\Theta _i^{\prime},\Theta _j^o} ) = | {\Theta _j^o \cap \Theta _i^{\prime}} |/| {\Theta _i^{\prime}} |$), and recall is the number of correctly classified cells over the volume of ground truth (i.e., $R( {\Theta _i^{\prime},\Theta _j^o} ) = | {\Theta _j^o \cap \Theta _i^{\prime}} |/| {\Theta _j^o} |$) [36]. Their extensions to overlapping clusters have been proposed by El Ayeb et al. [36] as the inclusion rate and coverage rate, respectively .

Briefly, the IR evaluates the embeddedness of the result clusters to the ground-truth clusters. For each result cluster, $IR( {\Theta _i^{\prime}} ) = \mathop {max}\limits_j P( {\Theta _i^{\prime},\Theta _j^o} )$ defines the individual IR. Then, the overall IR is defined as the weighted sum of the individual IR, $IR( {\Psi ^{\prime}} ) = \mathop \sum \limits_i IR( {\Theta _i^{\prime}} )| {\Theta _i^{\prime}} |/\mathop \sum \limits_i | {\Theta _i^{\prime}} |$. On the other hand, the CR evaluates the embeddedness of the ground-truth clusters, and the individual CR is $CR( {\Theta _j^o} ) = \mathop {max}\limits_i R( {\Theta _i^{\prime},\Theta _j^o} )$. Then, the overall CR is $CR( {{\Psi }^o} ) = \mathop \sum \limits_j CR( {\Theta _j^o} )| {\Theta _j^o} |/\mathop \sum \limits_j | {\Theta _j^o} |$.

IR and CR were shown to be highly complementary, where IR is an indicator of how similar the result clusters are to the ground truth, and CR is an indicator of how well the ground-truth clusters are represented in the result clusters [36]. However, CR values are inflated when the clustering results are undersegmented, and IR values are inflated when the clustering results are oversegmented. To this end, we devised an cluster accuracy measure to handle overlapping clusters. For each result cluster and ground-truth cluster, we calculated the ratio between their intersection and union, known as the JI, as $JI( {\Theta _i^{\prime},\Theta _j^o} ) = | {\Theta _i^{\prime} \cap \Theta _j^o} |/| {\Theta _i^{\prime} \cup \Theta _j^o} |$. JI yields $JI( {\Theta _i^{\prime},\Theta _j^o} )$ = 1 if $\Theta _i^{\prime} = \Theta _j^o$ and $JI( {\Theta _i^{\prime},\Theta _j^o} ) = 0$ if there is no overlap. In analogy with CR, for each ground-truth cluster, we then defined the individual DA as the ideal overlap with the clustering results, $DA( {\Theta _j^o} ) = \mathop {max}\limits_i JI( {\Theta _i^{\prime},\Theta _j^o} )$. Then, the overall DA is $DA( {{\Psi }^o} ) = \mathop \sum \limits_j DA( {\Theta _j^o} )| {\Theta _j^o} |/\mathop \sum \limits_j | {\Theta _j^o} |$. We used IR, CR, and DA jointly to evaluate the concordance between the clustering results and ground-truth clusters.

**Calculating cophenetic correlations between the MSC cluster and ground-truth hierarchies**: We wanted to evaluate the overall concordance between the cluster hierarchy from MSC and the ground-truth hierarchy. We utilized the cophenetic distance to calculate pairwise distances among the elements where the cluster compactness served as the distance metric in the cluster hierarchy dendrogram. Likewise, the cophenetic distances among the ground-truth clusters were calculated using the correlation distance, $d = \sqrt {2( {1 - \rho } )} $. The cophenetic correlations between the cluster and ground-truth hierarchies were then calculated by Spearman’s correlations between the 2 distance matrices.

**Checking ground-truth cluster detections at different hierarchy layers**: To study the impacts of noise in detecting clusters at different hierarchical layers, we evaluated the overlaps between the inferred clusters and the ground-truth clusters at *L_in_* and *L_out_* by the Jaccard index. The Jaccard index measures the proportion of intersection between 2 sets, A and B, to its respective union by J(A, B) = |A ∩ B|/|A ∪ B|, and this can serve to measure how identical 2 clusters are. We applied J > 0.8 to identify ground-truth clusters captured in the inferred clusters. In addition, we explored the clusterability of inferred clusters, a statistical measure of significant clustering structure in a group of cells, by utilizing the Phiclust framework [29]. We expected that ground-truth clusters in *L_out_* should be further clusterable and imposed the Phiclust score, $\phi $> 0.9, as the recommended threshold by Phiclust. For ground-truth clusters in *L_in_*, we expected that they should not be further clusterable and imposed $\phi $< 0.8 as the recommended threshold by Phiclust [29]. In summary, ground-truth clusters in *L_out_* were deemed as detected in inferred clusters with J > 0.8 and $\phi $> 0.9, and ground-truth clusters in *L_in_* were deemed as detected with J > 0.8 and $\phi $< 0.8.

### Data processing for single-cell transcriptomes of gold-standard data, Lee *et al*. [1] (influenza/COVID-19–infected PBMCs) and PBMC 8k data

We performed rigorous data preprocessing and quality controls on scRNA-seq using the Seurat workflow [8]. First, we removed low-quality cells with mitochondrial reads >20%, median absolute deviation (MAD) >3, and average count >0. The doublets were identified by DoubletFinder [63] and removed. The dropout reads were inferred using adaptively thresholded low-rank approximation (ALRA) [64]. The filtered data were normalized and log-transformed by SCTransform [65]. Where applicable, we integrated the single-cell transcriptome across different conditions, individuals, or batches by canonical correlation analysis (CCA) [66].

Then, we selected highly variable genes as the features for cell clustering by calculating gene dispersions. Using the *modelGeneVar()* function from the scran package [9], we calculated biological variances of individual gene expressions from the log-normalized, preprocessed data by modeling the mean-variance curve as the technical variance [67]. We selected genes with biological variance *P* < 0.05 as the variable features for cell clustering. Pearson’s correlation across the selected features was used to calculate the cell similarity and perform MSC. The top 20 principal components (PCs) from the selected features were used to calculate the Euclidean distances.

Cell-type identification in PBMC 8k: The cell types were annotated by applying *SingleR* (v2.2.0) [68] with bulk RNA-seq of sorted immune cell populations, also known as the Monaco collection (GSE107011), as the reference transcriptome [69]. The Monaco collection data was provided through the *celldex* R package (v1.6.0) [68] and accessed through the *MonacoImmuneData()* function.

Cell-type identification in  Lee *et al.* [1]: Similar to the 8k PBMC dataset, most of the major cell types were annotated by *SingleR* (v2.2.0) by using the Monaco collection as the reference through the *MonacoImmuneData()* function in the *celldex* R package (v1.6.0). However, the Monaco collection included immune cells, but it erroneously annotated many cells as progenitors, which are expected to be present at a rate of 1–2% in PBMCs under normal circumstances, and did not detect platelets and red blood cells, as reported in Lee *et al*. [1] (Supplementary Fig. S10). To this end, we utilized the human primary cell atlas (HPCA) [70], a microarray collection of broader blood cell types, as the reference to supplement the cell-type annotations (Fig. 6B). Similar to the Monaco collection, the HPCA was accessed through the *HumanPrimaryCellAtlasData()* function in the *celldex* R package.

### Data processing and analysis for Wu *et al*. [39] breast cancer single-cell transcriptome atlas

Wu *et al*. [39] data included over 90,000 cells, and the several steps in data preprocessing applied in the gold-standard and Lee *et al*. [1] datasets were computationally prohibitive. These include dropout read imputations by ALRA, generation of integrated and normalized gene expression data by CCA, and calculation of cell similarity by Pearson’s correlation across the selected features. To this end, we performed a separate data preprocessing using a computationally efficient reciprocal PCA (RPCA) framework in the Seurat v5 workflow [8], and the Euclidean distances in the RPCA-based reduced dimension (top 50 PCs) were used to perform MSC. Specifically, we performed:

Data processing and marker analysis: The raw count matrices of single-cell transcriptomes across 20 samples from Wu *et al*. [39] were downloaded from the Broad Single-Cell Portal (https://singlecell.broadinstitute.org/single_cell/study/SCP1039). We removed low-quality cells with mitochondrial reads >20%, MAD >3, and average count >0. The doublets were identified by DoubletFinder and removed [63]. Considering the large number of cells (~100,000 cells) and samples to perform the integration of samplewise single-cell transcriptomes, we utilized a fast implementation of CCA, RPCA, in the Seurat v5 workflow (v5.1) in R (v4.2.0) to integrate the top 50 PCs across different samples to embed them into a common reduced dimension. UMAP embeddings were subsequently calculated from the RPCA integrated coordinates for further analysis. In tandem, we normalized the samplewise single-cell transcriptomes by the SCTransformation approach using “*SCTransform()*” in Seurat v5, and the normalized expressions were recorrected by synchronizing the median UMI across different samples by “*PrepSCTFindMarkers()*” in the Seurat v5 workflow. The recorrected data were utilized for calculating cluster markers by adopting the MAST framework [71] in “FindMarkers().” Ribosomal and mitochondrial rates and cellwise UMI counts served as the latent variables, and markers were identified by FDR < 0.05, requiring a greater proportion of cells in a cell cluster/group of interest to express a marker gene than the control cell groups.

M138-specific marker identification: We first compared M138 with the rest of the ECs using “*FindMarkers()*” with the MAST framework, as implemented in the Seurat v5 workflow. We applied FDR < 0.05 and required the marker genes to be expressed in at least 10% of cells in M138 and in less than 5% of the rest of ECs. We then checked if M138-specific markers within ECs were also endothelial markers by comparing their expressions in other major cell types. Similarly, we required the marker genes to be expressed in at least 10% of ECs and in less than 5% of the rest of cells.

Enrichment analysis of the M138-specific program in bulk samples with a good prognosis: We downloaded the raw count matrix for 1,080 primary tumor samples of breast cancers from The Cancer Genome Atlas (TCGA) RNA sequencing experiments [72] and performed counts per million normalization, followed by trimmed mean of M-values scaling [73] and log_2_(x + 1) transformation, using the edgeR R package (v3.38.1). We then adjusted for the batch variables (data generating center, date, and machine as identified in TCGA barcode) and patients’ age by the generalized linear model (*glm()* in the **stats** R package, v4.2.0). Similarly, we downloaded the log-normalized gene expression data of 1,974 samples from the METABRIC cohort [44] and adjusted for batch and age by the generalized linear model. Then, we utilized immunohistochemistry status for estrogen, progesterone, and Her2 where available and labeled ER^+^, Her2^+^, and ER^+^/Her2^+^ (double-positive) and TNBC (defined as ER^−^, PR^−^, and Her2^−^).

For each subtype and all breast cancer samples, we calculated the relative enrichments of M138-specific markers in individual bulk samples by the gene set variation analysis (GSVA) [43] R package (v1.44.1) implemented in R (v4.2.0). We calculated ssGSEA scores by the “*gsva()*” function in the GSVA R package with the method=“ssgsea” parameter and used the ssGSEA scores as a proxy for the presence of the capillary ECs captured by M138 in the bulk samples (Fig. 7E, D).

## Availability of Source Code and Requirements

Project name: Single-cell multi-scale clustering

Project homepage: https://github.com/songlabcodes/MSC

Operating system(s): Platform independent

Programming language: R

Other requirements: R 4.2.0 or higher.

License: Data files for examples are distributed under the CC0 1.0 Universal (CC0 1.0) Public Domain Dedication (https://creativecommons.org/publicdomain/zero/1.0/). Otherwise, the codes are distrubuted under the GPL-3.0 license as creative works.

Any restrictions to use by nonacademics: None.

RRID:SCR_027342

bio.tools ID: single-cell_multi-scale_clustering_msc

## Abbreviations

ALRA: adaptively thresholded low-rank approximation; ARI: Adjusted Rand Index; CCA: canonical correlation analysis; CR: coverage rate; CSN: cell–cell similarity network; DA: detection accuracy; EC: endothelial cell; eNN: embedded nearest neighbor; FACS: fluorescence-activated cell sorting; FDR: false discovery rate; FET: Fisher’s exact test; GSVA: gene set variation analysis; HPCA: human primary cell atlas; IR: inclusion rate; JI: Jaccard index; kNN: k-nearest neighbor; LEN: locally embedded network; MAD: median absolute deviation; METABRIC: Molecular Taxonomy of Breast Cancer International Consortium; MSC: multiscale clustering; NMI: Normalized Mutual Information; NN: nearest neighbor; PBMC: peripheral blood mononuclear cell; PC: principal component; PCA: principal component analysis; PMFG: planar maximally filtered graph; QC: quality control; RB modularity: Reichardt–Bornholdt modularity; RPCA: reciprocal principal component analysis; scRNA-seq: single-cell RNA sequencing; SNN: shared nearest neighbor; ssGSEA: single-sample gene set enrichment analysis; TCGA: The Cancer Genome Atlas; TNBC: triple-negative breast cancer; tSNE: t-distributed stochastic neighbor embedding; UMAP: Uniform Manifold Approximation and Projection; UMI: unique molecular identifier.

## Supplementary Material

giaf111_Supplemental_Files

giaf111_Authors_Response_To_Reviewer_Comments

giaf111_Authors_Response_To_Reviewer_Comments_Original_Submission

giaf111_GIGA-D-25-00020_Original_Submission

giaf111_GIGA-D-25-00020_Revision_1

giaf111_GIGA-D-25-00020_Revision_2

giaf111_Reviewer_1_Report_Original_Submission Qianqian Song -- 2/8/2025

giaf111_Reviewer_1_Report_Revision_1 Qianqian Song -- 8/4/2025

giaf111_Reviewer_2_Report_Original_Submission Qi Liu, Ph.D. -- 2/8/2025

giaf111_Reviewer_2_Report_Revision_1 Qi Liu, Ph.D. -- 8/10/2025

## Data Availability

All of the raw and processed single-cell and bulk RNA sequencing data utilized in this study are available on Synapse with Synapse project ID, the project Synapse ID, syn52966803 [74]. Each folder under the project is assigned a unique Synapse ID as follows. **10×8k PBMC benchmark data**: The raw and processed count matrix is available on Synapse under synapse IDs syn52967814 (raw matrix) and syn53009488 (processed Seurat and SingleCellExperiment objects). **scRNA-seq of PBMCs from influenza, COVID-19–infected, and healthy control samples from Lee et al. 2020**: The data underlying this study are available in Gene Expression Omnibus (GEO) [75] and can be accessed with accession number GSE149689. The processed data are available under Synapse ID, syn53058712. **scRNA-seq of breast cancer single-cell atlas from Wu et al. 2021**: The raw count matrix and cell-level metadata were downloaded from the Broad Single-Cell Portal [76] under the study ID, SCP1039. The processed data are available on Synapse under Synapse ID, syn63695719. **Breast cancer bulk transcriptome data from TCGA and METABRIC**: The raw count matrix and the preprocessed, log-normalized data of TCGA breast cancer RNA sequencing data are available under Synapse ID, syn64621142. The preprocessed METABRIC data are also available under Synapse ID, syn64621177. **Code availability:** The R codes and MSC R package underlying this article are available in Zenodo [77]. A snapshot of our GitHub project is archived in Software Heritage [78], and the workflow is also available in Workflow hub [79]. The developmental version of MSC is available on GitHub [80].

## Additional Files

**Supplementary Fig. S1**. Proportions of detected clusters by MSC^COR^ and MSC^EUC^ at different ground-truth layers under different Δρ = 0.125 and 0.25 (labeled at the top) and cluster regularities (labeled on the right). X-axis: Noise amplitudes by σ. Y-axis: %. identified ground-truth cluster within L_in_ (upper) or L_out_ (lower).

**Supplementary Fig. S2**. tSNE plots of the PBMC 8k dataset. (A) The major immune cell types (top) and subtypes (bottom) are annotated into different colors with respective labels. (B) The clustering results from various methods: AdaptSplit results from Pearson’s correlations and Euclidean distances are shown along with other benchmark methods.

**Supplementary Fig. S3**. Detection accuracy of immune cell types and subtypes by different clustering methods. Clustering methods are labeled by different colors shown on the bottom right legend. (A) Tree map of the immune cell types and subtypes present in the PBMC 8k data. (B, C) Detection accuracy of the immune cell types and subtypes at different stages in A (B) and by different major cell types (C) by different clustering methods.

**Supplementary Fig. S4**. Compactness differentiates parent and child clusters with distinctions in Phiclust score as a statistical measure of clusterability. Each window represents different noise levels, and each red/blue dot represents a ground-truth child/parent cluster detected in the respective MSC^COR^ results. X-axis: Compactness, Y-axis: Phiclust score.

**Supplementary Fig. S5**. Evaluation of compactness as a function of the exponent α, υ(α), by different hierarchical structures and similarity measures. (A, B) Compactness for ground-truth parent clusters (green) and child clusters (red) in LENs computed from Pearson’s correlation (A) and Euclidean distance (B) on simulated data with hierarchy among irregular-sized clusters across various α in [0,3] across 10 random replicates. The horizontal dotted lines show the transition points, α′, where υ_parent_(α′) = υ_child_(α′). On the far right, the boxplot of α′ values is shown. (C, D) Similar plots as A for LENs computed from Pearson’s correlation (C) and Euclidean distance (D) on simulated data with hierarchy among regular-sized clusters.

**Supplementary Fig. S6**. Sparsity of LENs and SNNs for different gold-standard scRNA-seq datasets. Sparsities (*C_s_*) of LENs constructed from Pearson’s correlations (LEN:Correlation), Euclidean distance (LEN:Euclidean), and SNNs are shown.

**Supplementary Fig. S7**. Computational complexity analysis for different clustering methods. Different methods are labeled in different colors, and different single-cell data are labeled as different shapes, as shown in the bottom legend. (A) Plot of runtime for different clustering methods (y-axis) against single-cell transcriptome datasets with varying numbers of cells (x-axis). The axes are in log_10_ scales. The scaling exponents (η) for the runtimes at different numbers of cells are labeled for each method. (B) Plot of memory (y-axis) against single-cell transcriptome datasets with varying numbers of cells (x-axis).

**Supplementary Fig. S8**. Scatterplot of pairwise Pearson’s correlation (ρ) against the proportion of commonly expressed genes in the respective cell pairs in LEN for PBMC 8k.

**Supplementary Fig. S9**. Scatterplot to calculate the compactness scaling parameter (α) for the PBMC 8k dataset. X-axis: Module sizes randomly sampled from selecting random nodes and traversing 2 links to identify closely connected nodes. Y-axis: Scaling parameters with $\upsilon ( {{\alpha }_o} ) = 1$.

**Supplementary Fig. S10**. Inferred cell types of Lee dataset by SingleR with the Monaco collection as the reference set.

**Supplementary Fig. S11**. Number of detected immune subsets by different methods (y-axis) and detection accuracy thresholds (x-axis) for the Lee dataset.

**Supplementary Fig. S12**. UMAP plots showing marker expressions for platelet subpopulations identified by MSC. Respective gene names are shown on top of each panel, and the child clusters of the major platelet cluster M16 in Fig. 5A are marked.

**Supplementary Fig. S13**. UMAP plots show M138-specific marker expressions in endothelial cells.

**Supplementary Fig. S14**. Kaplan–Meier plots show prognostic significance of stratifying patients with breast cancer by median expressions of M138-specific markers in predicting relapse-free survival across the bulk transcriptome of 7,830 samples from 55 independent studies [45]. Four markers (CA4 [also known as RP17], ATOH8, TIMP4, and TNMD) out of the 6 tested genes with significant stratification by logrank *P* < 0.05 are shown.

**Supplementary Data S1**. Metadata for individual cells from the Lee et al. 2020 dataset. It includes inferred cell types in column “inferred.cell.type.broad,” for major cell types from PBMCs, and more specific subtypes in “inferred.cell.type.fine.” (B) Multiscale clusters identified MSC in .GMT format. (C) Table of MSC identified clusters. For each cluster in each row, it specifies its parent cluster, cluster compactness, and size. D. Enrichments of individual samples in MSC clusters by Fisher’s exact test (FET).

**Supplementary Data S2**. (A) Metadata for single-cell transcriptome of breast cancers from Wu et al. 2021. (B) Clustering results from SNN-based Louvain clustering at γ = 0.4, 0.8, and 1.2. (C) Multiscale clusters identified MSC in .GMT format. (D) Table of MSC identified clusters. For each cluster in each row, it specifies its parent cluster, cluster compactness, and size. (E) Jaccard index between MSC clusters and best-mapped cell types, minor cell types, and subsets by supervised subclustering in Wu et al. 2021. (F) Jaccard index between MSC clusters and best-mapped SNN-based Louvain clusters at different resolutions. (G) Differential expression statistics of M138-specific markers. Only includes a list of significant markers genes (FDR < 0.05, fold change > 1) for M138 within endothelial cells. (H) Clinical metadata for TCGA breast cancer cohort. Last columns include ssGSEA scores within each subtype and all primary tumor samples. (I) Clinical metadata for METABRIC breast cancer cohort. The last columns include ssGSEA scores within each subtype and all primary tumor samples.
