# Supplementary material for: Unsupervised multiscale clustering of single-cell transcriptomes to identify hierarchical structures of cell subtypes
Source: Gigascience. 2025 Oct 9;14:giaf111. doi: 10.1093/gigascience/giaf111 (PMC12509883; doi:10.1093/gigascience/giaf111)
Supplement: giaf111_Supplemental_Files [file giaf111_supplemental_files.zip › SUPPLEMENTARY MATERIAL.pdf]

SUPPLEMENTAL RESULTS

Detection of immune cell types and subtypes in PBMC scRNA-seq

We processed and analyzed scRNA-seq of 8,381 peripheral blood mononuclear cells (PBMC) from a healthy donor from 10x website, and performed and the benchmark clustering methods (see **METHODS** for data processing details) to identify the cell clusters. The cell types were annotated by *SingleR* (v2.2.0)[1] with bulk RNA-seq of sorted immune cell populations, also known as the Monaco collection (GSE107011)[2], as the reference transcriptome. This identified various immune subtypes (B-cell, CD4/CD8 T-cells, NK cells, monocytes, dendritic cells) and progenitor cells in the data (**Supplemental Figure 2A**).

Using the annotated cell types as a silver standard ground-truth clusters, we evaluated the performances of the various clustering methods to detect these immune cell types, and observed distinct differences in their clustering results. We observed that AdaptSplit results from Pearson's correlation (MSC_{1L}^{COR}) and Euclidean distance in the PC space (MSC_{1L}^{EUC}) identified coarse-grained clusters that mostly aligned with the major immune cell types, compared to the other benchmark results (**Supplemental Figure 2B**). For example, B-cells and NK-cells were correctly identified as single clusters. Notably, MSC_{1L}^{COR} correctly identified the myeloid cells into a single cluster, in contrast to the Euclidean distance-based results that differentiated several myeloid subpopulations such as dendritic cells and classical/intermediate monocytes. On the other hand, the clustering results from SNN- or aKNNO-based Louvain clustering in different resolutions and SC3 tended to over-split the clusters and NK-cells were the only major cell type identified as a

single-cluster. In contrast, the clusters by CIDR tend to under-split the clusters and failed to discriminate NK-cells from T-cells.

Based on the inferred cell types, we systematically evaluated how different clustering methods accurately detected immune cell hierarchy present in this data by capturing clusters at different hierarchical levels (**Supplemental Figure 3A**). At each hierarchy level, we calculated how similar each cluster from each method to the defined cell (sub)types by Jaccard Index. We observed that the multi-scale clustering results, $MSC_{ML}^{COR}$and MSC_{ML}^{EUC}, consistently captured the most similar clusters at all hierarchical levels and within different major immune types, compared to other methods (**Supplemental Figure 3B, C**). In contrast, the SNN- and aKNNO-based clustering with various resolutions emphasized detection of cell subtypes at the third level and failed to realized more granular structures in spite of the varying resolutions.

Behavior of compactness exponent α on different clustering structures

Previously, we defined the cluster compactness measure, $\upsilon(\alpha)=\overline{SPD}/\log(N_c)^{\alpha}$, where $\overline{SPD}$ is the average of shortest path distances of all cell pairs in a network, α is the compactness scaling parameter, and N_c is the number of nodes in cluster c[3]. This was inspired by the formal scaling relations between $\overline{SPD}$ and N_c, $\log(\overline{SPD}) \sim \log(N_c)^{\kappa}$, to define the small-worldness of networks where $\kappa \approx 1$ as the classical small-world, $\kappa < 1$ as ultra-small world, and $\kappa > 1$ as semi-small world[4]. In this sense, α in υ(α), serves as the proxy for κ, and α’ values at which the parent and its child compactness coincides (i.e. $\upsilon_{parent}(\alpha') = \upsilon_{child}(\alpha')$) serve as the break points that, for $\alpha < \alpha'$, the parent clusters are deemed more compact than the child clusters and, for $\alpha > \alpha'$, the child clusters are more compact than the parent clusters[3]. This has

been effective in identifying multi-scale clusters from embedded networks in topological sphere[3], and has been instrumental in identifying meaningful multi-scale gene networks to elucidate *de novo* disease mechanisms and key regulators[5–9].

Herein, we investigated how α' behaves in simulated data sets with respect to different cluster hierarchies and similarity measures (**Supplemental Figure 3**). To this end, we utilized the multivariate Gaussian generators with regularly (or irregularly) sized clusters nested in parental clusters to simulate data with hierarchical structures (see **Methods**), and generated 10 replicates per case. We computed the LENs from the simulated data, and calculated the compactness of the nested and parent ground-truth clusters to observe how the hierarchical structures in the data impact the compactness. Firstly, we observed that the compactness behaved robustly with respect to different similarity measures by exhibiting similar α' for irregularly sized clusters (**Supplemental Figure 3 A, B**) and regularly sized clusters (**Supplemental Figure 3C, D**). On the other hand, the irregularly sized clusters exhibited slightly lower α' (~ 2.6; **Supplemental Figure 3A, B**) than the regularly sized clusters (~ 2.8; **Supplemental Figure 3C, D**). These indicate α' can distinguish the structural differences and υ(α) can exploit the parent-child cluster hierarchies through incorporating small-worldness through the scaling exponent, α.

Computational Complexity Analysis

We analyzed the required computational resources to perform MSC and other benchmark methods (CIDR, SC3 and SNN at multiple resolutions (γ=0.4, 0.8 and 1.2) for Louvain clustering) on different single-cell transcriptome data with varying numbers of cells.

We curated a set of publicly available scRNA-seq data whose sizes vary from small sized cohorts (< 10,000 cells) to atlas-sized cohorts (> 100,000 cells) to evaluate how the required computational resources scale with data sizes. These include PBMC of healthy donors from 10x Genomics (PBMC 3k: 2,700 cells, PBMC 8k: 8,364 cells), melanoma tumors (Jerby Arnon *et al.* 2018: 7,186 cells from metastatic melanoma)(1), PBMCs of influenza, COVID-19 infected and healthy controls (Lee *et al.* 2020: 62,301 cells)(2), and breast tumors (Wu *et al.* 2021: 92,232 cells)(3) and human colon cancer atlas (HCCA; 333,212 cells)(4) (see **Methods** for data processing details).

While MSC is flexible to handle different similarity and dissimilarity measures, we selected the Euclidean distances by the principal components to objectively compare the runtimes and required memories for different clustering methods, especially for those dependent on Euclidean distances. We used the first 30 principal components (PCs) for the smaller data sets (PBMC 3k, PBMC 8k, Jerby Arnon *et al.* 2018 and Lee *et al.* 2020), and 50 PCs for the larger data sets (Wu *et al.* 2021, HCCA) to commensurate with the increasing number of cell populations for the larger data.

We tested different methods on high performance computing (HPC) units with 2.9 GHz Intel Cascade Lake 8268 processors at Icahn school of medicine at Mount Sinai (ISMMS). We utilized parallel computations with 8 cores for methods with available parallel functionalities (SC3 and MSC), and assigned 8GB of memory per each core. These numbers were deliberately selected to simulate the available computing resources for personal computers (PCs) or laptops supporting multi-core computing.

The runtimes differed greatly amongst the clustering methods (**Supplemental Figure 7A**). For the smaller data sets with sizes < 10,000 cells, SNN-based clustering at multiple resolutions showed the shortest runtimes (< 100s) across the data sets while MSC, CIDR and SC3 showed comparable runtimes (< 3,000s). For the larger data sets over 10,000 cells, SNN-based clustering still showed the best runtimes, followed by MSC. On the other hand, CIDR and SC3 could not be performed due to excessive memory usages (> 128GB) for data sets > 10,000 cells while MSC and SNN based clusterings maintained tractable memory usages under 50GB (**Supplemental Figure 7B**) .

The longer MSC runtimes compared to the SNN-based clustering are expected due to several additional steps in MSC to explore the cell hierarchies in an unsupervised manner. These additional steps include the unsupervised search for the granular clustering solutions within the pool of cells to realize the major cell types, followed iterative subclustering to search the meaningful cell subsets to eventually identify the data-driven cell hierarchy.

We summarized how the runtimes from different methods scales with the data size (n) through analyzing the overall computational complexity, $O(n) \sim n^{\eta}$, where η is the scaling factor. MSC and SNN clusterings showed similar complexity of $\eta \sim 1.3$, while SC3 showed $\eta \sim 2$ and CIDR showed $\eta \sim 2.7$. These imply that, for doubling the number of cells in the single-cell data, MSC and SNN will be expected to increase the runtime by 2.46 times, SC3 runtime to increase by 4 times, and CIDR runtime to increase by 6.5 times.

Overall, MSC is a scalable clustering method to analyze from small to atlas-sized single-cell cohorts with feasible computational resources on personal machines.

With access to high performance computing, MSC can be further parallelized to improve the overall runtime.

SUPPLEMENTAL FIGURES

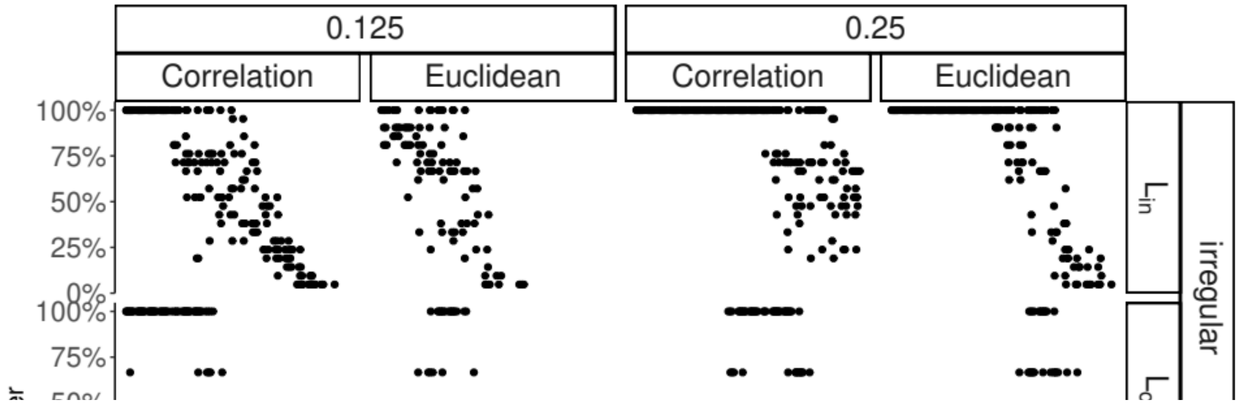

0.125
0.25
Correlation
Euclidean
Correlation
Euclidean
100%
75%
50%
25%
0%
100%
75%
L_{in}
irregular

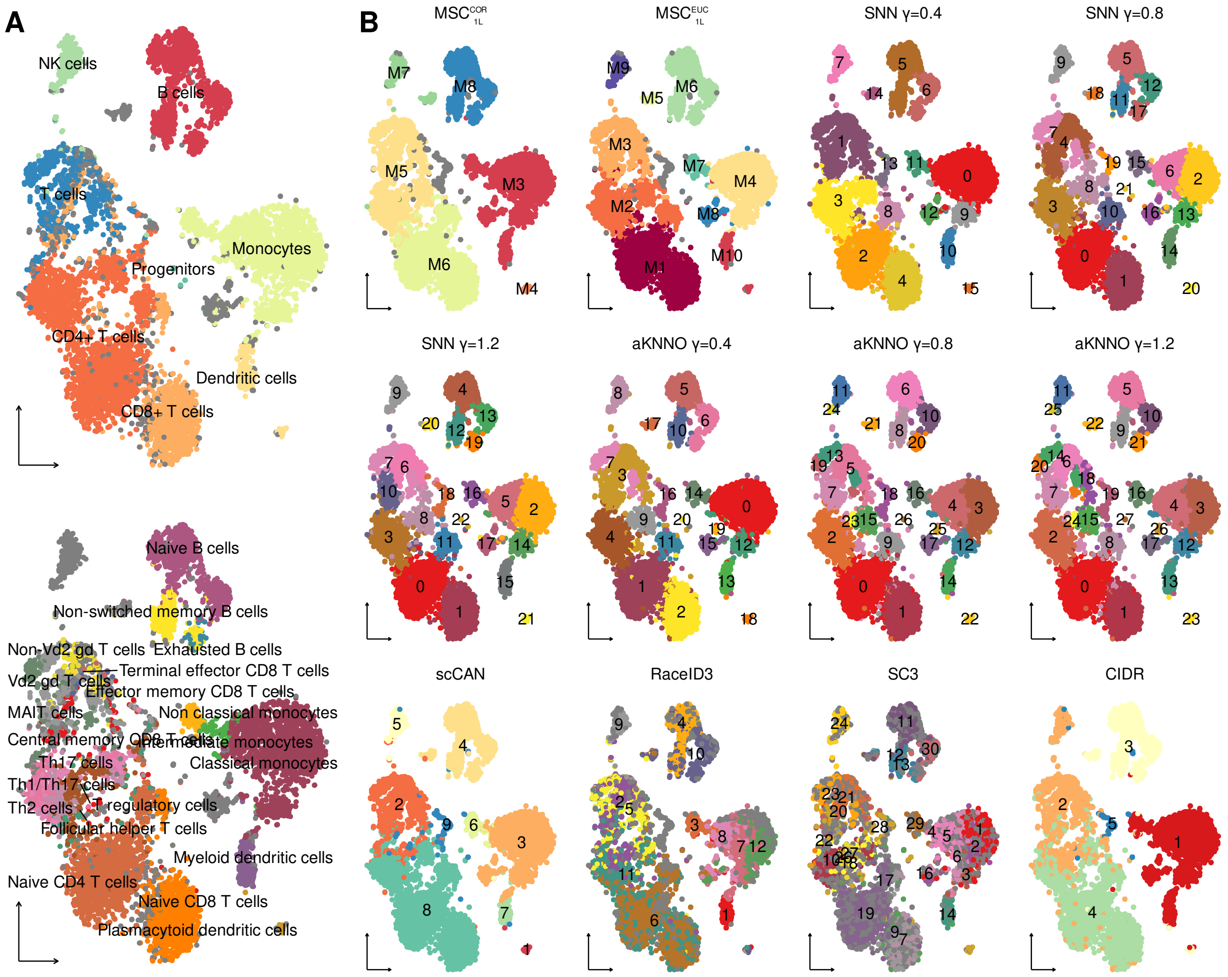

Supplemental Figure 2. tSNE plots of PBMC 8k data set. A. The major immune cell types (top) and subtypes (bottom) are annotated into different colors with respective labels. **B. The clustering results from various methods**: AdaptSplit results from Pearson's correlations (MSC_{1L}^{COR}) and Euclidean distances (MSC_{1L}^{EUC}) are shown along with other benchmark methods.

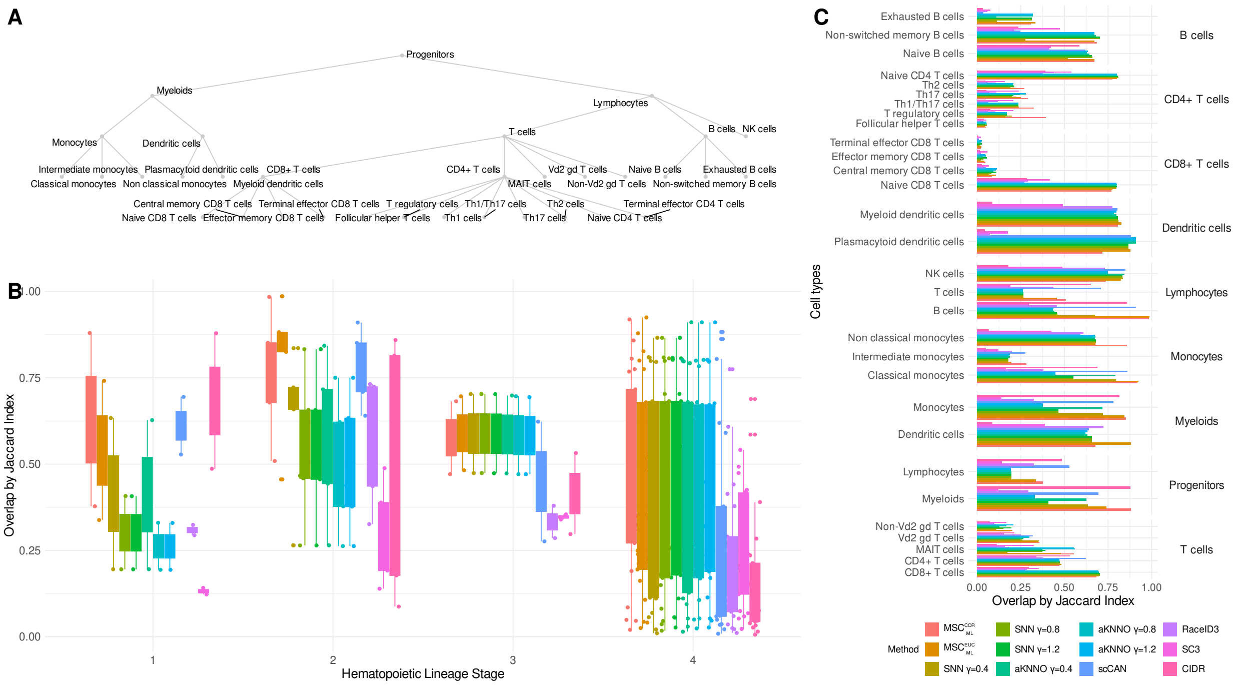

Supplemental Figure 3. Detection accuracy of immune cell types and subtypes by different clustering methods. Clustering methods are labeled by different colors shown on the bottom right legend. **A. Tree map of the immune cell types and subtypes present in PBMC 8k data. B, C. Detection accuracy of the immune cell types and subtypes** at different stages in A (**B**) and by different major cell types (**C**) by different clustering methods.

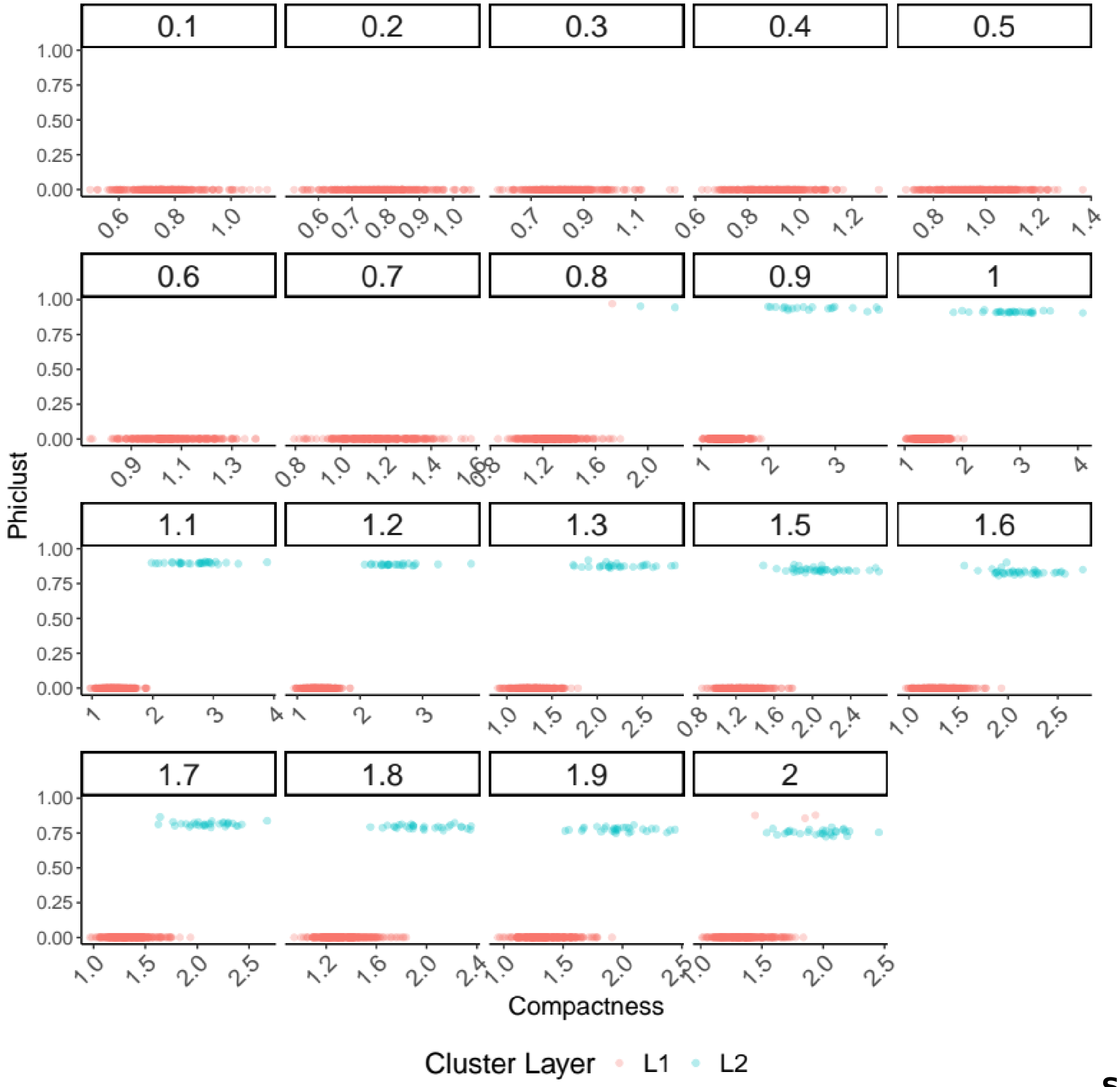

Supplemental Figure 4. Compactness differentiates parent and child clusters with distinctions in Phiclust score as a statistical measure of clusterability. Each window represents different noise levels, and each red/blue dot represents a ground-truth child/parent cluster detected in the respective MSC^{COR} results. X-axis: Compactness, Y-axis: Phiclust score.

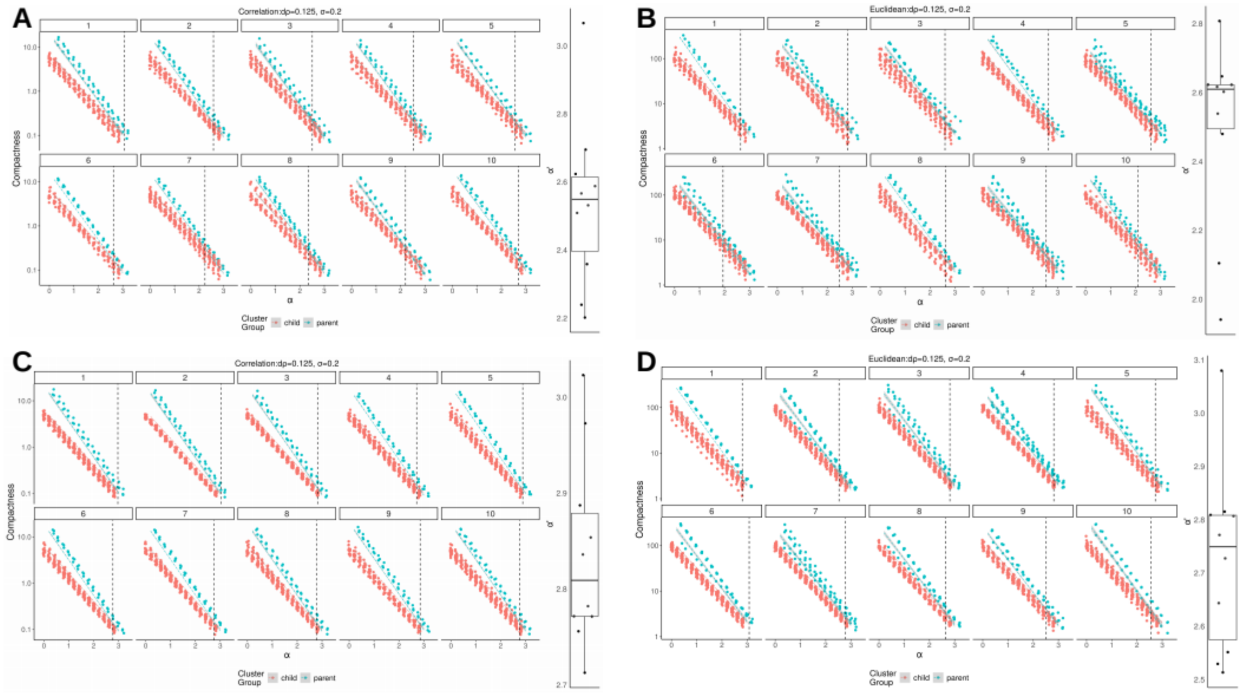

Supplemental Figure 5. Evaluation of compactness as a function of the exponent α, υ(α), by different hierarchical structures and similarity measures. A, B. Compactness for ground-truth parent clusters (green) and child clusters (red) in LENs computed from Pearson's correlation (**A**) and Euclidean distance (**B**) on simulated data with hierarchy among irregular sized clusters. across various α in [0,3] across 10 random replicates. The horizontal dotted lines show the transition points, α', where $\upsilon_{parent}(\alpha') = \upsilon_{child}(\alpha')$. On the far right, the boxplot of α' values is shown. **C, D**. Similar plots as **A** for LENs computed from Pearson's correlation (**C**) and Euclidean distance (**D**) on simulated data with hierarchy among regular sized clusters.

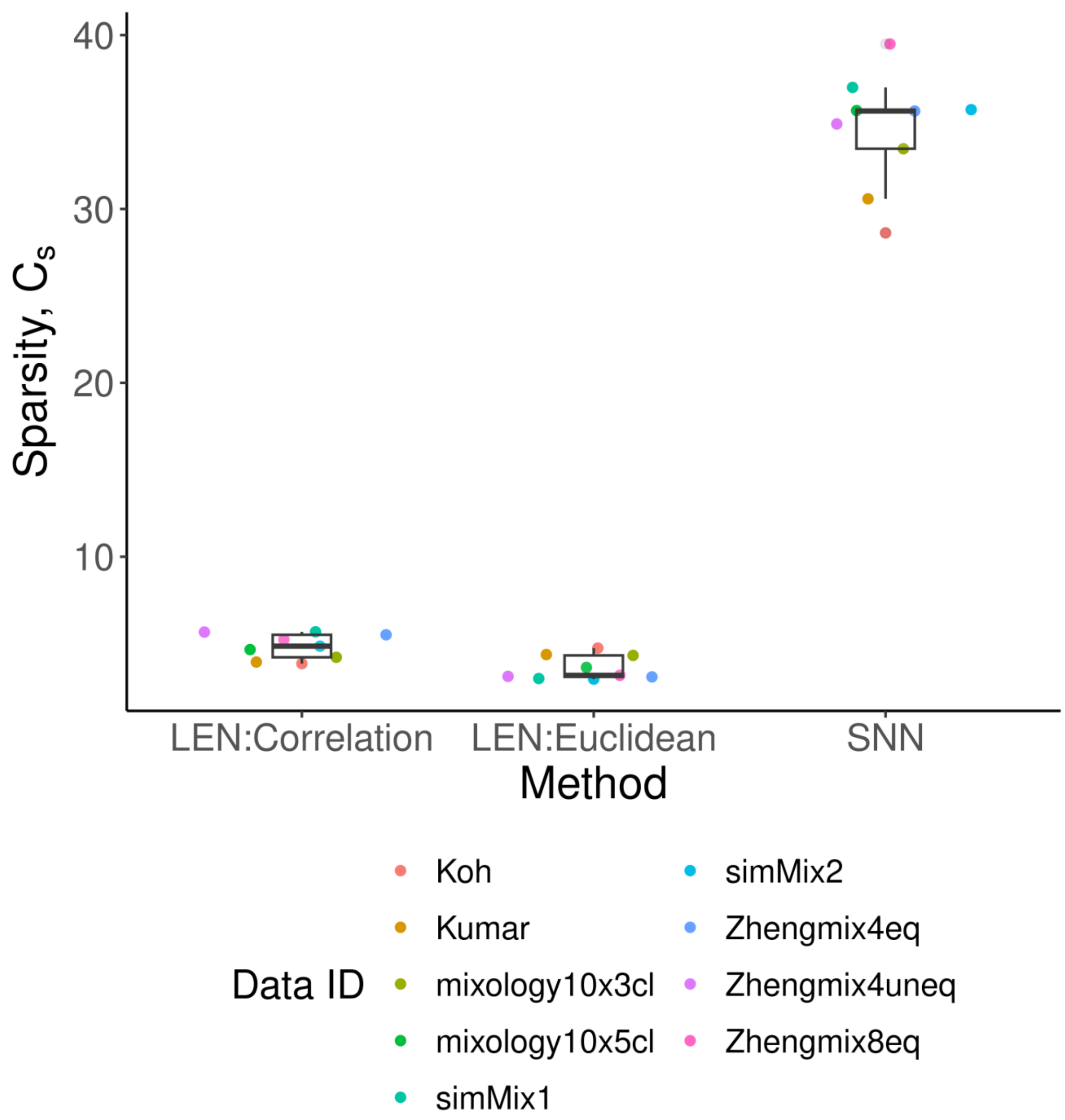

Supplemental Figure 6. Sparsity of LENs and SNNs for different gold standard scRNA-seq data sets. Sparsities (C_s) of LENs constructed from Pearson's correlations (LEN:Correlation), Euclidean distance (LEN:Euclidean) and SNNs are shown.

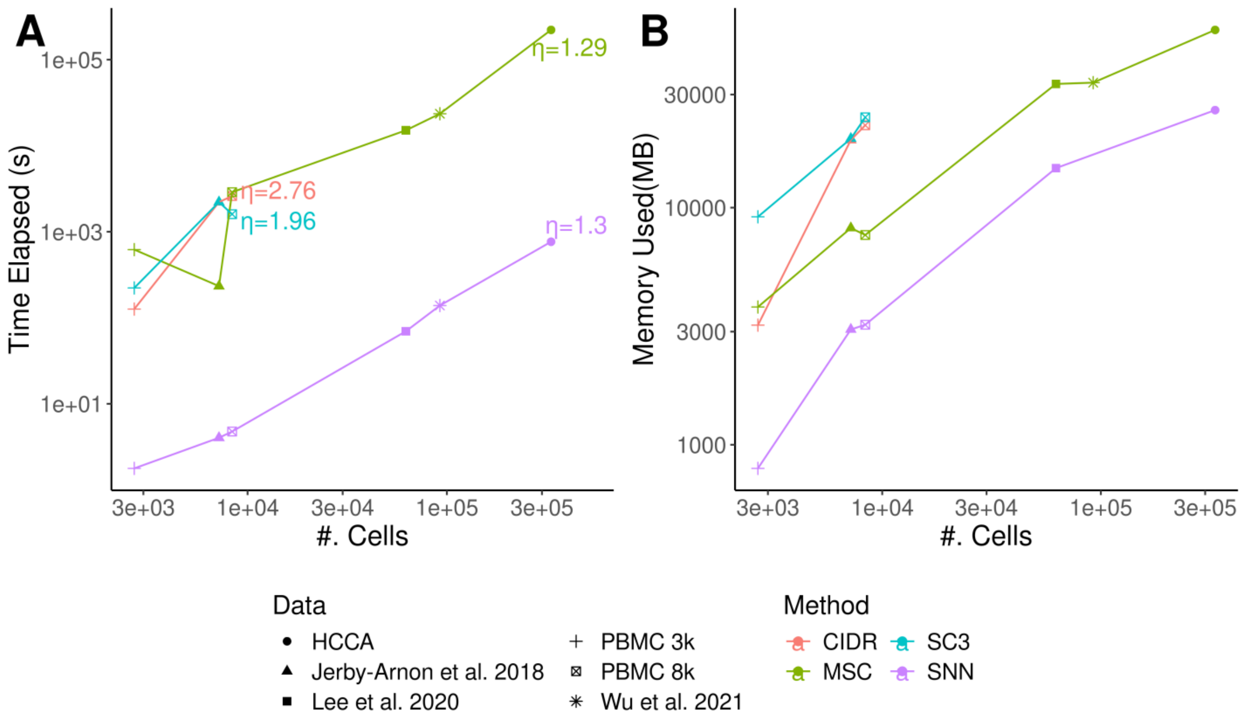

Supplemental Figure 7. Computational complexity analysis for different clustering methods. Different methods are labeled in different colors, and different single-cell data are labeled as different shapes as shown in the bottom legend. **A.** Plot of runtime for different clustering methods (y-axis) against single-cell transcriptome data sets with varying numbers of cells (x-axis). The axes are in log10 scales. The scaling exponents (η) for the runtimes at different numbers of cells are labeled for each method. **B**. Plot of memory (y-axis) against single-cell transcriptome data sets with varying numbers of cells (x-axis).

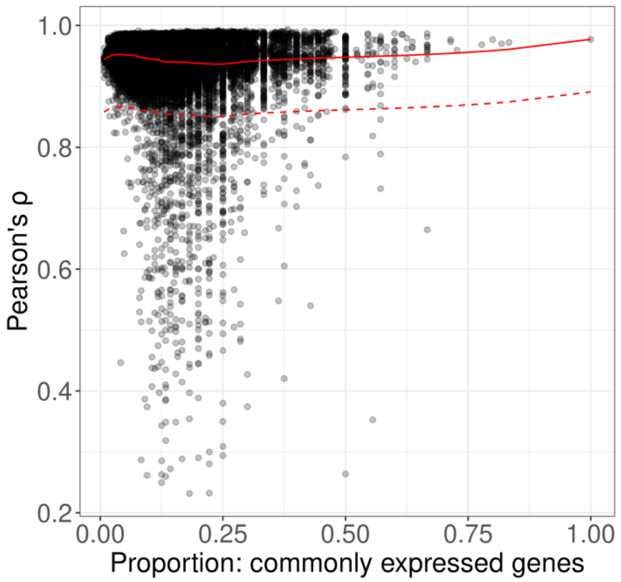

Supplemental Figure 8. Scatter plot of pairwise Pearson's correlation (ρ) against the proportion of commonly expressed genes in the respective cell pairs in LEN for PBMC 8k.

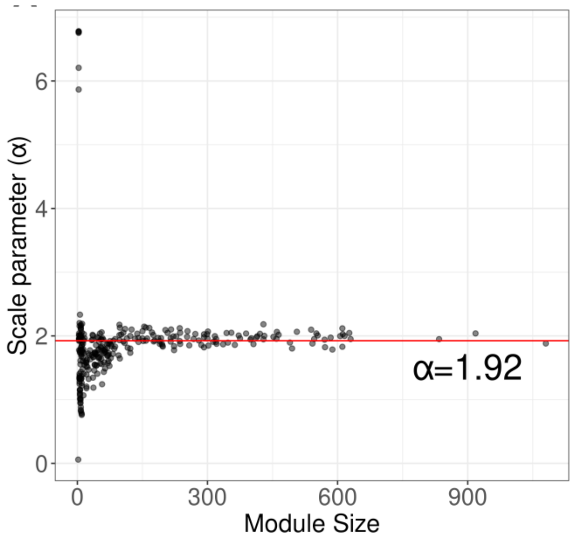

Supplemental Figure 9. Scatter plot to calculate the compactness scaling parameter (α) for PBMC 8k data set. X-axis: Module sizes randomly sampled from selecting random nodes and traversing two links to identify closely connected nodes. **Y-axis**: Scaling parameters with $\upsilon(\alpha_o)=1$.

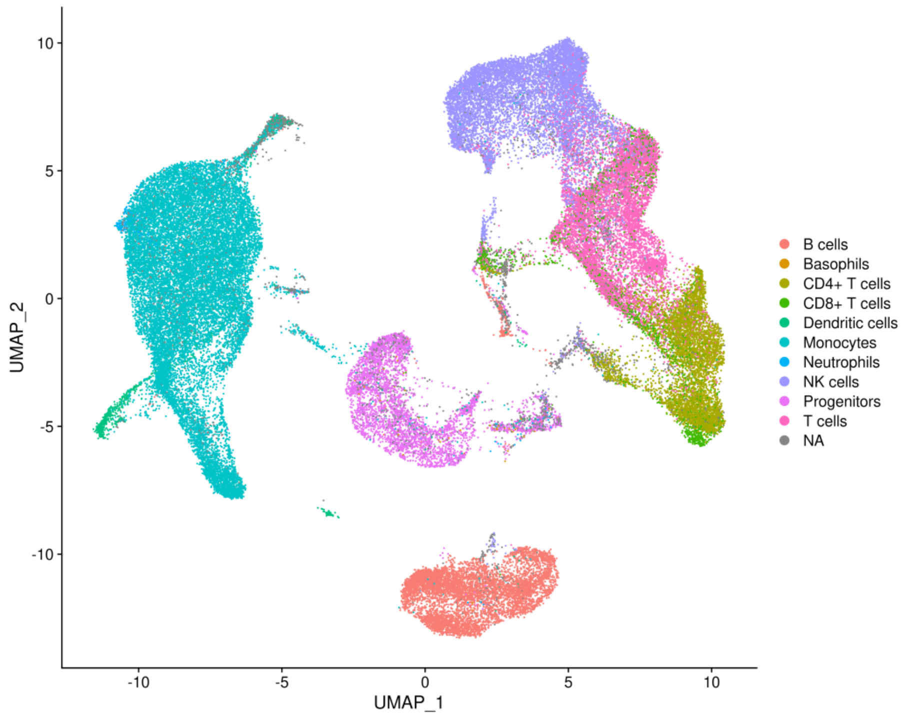

Supplemental Figure 10. Inferred cell types of Lee data set by SingleR with the Monaco collection as the reference set.

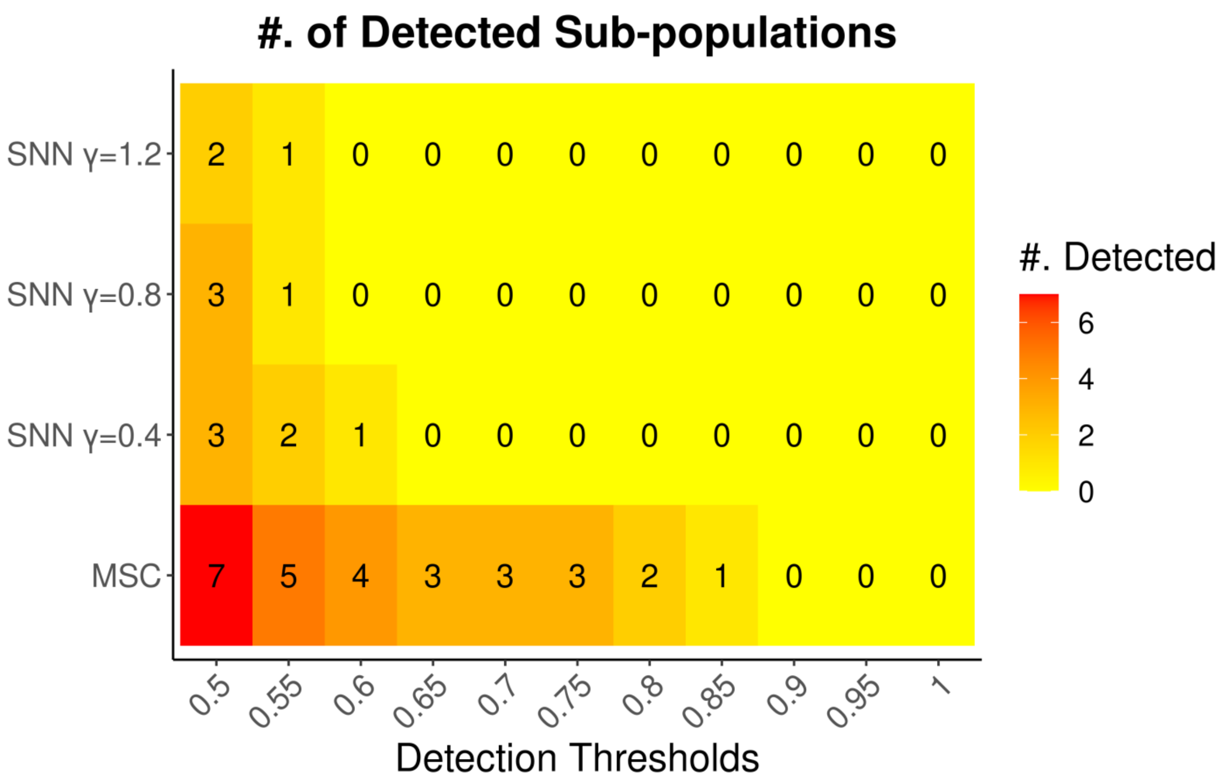

Supplemental Figure 11. Number of detected immune subsets by different methods (y-axis) and detection accuracy threaholds (x-axis) for Lee data set.

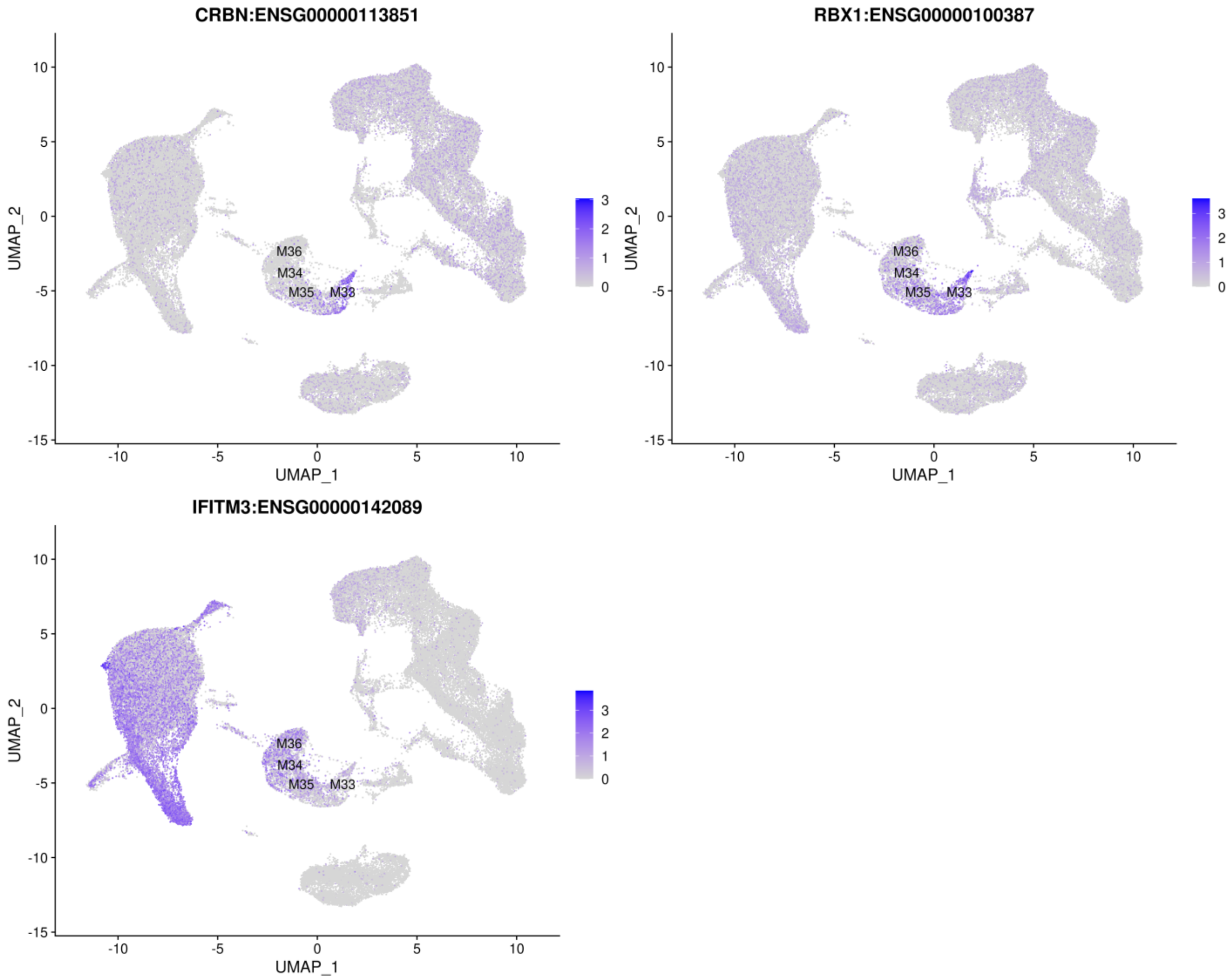

Supplemental Figure 12. UMAP plots showing marker expressions for platelet subpopulations identified by MSC. Respective gene names are shown on top of each panel, and the child clusters of the major platelet cluster M16 in **Figure 5A** are marked.

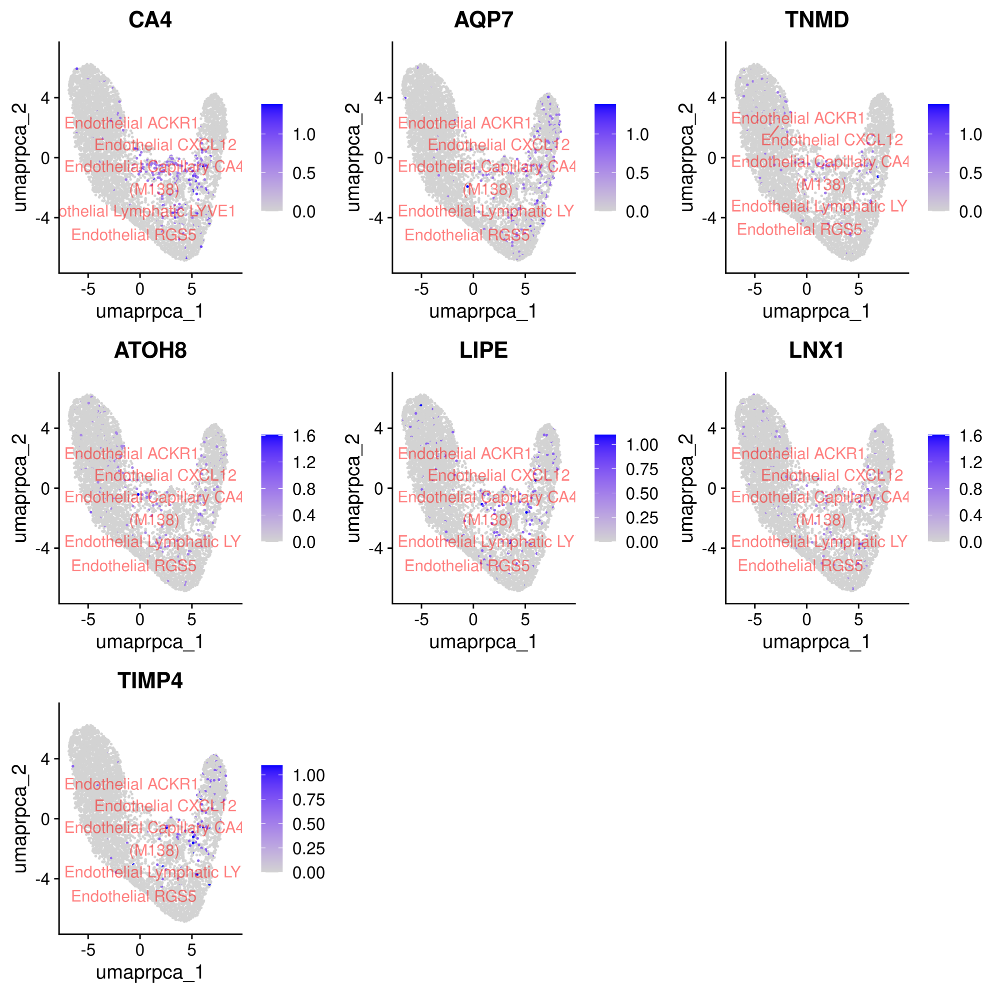

Supplemental Figure 13. UMAP plots to show M138-specific marker expressions in endothelial cells.

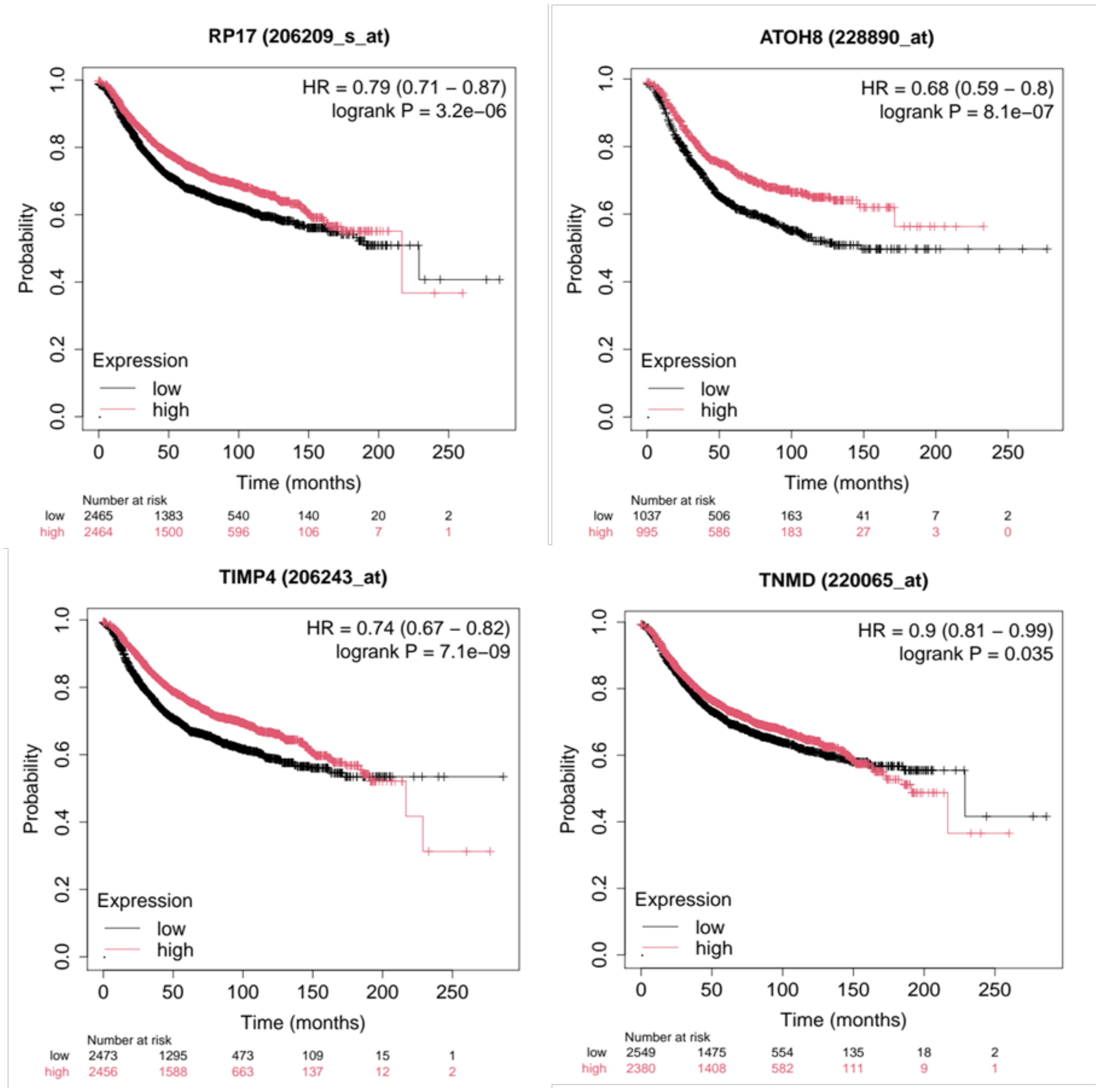

Supplemental Figure 14. Kaplan-Meier plots to show prognostic significance of stratifying breast cancer patients by median expressions of M138-specific markers in predicting relapse-free survival across bulk transcriptome of 7,830 samples from 55 independent studies(5). Four markers (CA4 (also known as RP17), ATOH8, TIMP4 and TNMD) out of the 6 tested genes with significant stratification by logrank p-value < 0.05 are shown.

SUPPLEMENTARY DATA

Supplementary Data 1. Meta data for individual cells from Lee *et al.* 2020 data set. It includes inferred cell types in column, "inferred.cell.type.broad", for major cell types from PBMC, and more specific subtypes in "inferred.cell.type.fine". **B**. Multi-scale clusters identified MSC in .GMT format. **C**. Table of MSC identified clusters. For each cluster in each row, it specifies its parent cluster, cluster compactness and size.

Supplementary Data 2. **A**. Meta data for single-cell transcriptome of breast cancers from Wu *et al.* 2021. **B**. Clustering results from SNN-based Louvain clustering at γ=0.4, 0.8 and 1.2. **C**. Multi-scale clusters identified MSC in .GMT format. **D**. Table of MSC identified clusters. For each cluster in each row, it specifies its parent cluster, cluster compactness and size. **E**. Jaccard index between MSC clusters and best mapped cell types, minor cell types and subsets by supervised subclustering in Wu *et al.* 2021. **F**. Jaccard index between MSC clusters and best mapped SNN-based Louvain clusters at different resolutions. **G**. Differential expression statistics of M138-specific markers. Only includes list of significant markers genes (FDR < 0.05, fold change > 1) for M138 within endothelial cells. **H**. Clinical meta data for TCGA breast cancer cohort. Last columns include ssGSEA scores within each subtype and all primary tumor samples. **I**. Clinical meta data for METABRIC breast cancer cohort. . Last columns include ssGSEA scores within each subtype and all primary tumor samples.

REFERENCES

1. Aran, D. *et al.* Reference-based analysis of lung single-cell sequencing reveals a transitional profibrotic macrophage. *Nat Immunol* **20**, 163–172 (2019).
2. Monaco, G. *et al.* RNA-Seq Signatures Normalized by mRNA Abundance Allow Absolute Deconvolution of Human Immune Cell Types. *Cell Rep* **26**, 1627-1640.e7 (2019).
3. Song, W.-M. & Zhang, B. Multiscale embedded gene co-expression network analysis. *PLoS computational biology* **11**, e1004574 (2015).
4. Song, W.-M., Di Matteo, T. & Aste, T. Building complex networks with Platonic solids. *Physical Review E* **85**, 046115 (2012).
5. Song, W.-M. *et al.* Multiscale network analysis reveals molecular mechanisms and key regulators of the tumor microenvironment in gastric cancer. *International journal of cancer* **146**, 1268–1280 (2020).

6. Song, W.-M. *et al.* Network models of primary melanoma microenvironments identify key melanoma regulators underlying prognosis. *Nature communications* **12**, 1–14 (2021).
7. Song, W. M. *et al.* Multiscale protein networks systematically identify aberrant protein interactions and oncogenic regulators in seven cancer types. *J Hematol Oncol* **16**, 120 (2023).
8. Wang, M. *et al.* Molecular networks and key regulators of the dysregulated neuronal system in Alzheimer's disease. *BioRxiv* 788323 (2019).
9. Wang, Q. *et al.* The landscape of multiscale transcriptomic networks and key regulators in Parkinson's disease. *Nature communications* **10**, 1–15 (2019).
