# Supplementary material for: Unsupervised multiscale clustering of single-cell transcriptomes to identify hierarchical structures of cell subtypes
Source: Gigascience. 2025 Oct 9;14:giaf111. doi: 10.1093/gigascience/giaf111 (PMC12509883; doi:10.1093/gigascience/giaf111)
Supplement: giaf111_GIGA-D-25-00020_Original_Submission [file giaf111_giga-d-25-00020_original_submission.pdf]

GigaScience

Unsupervised multi-scale clustering of single-cell transcriptomes to identify hierarchical structures of cell subtypes

--Manuscript Draft--

**Manuscript Number:**	GIGA-D-25-00020
**Full Title:**	Unsupervised multi-scale clustering of single-cell transcriptomes to identify hierarchical structures of cell subtypes
**Article Type:**	Research
**Funding Information:**	National Institute of General Medical Sciences (R35GM142918) — Associate Professor Won-Min Song<br>Division of Microbiology and Infectious Diseases, National Institute of Allergy and Infectious Diseases (R21AI149013) — Associate Professor Won-Min Song, Associate Professor Christian Forst<br>Division of Microbiology and Infectious Diseases, National Institute of Allergy and Infectious Diseases (R01AI170112) — Associate Professor Christian Forst<br>National Institute on Aging (RF1AG074010) — Professor Bin Zhang<br>National Institute on Aging (U01AG046170) — Professor Bin Zhang
**Abstract:**	Cell clustering is an essential step in uncovering cellular architectures in single cell RNA-sequencing (scRNA-seq) data. However, the existing cell clustering approaches to discover the complex cellular landscapes in scRNA-seq are limited by user bias in supervised parameter selections and inherent resolution limits to shadow rare but meaningful cell subtypes. Here, we develop a multi-scale clustering (MSC) approach to construct sparse cell-cell correlation network for identifying de novo cell types and subtypes at multiscale resolution in an unsupervised manner. Based upon simulated, silver and gold standard data as well as real scRNA-seq data in diseases, MSC showed much improved performance in comparison to established benchmark methods, and identified biologically meaningful cell hierarchy to facilitate the discovery of novel disease associated cell subtypes and mechanisms.
**Corresponding Author:**	Won-Min Song<br>Icahn School of Medicine at Mount Sinai<br>New York, New York UNITED STATES
**Corresponding Author Secondary Information:**	
**Corresponding Author's Institution:**	Icahn School of Medicine at Mount Sinai
**Corresponding Author's Secondary Institution:**	
**First Author:**	Won-Min Song
**First Author Secondary Information:**	
**Order of Authors:**	Won-Min Song<br>Chen Ming<br>Christian Forst<br>Bin Zhang
**Order of Authors Secondary Information:**	
**Additional Information:**	
**Question**	**Response**

Powered by Editorial Manager® and ProduXion Manager® from Aries Systems Corporation

Are you submitting this manuscript to a special series or article collection?	No
**Experimental design and statistics**<br><br>Full details of the experimental design and statistical methods used should be given in the Methods section, as detailed in our Minimum Standards Reporting Checklist. Information essential to interpreting the data presented should be made available in the figure legends.<br><br>Have you included all the information requested in your manuscript?	Yes
**Resources**<br><br>A description of all resources used, including antibodies, cell lines, animals and software tools, with enough information to allow them to be uniquely identified, should be included in the Methods section. Authors are strongly encouraged to cite Research Resource Identifiers (RRIDs) for antibodies, model organisms and tools, where possible.<br><br>Have you included the information requested as detailed in our Minimum Standards Reporting Checklist?	Yes
**Availability of data and materials**<br><br>All datasets and code on which the conclusions of the paper rely must be either included in your submission or deposited in publicly available repositories (where available and ethically appropriate), referencing such data using a unique identifier in the references and in the "Availability of Data and Materials" section of your manuscript.<br><br>Have you have met the above requirement as detailed in our Minimum	Yes

Standards Reporting Checklist?	
GigaScience has policies and guidelines in place for the use of generative AI-writing tools such as ChatGPT. If you have used such writing tools to assist with writing the manuscript this must be declared and cited in the text. Authors should not list AI-writing tools and other AI-assisted technologies as an author or co-author and should acknowledge that they are fully responsible for text generated or refined by AI-writing tools.<p><br><br>A summary of use (particularly in the introduction or among methods) needs to be included at the end of the paper, and the outputs should also be included as a supplementary file hosted in GigaDB or other open repositories. Please read our guidelines for more information. <p><br><br>By submitting to GigaScience, you are aware of the journal's AI-writing tools policy, and if you have declared use of such tools below, you have acknowledged this where appropriate in your manuscript and have made a summary of use and outputs available. <p><br><b>AI-assisted writing tools have been used in the preparation of this manuscript?	No

Manuscript

Click here to access/download;Manuscript;manuscript.v19_no_hyperlink.pdf

Unsupervised multi-scale clustering of single-cell transcriptomes to identify hierarchical structures of cell subtypes

Won-Min Song[1,2*§], Chen Ming[4], Christian V. Forst[1,2,3], Bin Zhang[1,2]

[1] Department of Genetics and Genomic Sciences, Icahn School of Medicine at Mount Sinai, One Gustave L. Levy Place, New York, NY 10029, USA

[2] Mount Sinai Center for Transformative Disease Modeling, Icahn School of Medicine at Mount Sinai, One Gustave L. Levy Place, New York, NY 10029, USA

[3]Department of Microbiology, Icahn School of Medicine at Mount Sinai, One Gustave L. Levy Place, New York, NY 10029, USA

[4]Faculty of Health Sciences, University of Macau, Avenida da Universidade, Taipa, Macau, China

[*]First author

[§]Corresponding author:

Won-Min Song, Ph.D.

Associate Professor, Department of Genetics & Genomic Sciences

Member, Mount Sinai Center for Transformative Disease Modeling

Icahn School of Medicine at Mount Sinai,

1399 Park Avenue, Suite 4-429, New York, NY 10029,

Tel: (332) 243-7070, Email: won-min.song@mssm.edu

ABSTRACT: Cell clustering is an essential step in uncovering cellular architectures in single cell RNA-sequencing (scRNA-seq) data. However, the existing cell clustering approaches are not well designed to dissect complex structures of cellular landscapes at a finer resolution. Here, we develop a multi-scale clustering (MSC) approach to construct sparse cell-cell correlation network for identifying *de novo* cell types and subtypes at multiscale resolution in an unsupervised manner. Based upon simulated, silver and gold standard data as well as real scRNA-seq data in diseases, MSC showed much improved performance in comparison to established benchmark methods, and identified biologically meaningful cell hierarchy to facilitate the discovery of novel disease associated cell subtypes and mechanisms.

Keywords: multi-scale clustering, scRNA-seq, bioinformatics, similarity network

BACKGROUND

Single-cell sequencing enables the extraction of molecular features at the cellular resolution to elucidate heterogeneous cellular landscapes in various tissues under different conditions (e.g., development and disease). Cellular heterogeneity often manifests as distinct subtypes within certain cell types, and some of these are associated with certain conditions under a study. For examples, previous studies have identified expanded inflammatory monocytes in COVID-19 patients[1], microglia subtype associated with Alzheimer's Disease (AD)[2, 3], and exclusion of cytotoxic T-cells in tumors[4]. Unsupervised cell clustering analysis is crucial to capture these heterogeneous cellular landscapes in various conditions, especially to identify novel cell populations[5-7].

Graph-theoretic approaches have been popular for understanding clustering structures in scRNA-seq to identify meaningful subpopulation architectures. These graph-theoretic approaches often utilize k-nearest neighbor (kNN) network and its variant shared nearest neighbor (SNN) networks to construct the cell similarity networks[8-12], followed by the search for closely connected subnetworks by Reichardt-Bornholdt (RB) modularity (Q_{RB}) optimization. Q_{RB} is a variant of Newman's modularity (Q_N) modularity to quantify close connections within a subnetwork, compared to randomly connected subnetworks as the null reference[13]. A unique feature of Q_{RB} is the resolution parameter (γ) to control the resolution of the optimal clustering solutions [14] and Q_{RB} is defined as,

$$Q_{RB}(\gamma) = \frac{1}{2m_o}\sum_{c}(e_c - \gamma\frac{K_c^2}{2m_o})$$

where $\gamma > 0$ is clustering resolution parameter, m_o is the total number of links, e_c is number of links in cluster c, K_c is the sum of degree of nodes in cluster c. By choosing various γ, it allows the natural adaptation of multi-scale detection of cell clusters [10, 15, 16].

However, the multi-scale cell type architectures have been primarily explored by supervised approaches, thus guided by prior knowledge and user bias. These are exemplified by user guided selection of several crucial parameters such as kNN and γ. These parameters often takedefault values such as kNN=20 and γ=1 or are determined through visual inspection of the clustering results across different parameter values via UMAP or tSNE embedding[15, 16]. Also, the searches for cell subtypes are often hypothesis-driven. Based on prior knowledge, supervised subclustering is performed on cell types of interest to identify subtypes at finer resolutions [3, 4, 17], but it could also shadow discovery for novel subtypes with little or no prior knowledge.

Further, Q_{RB} suffers from the inherent resolution limit that fundamentally restrict the detection of fine clustering structures in a network. Within a network with m links, the resolution limit dictates the detection of closely connected subnetworks with an internal number of links, e_c, only upto $e_c = \sqrt{2m_o}$[18], and the resolution limit persists regardless of γ[19]. The dependency of resolution limit on m exacerbates in many kNN networks which often yield densely connected cell networks/subnetworks (i.e. $m_o \sim N_o^2$), and these could shadow rare but distinct cell subtypes present in the tissues.

Herein, we introduce an unsupervised multi-scale clustering (MSC) approach for single-cell transcriptome analysis to resolve the issues in supervised clustering approaches and the

resolution limit. Within MSC, we have developed a new cell similarity network method to construct sparse and clustered cell networks and improve the sparsity-driven resolution limit in the modularity optimization problem. We have also implemented a new top-down clustering approach to iteratively split a parent network into more coherent and compact subnetworks, and eventually construct a cell hierarchy as the data-driven model of cell types and subtypes to facilitate the novel cell population discovery.

We evaluate MSC by applying it to simulated data, golden standard data with known ground-truth clusters, and silver standard data with inferred cell types as the ground-truth clusters. Ground-truth clusters allow an objective performance comparison of MSC with widely used benchmark single-cell clustering methods such as SNN-based Louvain clustering approaches with varying γ in Seurat[10], SC3[20] and CIDR[21], which have been identified as among the best performing single-cell clustering methods[22, 23]. Then, we apply MSC to several disease scRNA-seq datasets from different tissue types to demonstrate its capacity to identify novel cell subpopulations and biological mechanisms. Overall, we present MSC as a valuable unsupervised single-cell transcriptome clustering method to understand complex cell architectures.

RESULTS

Overview of Multi-Scale Clustering (MSC) analysis framework

MSC consists of two major steps, including construction of cell similarity (also termed cell-cell interaction) network (CSN) and and top-down cell clustering on CSN (**Figure 1**). Firstly, MSC employs a novel locally embedded network (LEN) method to construct a sparse cell network without the needs to specify kNN (**Figure 1A**). For a similarity (or dissimilarity) metric of choice,

LEN utilizes a graph embedding technique on topological sphere[24] to deterministically identify the nearest neighbors (NNs) for each cell. These locally embedded nearest neighbors (eNNs) are identified by searching for high similarity cell pairs among the cell and its eNNs without edge crossing when drawn on a sphere. In turn, the ensemble of eNNs for all cells constitutes the locally embedded neighbor network (LEN; **Figure 1A**-I), followed by low quality edge filtering through evaluating low similarity and edge centrality (**Figure 1A**-II, III) (see **METHODS** for details of LEN construction).

Then, MSC employs a top-down clustering approach, iteratively splitting a parent cell network into more coherent and compact subnetworks to produce a cell hierarchical structure of cells. While different clustering solutions may emerge at different resolutions, we aim to identify the most granular clustering solution at each split, exploring cell subpopulations at progressively finer resolutions with each resolution. Specifically, we have developed *AdaptSplit*, an adaptive clustering method to search for the most granular clustering solution at each split. The child clusters from the split are compared to the parent for assessment of improvements in compactness (υ) and intra-cluster connectivity (λ) (**Figure 1B**-II; see **METHODS** for details). The iterative top-down split continues until no child cluster shows improved cluster qualities than its predecessors, completing the search for the cell hierarchy (**Figure 1B**-III). The cell hierarchy then informs data-driven biological insights into the cell subsets with distinct molecular characteristics (**Figure 1C**).

Performance Evaluation with Simulated Data

Simulated data are useful to evaluate performances of clustering methods by providing the ground-truth clusters and gain insights on how these methods behave under different scenarios by varying noises, cluster sizes and hierarchies[25]. However, there are currently no tools to simulate single-cell sequencing data with careful controls over hierarchical structures and noise parameters. To mitigate this, we utilized the multivariate Gaussian model, $\mathbf{X} \sim \mathcal{N}(\boldsymbol{\mu}, \boldsymbol{\Sigma})$, with Gaussian noises, $\boldsymbol{\epsilon}$, as the stochastic data generator, $\mathbf{X}' = \mathbf{X} + \boldsymbol{\epsilon}$. This framework allows us to instill various clustering structures including hierarchies by specifying the covariance matrix ($\boldsymbol{\Sigma}$) with a higher intra-cluster covariance than the inter-cluster covariance, and have been successfully utilized in our previous study[25]. Utilizing $\mathbf{X}'$, we simulated stochastic data with various scenarios including (I) a single-layer clustering structures with irregular cluster sizes (**Figure 2A**), (II) a two-layer clustering structures with regular cluster sizes to mimic cluster hierarchy (**Figure 2B**), and (III) a two-layer clustering structures with irregular cluster sizes (**Figure 2C**). The data were simulated with varying noises amplitudes (σ) and intra-cluster correlations (ρ_{in}) as the factors eluding the true clustering structures (see **METHODS** for details).

We performed several benchmark single-cell clustering methods on the simulated data along with MSC. The benchmark methods included SNN-based Louvain clustering across $\gamma \in [0.4,2]$[10] and consensus clustering-based SC3[20], while CIDR was omitted as the data simulation does not generate read counts. MSC was performed using Pearson's correlations amongst the samples to identify the clusters.

The performances of these clustering methods in capturing the ground-truth clusters in the simulated data were evaluated using metrics that can handle overlapping clusters[26], as MSC

yields overlaps in the parent-child clusters. To this end, we adopted inclusion rate (IR), equivalent to the precision measure showing correctly classified cells in an inferred cluster[26], coverage rate (CR), equivalent to the recall measure showing correctly classified cells in a ground-truth cluster[26], and detection accuracy (DA), equivalent to the accuracy measure to identify the best match between a ground-truth cluster and a inferred cluster (see **METHODS** for details).

From scenario I, we evaluated the clustering methods to detect clusters defined at different resolutions by varying the intra-cluster correlations (ρ_{in}). With σ=1, we observed that all clustering methods performed well for $\rho_{in} \geq 0.2$ by showing high IR, CR and DA overall (**Figure 2D-F**). However, when DAs are evaluated for different cluster sizes, we observed that the SNN-based and SC3 results were prone to miss many small clusters (size = 25) at ρ_{in}=0.2, while MSC exhibited high detection accuracies for the small clusters (**Supplemental Figure 4**).

From scenario II, we evaluated whether the clustering methods are capable of detecting multi-scale clustering structures embedded in two-layer cluster hierarchy of regular clusters (**Figure 2B**). We simulated the hierarchical structures by imposing the intra-cluster correlation coefficient for the inner-layer (L_{in}) clusters at ρ_{in} = 0.25, the outer-layer (L_{out}) clusters at ρ_{in} = 0.125, and the rest at ρ_o = 0, hence different layers have correlation coefficient difference $\Delta\rho$=0.125 as the hierarchical structure resolution parameter[25]. Overall, MSC was the only method capable of detecting the ground-truth clusters from the inner-layer (L_{in}) and outer-layer (L_{out}) simultaneously in two distinct regions of noises, $0 \leq \sigma \leq 0.4$ and $0.8 \leq \sigma \leq 1$ (**Figure 2G-I**). Interestingly, there was an intermediate noise region ($0.5 \leq \sigma \leq 0.7$) where MSC missed the

clusters a L_{out} while detecting the clusters at L_{in}. This was followed by the larger noise region ($\sigma \geq$ 1.1) where only the clusters at L_{out} were detected, while missing those at L_{in}. In contrast, none of the SNN-based results and SC3 results were capable of detecting both layers simultaneously. Particularly, regardless of different γ values, SNN-based results were not able to capture the clusters at L_{out} for $\sigma \leq 1$ (**Figure 2G-I)**. Rather, higher γ imposed lower detection accuracies for clusters at L_{out} for $\sigma > 1$ (**Figure 2I**). Similar results were observed when $\Delta\rho=0.25$ (**Supplemental Figure 5A-C**), and indicate these findings are applicable in broader hierarchical structure resolutions.

Scenario III showed similar qualitative trends to scenario II (**Figure 2J-L)**. MSC was the only method capable of realizing the cluster hierarchy while other methods were detecting either of the two layers in different noise regions. Yet, some quantitative differences were observed. The range of noise levels that MSC detects the ground-truth clusters from the both layers have substantially narrowed ($1 \leq \sigma \leq 1.1$, **Figure 2L**). These are particularly due to the worsened detection of clusters at L_{out} for $\sigma \leq 0.9$, in which MSC failed to detect the higher order clustering structures of the clusters at L_{in}. It is interesting that these higher order structures were detected in the presence of greater noises in $1 \leq \sigma \leq 1.1$. Similar results were observed at $\Delta\rho=0.25$ to indicate the robustness of the findings at different hierarchical structure resolutions (**Supplemental Figure 5D-F**).

Overall, the simulated study allowed exploring various clustering scenarios across varying noises, cluster coherence and presence of hierarchical structures. The results demonstrate the advantages in MSC for improved detection of small clusters and hierarchical clusters, and

improved resolution limits compared to the SNN-based clustering. The simulation study also outlines several clear limitations. At certain noise windows, MSC failed to detect the hierarchical structure. When noise levels are relatively low ($\sigma \leq 1$), all clustering methods including MSC tend to detect the more correlated inner clusters at L_{in}. On the other hand, larger noise levels ($\sigma \leq 1$) tend to favor the detection of the less correlated outer cluster at L_{out}. These suggest the roles of noises in determining detectable clusters, and warrant further studies.

Performance Evaluation with Gold Standard Data

We collected a number of gold standard data sets generated from independent studies, whose ground-truth clusters are known through model simulation under various scenarios, FACS-sorted cell populations and different ratio of mRNA mixtures from distinct cell lines[27, 28] (**Table 1**). Using the ground-truth clusters, we comparatively evaluated MSC clustering results from utilizing Pearson's correlations (denoted MSC:Correlation) with variable genes and Euclidean distances in PCA space (denoted MSC:Euclidean) (see **Methods** for gold-standard data processing details). The benchmark methods for comparisons included: SNN-based Louvain clustering with widely used resolution values at γ = 0.4, 0.8 and 1.2 (denoted SNN γ=0.4, 0.8, 1.2)[10], the best performing SNN-based results from all γ (denoted SNN Best), imputation-based CIDR[21] and consensus clustering-based SC3[20].

We first evaluated if AdaptSplit can effectively capture these clusters without iterative splits. Using the adjusted Rand Index (ARI) between the ground-truth clusters and the inferred clusters[29] as the quality metric. We observed that AdaptSplit from Pearson's correlations and Euclidean distances were robustly among the better performing methods, following after SNN γ

= 0.4 as the overall best performing method (**Figure 3A, B**). These sub-optimal outcomes from AdaptSplit were rather expected, as it is designed to search for the most coarse-grained clustering solution to allow detections of more coherent clusters as the subclusters in the successive splits. These are also reflected in the lower IRs by the large and coarse-grained clusters to include cells from several ground-truth clusters (**Figure 3C**). Yet, these ground-truth clusters were correctly classified to single clusters while the iterative splits took place to identify the multi-scale clusters, and these were reflected in the higher CRs (**Figure 3D**).

We observed the multi-scale clustering scheme by MSC further improved the overall cluster detections. The iterative splits in MSC eventually identified the ground-truth clusters accurately, and these are reflected in the high DA scores (**Figure 3E**) for both of MSC:Correlation and MSC:Euclidean, and outperformed the other benchmark methods in accurately detecting the ground-truth clusters.

Further, we observed LENs were consistently sparse across all gold standard data sets. The sparsity of a network can be formulated by the relationship, $m=c_sN_o$ where m is the total number of links, N_o is the number of cells, and c_s is a scaling factor to define the network sparsity. From the golden standard data sets, LENs showed $3 \leq c_s \leq 5$. On the contrary, SNN networks showed $28 \leq c_s \leq 40$, indicating LENs are substantially sparser than the SNN networks to facilitate the small yet meaningful cluster detections (**Supplemental Figure 6**).

Performance Evaluation with Silver Standard Data in 8k PMBC Data Set

We processed and analyzed scRNA-seq of 8,381 peripheral blood mononuclear cells (PBMC) from a healthy donor from 10x website, and performed and the benchmark clustering methods

(see **METHODS** for data processing details) to identify the cell clusters. The cell types were annotated by *SingleR* (v2.2.0)[30] with bulk RNA-seq of sorted immune cell populations, also known as the Monaco collection (GSE107011), as the reference transcriptome[31]. This identified 29 subsets of immune cells (B-cell, CD4/CD8 T-cells, NK cells, monocytes, dendritic cells) and progenitor cells in the data (**Figure 4A, B**; **Supplemental Data 1**).

Using the annotated cell types as a silver standard ground-truth clusters, we evaluated the performances of the various clustering methods to detect these immune cell types, and observed distinct differences in their clustering results. We observed that AdaptSplit results from Pearson's correlation (AdaptSplit:Correlation) and Euclidean distance in the PC space (AdaptSplit:Euclidean) identified coarse-grained clusters that mostly aligned with the major immune cell types, compared to the other benchmark results (**Figure 4C**). For example, B-cells and NK-cells were correctly identified as single clusters. Notably, correlation-based AdaptSplit correctly identified the myeloid cells into a single cluster, in contrast to the Euclidean distance-based results that differentiated several myeloid subpopulations such as dendritic cells and classical/intermediate monocytes (**Figure 4C**). On the other hand, the clustering results from SNN-based Louvain clustering in different resolutions and SC3 tended to over-split the clusters and NK-cells were the only major cell type identified as a single-cluster. In contrast, the clusters by CIDR tend to under-split the clusters and failed to discriminate NK-cells from T-cells (**Figure 4C**).

Overall, MSC was the most balanced method that avoided over- or under-fragmentation of the ground-truth clusters. Similar to the results from simulated data, the overall MSC performances

were characterized by slightly lower IRs than SNN-based clustering results due to the coarse-grained clusters, and the highest CRs and DAs to reflect the detections of the correct ground-truth clusters in probing the cell hierarchy (**Figure 4D, E**). These patterns were consistently observed for detecting the major immune cell types (**Figure 4D**) and subtypes (**Figure 4E**).

Further, MSC was among the best performing clustering to detect the immune cell subtypes (**Figure 4E**). Using Jaccard Index between the clustering results and the ground-truth clusters as the detection accuracy of individual immune subtypes (see **Methods** for details), MSC consistently detected largest numbers of immune subtypes across different detection thresholds. Overall, MSC can effectively detect major cell types and subtypes in unsupervised manner in real scRNA-seq data.

Applications to influenza and COVID-19 infected PBMC scRNA-seq: MSC identifies novel *CRBN/RBX1*-high platelet subpopulations in severe COVID-19

To assess the utility of MSC to study cellular landscapes in infectious diseases, we processed and analyzed single-cell transcriptome of 62,301 cells from 20 PBMC samples, comprised of 5 influenza infected patients, 11 COVID-19 infected patients with varying range of severity and 4 healthy controls from Lee *et al.* 2020 (see **METHODS** for data processing details)[1].

MSC clusters systematically identified several branches of immune/blood cell types associated with influenza and COVID-19 infections. Using the finalized cell type annotations (**Figure 5B;** see **METHODS** for cell type annotations; **Supplemental Data 2A**), the MSC cluster hierarchy (**Supplemental Data 2B, C**) captured the most of the major cell types in the clusters at the first split, and the child clusters subsequently compartmentalized into more distinct immune cell

subtypes (**Figure 5A-C**), characterized by enrichments of different disease conditions (**Figure 5D**). Particularly, MSC outperformed SNN-based Louvain clustering at varying resolutions in detecting the annotated cell types and subtypes with greater IR, CR and DA (**Figure 5E**; **Supplemental Figure 8**). We note that other benchmark methods (SC3 and CIDR) were not successfully executed due to the requirements for large computational resources by these methods, hence were omitted in the comparisons.

Several unique cell subtypes identified by MSC were associated with severe COVID-19 samples. Many cell clusters showed preferential enrichments for individuals from specific disease conditions (**Figure 5F-J**; **Supplemental Data 2D**). One example is the expansion of platelets in severe COVID-19 samples (**Figure 5J**), comprised of *CRBN/RBX1*-high (M33) and *IFITM3*-high (M34) subpopulations (**Supplemental Figure 9**). Recently, Lenalidomide, a CRBN/RBX1 inhibitor, has shown protective roles in multiple COVID-19 infected myeloma patients against progressing into severe infections[32], and suggests the emergence of this particular platelet subpopulation may drive the disease severity in COVID-19 infection. On the contrary, *IFITM3* is IFN-induced antiviral protein and its expressions are shared with monocytes/macrophages. Polymorphism in IFITM3 has been associated with COVID-19 and severity[33], its expression inhibits COVID-19 infection[34] and these suggest M34 is a protective platelet subtype under pro-inflammatory environments. Overall, the MSC identified distinct platelet subtypes with functionally distinct characteristics, and these warrant further investigations for novel COVID-19 therapeutics.

Applications to breast cancer single-cell atlas: MSC identifies a novel protective endothelial subset in breast cancer

We expanded MSC applications to a large-scale study of breast cancer single-cell transcriptomes to explore heterogeneous tumor microenvironments and novel cell subtypes in solid tumors. Specifically, we performed MSC on single-cell transcriptome atlas of breast cancer by Wu *et al.* 2021[35], encompassing 26 breast cancer primary tumors of diverse subtypes by hormonal status (estrogen receptor (ER), progesterone receptor (PR) status), Her2 signaling status (Her2 amplification/deletion) and by molecular PAM50 subtyping[35]. This study has identified major cell types and the subsets through adapting supervised approaches to infer known cell types by xCell[36] and subcluster within known major cell types by SNN-based Louvain clustering in Seurat[35] (**Supplemental Data 3A**).

After quality controls (QC; see **METHODS** for data processing details), we processed 92,232 cells, analyzed and enumerated distinct cell populations. Firstly, we performed MSC and SNN-based clustering at varying resolutions (γ=0.4, 0.8 and 1.2) (**Figure 6A, B**), and compared the clustering results to the annotated major cell types and subsets from the published study as the silver standard ground-truth clusters (**Supplemental Data 3B-D**). We remark that SC3 and CIDR could not be carried out due to their excessive memory requirements. The first-split cell clusters from MSC readily captured the major cell types without supervision, while SNN-based clustering requires the fine-tuning of the resolution (**Figure 6A**). Further, MSC consistently detected higher numbers of the ground-truth clusters of major cell types and subtypes, compared to the SNN-based Louvain clustering (**Figure 6B**).

As the cell types and subtypes identified by Wu *et al.* 2021 are primarily by supervised approaches[35], we anticipated that unsupervised clustering results by MSC could potentially

identify novel cell subtypes which were overlooked in the supervised approaches, and provide insights to the breast cancer biology. To this end, we leveraged the Jaccard index (JI) as a normalized overlap metric to assess MSC-unique clusters with low overlaps against the annotated cell types/subsets, and the SNN-based Louvain clusters at different resolutions with JI < 10% (**Supplemental Data 3E**, **F**) . These yielded a large number of MSC-unique clusters, primarily as subtypes within major cell types in the cell hierarchy(**Figure 6C**).

Among these, M138 captured a unique endothelial subset that was overlooked in the previous study (**Figure 6D**). While the previous study identified the subsets characterized by ACKR1, LYVE1, CXCL12 and RGS5 (right, **Figure 6D**), M138 is a unique subset of capillary endothelial cells (ECs) characterized CA4 expressions (**Figure 6E**)[37, 38], and is present in ER+, Her2+ and triple-negative breast cancer (TNBC) subtypes with enrichment of cells from TNBC, compared to the pool of all ECs (**Figure 6F;** FET p-value = 8.71E-5, EFC = 1.62).

We observed that presence of M138 EC subset in breast cancers is robustly predictive of good prognosis. To estimate the relative abundance of M138 EC subset, we identified M138-specific marker expressions (**Figure 6E**; **Supplemental Figure 10**; see **Methods** for marker identification), and performed single-sample Gene Set Enrichment Analysis (ssGSEA) score[39] as the proxy for the relative abundances of M138 ECs in METABRIC bulk transcriptome cohort[40] (see **Methods** for METABRIC data processing). Stratifying patients by median M138 ssGSEA scores, stronger enrichments of M138 cells were significantly associated with good prognosis in ER+, TNBC and all METABRIC cohort with logrank p-value < 0.05 (**Figure 6G**). We also observed higher expressions of several M138 marker genes were significantly associated to better relapse-

free survival in independent breast cancer transcriptomes from previously published studies[41] (**Supplemental Figure 11**). Reported functions of the marker genes in the literature are also supportive of the protective roles of the capillary ECs against breast cancer. These include TIMP4 (an inhibitor of capillary EC invasion[42]), TNMD (an angiogenesis inhibitor), ATOH8 (transcription factor to regulate endothelial cell proliferation[43]), AQP7[44] and LIPE[45] (regulators of fatty acid metabolism).

Overall, these results demonstrate that MSC can effectively facilitate the discovery of novel cell subsets in exploratory studies, as exemplified by M138. M138 signifies a unique capillary endothelial subset characterized by CA4 over-expressions, and its presence is robustly predictive of good prognosis in breast cancer.

Computational complexity of MSC

We analyzed the overall computational complexity, $\mathcal{O}(n) \sim n^{\eta}$ (η is the scaling factor), of different methods through measuring the runtimes of MSC and the benchmark methods scales across data with varying sizes (*n*). We curated a set of publicly available scRNA-seq data whose sizes vary from small sized cohorts (< 10,000 cells) to atlas-sized cohorts (> 100,000 cells). We utilized parallel computations with 8 cores for methods with available parallel functionalities (SC3 and MSC), and assigned 8GB of memory per each core. Overall, MSC is a scalable clustering method to analyze from small to atlas-sized single-cell cohorts with feasible computational resources on personal machines. MSC and SNN-based clustering were among the most scalable methods showing η ~ 1.3, while SC3 showed η ~ 2 and CIDR showed η ~ 2.7 (**Supplemental Figure 3A**).

The memory usage was also a crucial factor for applicability. While memory usages by MSC and SNN-based clustering scaled similarly across different data sets with tractable < 50GB usages, CIDR and SC3 failed to perform due to excessive memory usage for 10,000 > cells (**Supplemental Figure 3B**). With access to high performance computing, MSC can be further parallelized to improve the overall runtime (see **Supplemental Results** for detailed analysis).

DISCUSSION

In this study, we have developed a new multi-scale cell clustering (MSC) approach. Firstly, we introduced a novel method for constructing cell similarity network, named LEN. LEN is a deterministic method that does not require user-defined parameters such as kNN and guarantees the generation of sparse cell networks owing to the utilization of embedding the nearest neighbors on a topological sphere, which imposes a hard upper bound on the number of links in the locally embedded network, m_{local}, by Euler's relation, where $m_{local} \leq 3(N_{local}-2)$ for such embedded networks[24]. This upper bound implies the local sparsity (c_s^{local}) is restricted upto 3, and this translated to the global sparsity in $3 \leq c_s \leq 5$ (**Supplemental Figure 6**).

Such sparsity can inherently improve the cluster detection resolution limit via lowering the overall number of links (m_o), restricting the detection of cell clusters with the number of internal links, $e_c = \sqrt{2m_o}$[18]. Indeed, we observed improved detection of small clusters from the simulation studies, compared to the SNN-based clustering results (**Supplemental Figure 4**).

We also introduced a new multi-scale clustering (MSC) algorithm, which detects meaningful cell cluster hierarchy in a LEN, and improves detection accuracy of the underlying clustering structures in the single-cell transcriptome data. The performance of MSC was evaluated in

simulated data by multivariate Gaussian models with noises. Overall, MSC outperformed other benchmark single-cell clustering methods by detecting the true clusters with greater accuracy under various scenarios simulating presence of cluster hierarchy, varying noise amplitudes, and irregular cluster sizes (**Figure 2**).

Interestingly, MSC was the only method capable of simultaneously detecting clusters at different hierarchical layers (**Figure 2G-L**). The top-down iterative clustering approach allowed detection of the nested, inner layer clusters at L_{in} after successfully detecting the outer layer clusters at L_{out}. However, depending on the cluster size regularity, different windows of noise amplitudes allowed the simultaneous detection of clusters at both layers. This is in contrast to the kNN-based clustering results detecting only one layer of clusters, regardless of the varying cluster resolution parameter, γ. Rather, the noise amplitudes were the main determinants of the kNN-based clustering results. The lower noise amplitudes favored detection of the inner layer clusters at L_{in}, and higher noise amplitudes favored the outer layer clusters at L_{out}. Overall, these exemplify the benefits of multi-scale cluster detection in MSC by the top-down approach, otherwise controlling for γ alone is not capable of exploring the cluster hierarchy, hence the true multi-scale structures that are often present in single-cell transcriptomes.

Further, we showed that MSC consistently outperformed other benchmark single-cell clustering methods, showing higher inclusion rates, coverage rates, and detection accuracies of the ground-truth clusters based on gold standard benchmark data sets with known ground-truth clusters from FACS sorting, or mRNA mixtures from different cell lines from different scRNA-seq platforms. (**Figure 3**).

These superior performance of MSC is evident when applied to detect cell types in real-world scRNA-seq data from various diseases and tissues. Using inferred cell types as the silver standard, MSC detected the highest number of major cell types and their subtypes in PBMC from healthy donors (**Figure 4**), PBMC from influenza and COVID-19 infected patients (**Figure 5**) and breast cancer (**Figure 6**). We demonstrated that MSC is capable of identifying novel cell populations associated with various disease etiologies. From the PBMC of influenza and COVID-19 infected patients, MSC identified two platelet subpopulations expanded in severe COVID-19 patients, namely, *CRBN/RBX1*-high (M33) and *IFITM3*-high (M34) cells. Particularly, the over-expression of CRBN/RBX1 exemplified the potential therapeutic implication of Lenalidomide, a CRBN/RBX1 inhibitor, in severe COVID-19 patients, where CRBN/RBX1 inhibitor were reported as protective against severe COVID-19 in several myeloma patients whose standard-of-care included Lenalidomide[32].

MSC also facilitated detection of novel cell subtypes in breast cancers. While the supervised subclustering of the endothelial cells in the published study remarked four subsets characterized by *ACKR1*, *LYVE1*, *CXCL12* and *RGS5* expressions[35], MSC readily identified another distinct capillary EC subset characterized by CA4 expressions. Enrichment of the capillary EC subset was robustly associated to good prognosis in multiple breast cancers bulk transcriptome cohorts, and demonstrate the utility of MSC for novel cell subset discovery in diseased tissues.

CONCLUSIONS

We have presented MSC as a new single-cell multi-scale clustering framework by adopting a novel algorithm for constructing cell similarity network and a multi-scale clustering approach.

MSC shows superior performance over some state-of-the-art single-cell clustering methods through an objective evaluation based on a broad spectrum of simulated and real-world data with ground-truth clusters. MSC is an invaluable tool for advancing discoveries in disease associated cell populations in single-cell sequencing data.

METHODS

Overview of Multi-Scale Clustering (MSC)

MSC is a two-step process consisting of cell-cell similarity network construction by locally embedded network (LEN), followed by iterative top-down splits of the cell network to realize a hierarchy of parent and child clusters (**Figure 1**).

I. Locally embedded network (LEN) construction: In many complex real-world networks, the network topologies amongst a node and its immediate neighbors are often planar, such as star graphs and wheel graphs[46]. Further, planarity networks are sparse networks due to the topologically enforced upper limit on the number of links, $\mathrm{m} = 3(\mathrm{N}-2)$, where *N* = number of nodes, by the Euler's relation[46]. Taken together, this implies that the planarity constraint could be sufficient to realize the true interacting neighbors for a node and guarantee sparsity in the resulting local network. Indeed, we have translated the planarity constraint to construct gene interaction networks[47], and these networks have been validated to capture true gene interactions and facilitated discoveries of novel regulators of disease pathways such as cancers[48-50], asthma[51], neurodegenerative diseases[52-54] and infectious diseases[55]. Herein, we sought to translate the utility of the planar network to effectively construct clustered and sparse cell similarity networks.

(i) Search for locally embedded neighbors for individual cells: We leveraged the planarity constraint to determine the nearest neighboring cells to construct sparse and clustered cell similarity networks. Using a cell similarity of choice, S, LEN first searches for k most similar cells (NN_k^i), $NN_k^i = \{\mathrm{j}|\mathrm{S(i,j)} \leq S_k(\mathrm{i})\}$ where $S_k(\mathrm{i}) = k$th nearest similarity from each cell, i. Then, a planar maximally filtered graph (PMFG) amongst the cells in NN_k^i is constructed to identify a planar graph, P_k^i, with the maximal number of links, $3(NN_k^i - 2)$, that maximize the overall similarity among the connected cells[24] (**Figure 1A**-I). As we gradually increase k in $[3, \sqrt{N_o}]$ (N_o = number of cells in the data set), the neighbors immediately connected to i in P_k^i saturates to a plateau at k' to yield the finalized nearest neighbors, $NN^i_{k'} = NN^i$ as the locally embedded neighbors. In practice, we find $k' \sim \log(N_o)$ to reach the plateau. Finally, the locally embedded network of each cell, P'^i, is realized by connecting to its embedded neighbors, NN^i, and the overall locally embedded network is constructed through the ensemble across all cells, $G' = \cup_i P'^i$.

(ii) Low quality link screening: As the local embedding explores directly linked cells, i.e. the 1st order connections, the higher order network structures such as local clustering and node centralities are overlooked in the initial network, and as results, low quality links to shadow the higher structures can be introduced in G'. Further, scRNA-seq are often noisy and may result in introducing low quality cell-cell links to further shadow the network topology. To mitigate these, we have implemented link screening steps to filter out links with low similarities and low centralities:

- *Low similarity screen*: The sparsity of single-cell transcriptome is a major source of noises and is detrimental to inferring the cell clustering structure[16, 56]. To this end, we observed the single-cell transcriptome sparsity manifested into the varying number of commonly expressed genes between two cells across a broad range, and this affected the pairwise cell similarities, S_{ij}, to vary dependently on the size of commonly expressed genes (**Supplemental Figure 1**). Thus, we modeled the relationship between the number of common genes and the cell-cell similarity with LOESS regression[57], and identified the noisy links as the outliers from the fitted curve. Specifically, we calculated the proportion of commonly expressed genes between two cells over the union of all expressed genes in both cells, J_{ij}. Then, we evaluate the relationship between J_{ij} and S_{ij} via LOESS regression to identify the sparsity-dependent similarity thresholds as the two standard deviations away from the fitted mean (left, **Figure 1A**-II).
- *Low centrality screen*: The ratio of shared nearest neighbors between two cells, M_{ij}, is a useful 2nd order centrality measure to evaluate the local clustering structures[58]. We calculate the M_{ij} for all pairs of connected cells in G', and contest the lower quantile cell pairs by the cell-cell similarity for removal. For each contested cell pair, we evaluate if removal of the cell link improves M_{ij}. If improved, the cell link is removed and this removal occurs iteratively for all contested cell pairs. The cell link removal iteratively occurs for the similarity-sorted cell links (middle, **Figure 1A**-II).

Altogether, the local embedding and link screening yields the finalized locally embedded network (LEN), G_o.

II. Iterative top-down clustering: The clustering structure in G_o is probed by iteratively splitting parent networks into several child clusters with improved cluster qualities including connectivity (i.e. coherent clusters) and compactness (i.e. tightly connected clusters). The iterative splits terminate when no further child clusters are discovered with improved cluster qualities, and eventually identify a cell hierarchy of parent and child clusters as the data-driven model of cellular architecture in the single-cell transcriptome.

<u>Adaptive network split (*AdaptSplit*) to search for granular clustering solutions:</u> Each split purposely searches for the most granular clusters so that the child clusters represent the immediate subtypes of its parent cell type. These granular clusters may be defined at varying resolutions, dependent on the parent network's topology. To address this, we devised *AdaptSplit* method to adaptively search for the granular clustering solution. Specifically, *AdaptSplit* first identifies clustering solutions in $\gamma' \in (0,2]$ on a parent network, $G_o(V_o,E_o)$, by Leiden's clustering[59]. The range of γ' is purposely set to explore the clustering solutions around the neutral resolution, γ'=1[13, 14], and include widely used $\gamma' \leq 1.2$ in single-cell clustering[10, 15].

We hypothesized that a stable, granular clustering solution should maintain stable intra-cluster connectivity at low resolutions (i.e. low γ' values). To test this, we examined the overall intra-cluster connectivity, $K_{in} = \sum_{i,j\in\Theta_c} A_{ij}$ where $A_{ij} = 1$ if *i* and *j* are connected for a clustering solution by Louvain clustering at γ', $\Psi(\gamma = \gamma') = \{\Theta_c | \Theta_c \subseteq V_o\}$ with the disjoint conditions ($\Theta_c \cap \Theta_{c'} = \emptyset, c \neq c'$ and $\bigcup_c \Theta_c = V_o$), to maintain stable values for a range of γ' values. Typically, more fragmented and smaller clusters yield smaller K_{in}, and often, stable clustering solutions manifest as stable K_{in} to across a certain range of $\gamma' \leq \gamma \leq \gamma''$, at the break points, γ' and γ''

(**Figure 1B**-I). The break points are systematically identified by logistic regression to fit step functions incorporating the discrete K_{in} values at different γ regimes with *rpart* R package (v4.1.19). The first regime, $\gamma < \gamma'$ (highlighted in **Figure 1B**-I), is identified as the stable clustering solutions with granular clusters, and the clustering solution with median resolution in the regime, γ_f, is selected as the final clustering result for *AdaptSplit*.

Comparative evaluations of child clusters to its parent clusters for cluster quality improvements: Then, the child clusters are compared to its respective parent cluster for improved cluster qualities. This comparison assumes that the split is meaningful only if it yields more well-defined clusters than the parent cluster, and this rationale serves to determine the termination when no further improved child clusters are detected. Specifically, we utilize (I) compactness and (II) intra-cluster connectivity as the cluster quality metrics:

(I) Compactness comparison: We have previously developed Multi-scale Embedded Gene co-Expression Analysis (MEGENA) that utilizes an iterative top-down clustering approach on planar gene networks[47]. Within MEGENA, we established a cluster compactness measure, $\upsilon(\alpha) = \overline{SPD}/\log(N_c)^{\alpha}$, where $\overline{SPD}$ is the average of shortest path distances of all cell pairs in a network, α is the compactness scaling parameter, and N_c is the number of nodes in cluster *c*. When comparing compactness of child clusters to the parent cluster, we showed that $\upsilon(\alpha)$ can effectively identify compact child clusters, and detect biologically meaningful cluster hierarchy of parent and child clusters[47]. However, its direct translation to LEN is limited as α varies in a narrow range for planar networks[47, 60]. To this end, we adapted the compactness measure by fine-tuning α. In MSC

workflow, α serves as the scaling parameter for $\overline{SPD}$, and determines the role of cluster sizes in calculating the compactness. To identify the suitable α for a given network, we randomly sample 100 subnetworks by propagating 3-layer neighborhoods of 100 randomly chosen nodes. Standardizing $\upsilon\left(\alpha_{o}\right) = 1$ as the normalized compactness where α_o serves as the reference scaling parameter, we can derive the expression for the reference scaling parameter as $\alpha_o = \log(\overline{S\ \square\square})/\log(\log(N_c))$. In N_c -vs- α_o plot, α_o converged towards a constant value < 2 (See **Supplemental Figure 2**) in most cases, and this convergent value was used as the compactness scaling parameter for parent-child cluster comparisons.

(II) Intra-cluster connectivity comparison: In addition to the compactness comparison between the parent and child clusters, we evaluated the significance of intra-cluster density among the child clusters to ensure probing for coherent clustering structures. Within each parent cluster, *p*, the intra-cluster connectivity of each child cluster, *c*, can be defined as: $\lambda_c = e_{cc}^{p}/e_{c}^{p}$, where e_{c}^{p} is the number of links connected to any cells in *c*, e_{cc}^{p} is the number of links connecting cells within cluster *c*.

We evaluated the statistical significance of λ_c by randomly permuting 10% cells across different child clusters 100 times, and calculated the permuted intra-cluster density $\lambda_{cc}^{'}$ as the random reference values to calculate the significance p-value. With the density p-value < 0.05, the child clusters were identified as significantly coherent.

Disease group enrichment analysis

We performed Fisher's Exact Test (FET) to evaluate enrichment of individual cell clusters in individual samples. A sample was deemed enriched for a cell cluster if the respective FDR adjusted FET p-value (FET FDR) < 0.05. Then, for each disease condition and each cell cluster, we calculated the proportion of samples showing the enrichments, and labeled cell clusters where at least 50% of samples from a respective disease condition as enriched.

Data Simulation

We generated simulated data using multivariate Gaussian model, $\mathbf{X} \sim \mathcal{N}(\boldsymbol{\mu}, \boldsymbol{\Sigma})$, $\mathbf{X} \in \mathbb{R}^N$ with $\boldsymbol{\mu} = \mathrm{E}(\mathbf{X})$ is the N-dimensional mean vector, and $\Sigma_{ij} = E\big((X_i - \mu_i)(X_j - \mu_j)\big)$ is the covariance between *i*th and *j*th values in $\mathbf{X}$. Then, we added data Gaussian noises ($\epsilon \sim \mathcal{N}(\mu, \sigma)$ with $\mu = 0$) to this model, hence $\mathbf{X}' = \mathbf{X} + \boldsymbol{\epsilon}$. Throughout the simulations, we also imposed $\Sigma_{ii} = 1$ and $\mu_i = 0$ for all i to ensure the covariance becomes synonymous with the correlation, $\boldsymbol{\rho}$.

In this formulation, we have customized the correlation matrix to impose several clustering scenarios in the simulated data.

These scenarios include:

(I) A single-layer of clustering structure with irregular cluster sizes across varying intra-cluster correlations (**Figure 2A**): While fixing $\sigma = 1$, we varied intra-cluster correlations, for $\mathrm{i}, \mathrm{j} \in \mathrm{c}$ for some cluster *c*, $\rho_{ij} = \rho_{in} \in [0.1, 0.8]$ and set the inter-cluster correlation at 0 ($\rho_{ij} = \rho_{out} = 0$ if i and j do not belong to a same cluster). Heterogeneous cluster sizes were imposed, including sizes of 25 (12 clusters), 50 (6 clusters) and 100 elements (3 clusters).

(II) A hierarchical clustering structure of regular cluster sizes (**Figure 2B**): We defined two layers of clustering structures by imposing different correlation strengths at different layers. Specifically, we started by defining 21 seed clusters of size 50, constituting the inner layer clustering structure (L_{in}), with an intra-cluster correlation, ρ_{in}. Then, we adjoined six seed clusters to construct the outer layer clustering structure (L_{out}), with a weaker intra-cluster correlation, ρ_2 with $\rho_1 > \rho_2 > 0$. The inter-cluster coefficients were fixed at 0. We explored two different sub-scenarios by controlling $\Delta\rho = \rho_1 - \rho_2$ at 0.125 and 0.25, to simulate different definitions in the hierarchy. Having defined the hierarchical correlation matrix, we varied the amplitude of the Gaussian noises via $\sigma \in [0.1,2]$.

(III) A hierarchical clustering structures of irregular cluster sizes (**Figure 2C**): Similar to scenario II, we imposed two-layer hierarchy with $\Delta\rho = 0.125 \text{ and } 0.25$, where the seed clusters were heterogeneous in sizes at L_{in}, including 12 clusters of size 25, 6 clusters of size 50,and 3 clusters of size 100. At L_{out}, we imposed the higher layer clustering structure by merging 4 seed clusters of size 25, 2 seed clusters of size 50, and 1 seed cluster of size 100 with ρ_2. Similar to scenario II, we generated $\Delta\rho = 0.125, 0.25$ with varying Gaussian noise amplitudes, $\sigma \in [0.1,2]$.

For each set of parameter, we generated 10 random replicates, across 500 features. While each scenario generates data across ~1000 cells, the number of features was deliberately selected to be much smaller than the number of cells, as observed many scRNA-seq studies[16]. These simulations were performed using *MASS* R package (v7.3-57).

Evaluation Metrics

As MSC yields overlapping clusters from its parent-child cluster hierarchy, we evaluated the agreements of clustering results with the true clusters by adopting the evaluation metrics for overlapping clusters. Traditionally, for a clustering results, $\Psi' = \{\Theta_i' | i = 1, \ldots, k'\}$, and a ground-truth clusters, $\Psi^o = \{\Theta_j^o | \mathrm{j} = 1, \ldots, k^o\}$, precision and recall were used to evaluate performances of non-overlapping cluster results. Precision represents the number of correctly classified cells over the volume of a result cluster (i.e. $\mathrm{P}(\Theta_i', \Theta_j^o) = |\Theta_j^o \cap \Theta_i'| / |\Theta_i'|$), and recall is the number of correctly classified cells over the volume of ground-truth (i.e. $\mathrm{R}(\Theta_i', \Theta_j^o) = |\Theta_j^o \cap \Theta_i'| / |\Theta_j^o|$)[61]. Their extensions to overlapping clusters have been proposed by El Ayeb *et al.* 2022, as inclusion rate and coverage rate, respectively[26].

Briefly, inclusion rate (IR) evaluates the embeddedness of the result clusters to the ground-truth clusters. For each result cluster, $\mathrm{IR}(\Theta_i') = \max_j \mathrm{P}(\Theta_i', \Theta_j^o)$ defines the individual IR. Then, the overall IR is defined as the weighted sum of individual IR, $\mathrm{IR}(\Psi') = \sum_i \mathrm{IR}(\Theta_i')|\Theta_i'| / \sum_i |\Theta_i'|$. On the other hand, the coverage rate (CR) evaluates the embeddedness of the ground-truth clusters, and the individual CR is $\mathrm{CR}(\Theta_j^o) = \max_i \mathrm{R}(\Theta_i', \Theta_j^o)$. Then, the overall CR is $\mathrm{CR}(\Psi^o) = \sum_j \mathrm{CR}(\Theta_j^o)|\Theta_j^o| / \sum_j |\Theta_j^o|$.

IR and CR were shown to be highly complementary, where IR is an indicator of how similar the result clusters are to the ground-truth, and CR is an indicator of how well the ground-truth clusters are represented in the result clusters[26]. However, CR values are inflated when the clustering results are under-segmented, and IR values are inflated when the clustering results are over-segmented. To this end, we devised an cluster accuracy measure to handle overlapping clusters. For each results cluster and ground-truth cluster, we calculated the ratio between their

intersection and union, known as Jaccard Index (JI), as $\mathrm{JI}\left(\Theta_i', \Theta_j^o\right) = |\Theta_i' \cap \Theta_j^o|/|\Theta_i' \cup \Theta_j^o|$. JI yields $\mathrm{JI}\left(\Theta_i', \Theta_j^o\right)$=1 if $\Theta_i' = \Theta_j^o$, and $\mathrm{JI}\left(\Theta_i', \Theta_j^o\right) = 0$ if there is no overlap. In analogy with CR, for each ground-truth cluster, we then defined the individual detection accuracy (DA) as the ideal overlap with the clustering results, $\mathrm{DA}\left(\Theta_j^o\right) = \max_i \mathrm{JI}\left(\Theta_i', \Theta_j^o\right)$. Then, the overall DA is $\mathrm{DA}(\Psi^o) = \sum_j \mathrm{DA}\left(\Theta_j^o\right)|\Theta_j^o|/\sum_j |\Theta_j^o|$. We used IR, CR and DA jointly to evaluate the concordance between the clustering results and ground-truth clusters.

Data processing for single-cell transcriptomes of gold standard data, Lee *et al.* 2020 (influenza/COVID-19 infected PBMC) and PBMC 8k data

We performed rigorous data pre-processing and quality controls on scRNA-seq using Seurat workflow[10]. First, we removed low-quality cells with mitochondrial reads > 20%, median absolute deviation (MAD) > 3 and average count > 0[62, 63]. The doublets were identified by DoubletFinder[64] and removed. The dropout reads were inferred using Adaptively thresholded Low-Rank Approximation (ALRA)[65]. The filtered data will then be normalized and log-transformed by SCTransform[66]. Where applicable, we integrated the single-cell transcriptome across different conditions, individuals or batches by canonical correlation analysis (CCA)[67].

Then, we selected highly variable genes as the features for cell clustering by calculating gene dispersions. Using *modelGeneVar()* function from scran package[63], we calculated biological variances of individual gene expressions from the log-normalized, pre-processed data by modeling mean-variance curve as the technical variance[62]. We selected genes with biological variance p-value < 0.05 as the variable features for cell clustering. The Pearson's correlation across the selected features was used to calculate the cell similarity and perform MSC. The top

20 principal components (PCs) from the selected features were used to calculate the Euclidean distances.

Cell type identification in PBMC 8k: The cell types were annotated by applying *SingleR* (v2.2.0)[30] with bulk RNA-seq of sorted immune cell populations, also known as the Monaco collection (GSE107011), as the reference transcriptome[31]. The Monaco collection data was provided through *celldex* R package (v1.6.0)[30], and accessed through *MonacoImmuneData()* function.

Cell type identification in *Lee et al.* 2020: Similar to 8k PBMC data set, most of the major cell types were annotated by *SingleR* (v2.2.0)[30] by using the Monaco collection as the reference[31] through *MonacoImmuneData()* function in *celldex* R package (v1.6.0)[30]. However, the Monaco collection included immune cells only, erroneously annotated many cells as progenitors, expected to be present at 1-2% in PBMC under normal circumstances and missed out on detecting platelets and red blood cells as reported in Lee *et al.* 2020[1] (**Supplemental Figure 7**). To this end, we utilized human primary cell atlas (HPCA)[68], a microarray collection of broader blood cell types, as the reference to supplement the cell type annotations (**Figure 5B**). Similar to the Monaco collection, HPCA was accessed through *HumanPrimaryCellAtlasData()* function in *celldex* R package.

Data processing and analysis for Wu *et al.* 2021 breast cancer single-cell transcriptome atlas: Wu *et al.* 2021 data included over 90,000 cells, and the several steps in data pre-processing applied in gold standard, Lee *et al.* 2020 and PBMC 8k data sets were computational prohibitive. These include dropout read imputations by ALRA, generation of integrated and normalized gene expression data by CCA, and calculation of cell similarity by Pearson's

correlation across the selected features. To this end, we performed a separate data pre-processing using computational efficient reciprocal PCA (RPCA) framework in Seurat v5 workflow[10], and the Euclidean distances in RPCA-based reduced dimension (top 50 PCs) was used to perform MSC. Specifically, we performed:

<u>Data processing and marker analysis:</u> The raw count matrices of single-cell transcriptomes across 20 samples from Wu *et al.* 2021[35] were downloaded from the Broad Single-Cell Portal (https://singlecell.broadinstitute.org/single_cell/study/SCP1039). We removed low-quality cells with mitochondrial reads > 20%, median absolute deviation (MAD) > 3 and average count > 0[62, 63]. The doublets were identified by DoubletFinder and removed[64]. Considering the large number of cells (~100,000 cells) and samples to perform the integration of samplewise single-cell transcriptomes, we utilized a fast implementation of CCA, reciprocal PCA (RPCA)[10] in Seurat v5 workflow (v5.1) in R (v4.2.0) to integrate top 50 PCs across different samples to embed them into a common reduced dimension. UMAP embeddings were subsequently calculated from the RPCA integrated coordinates for further analysis. In tandem, we normalized the samplewise single-cell transcriptomes by SCTransformation[66] approach using “SCTransform()”in Seurat v5, and the normalized expressions were re-corrected by synchronizing the median UMI across different samples by “PrepSCTFindMarkers()” in Seurat v5 workflow. The re-corrected data were utilized for calculating cluster markers by adopting MAST framework[69] in “FindMarkers()”. Ribosomal, mitochondrial rates and cellwise UMI counts served as the latent variables, and markers were identified by FDR < 0.05, and requiring a greater proportion of cells in a cell cluster/group of interest to express a marker gene than the control cell groups.

M138-specific marker identification: We first compared M138 against the rest of endothelial cells (ECs) using “FindMarkers()” with MAST framework as implemented in Seurat v5 workflow. We applied FDR < 0.05 and required the marker genes to be expressed in at least 10% of cells in M138, and expressed in less than 5% of the rest of ECs. We then checked if M138-specific markers within ECs were also endothelial markers by comparing their expressions in other major cell types. Similarly, we required the marker genes to be expressed in at least 10% of ECs, and expressed in less than 5% of the rest of cells.

Enrichment analysis of M138-specific program in bulk samples with good prognosis**:** We downloaded the raw count matrix for 1,080 primary tumor samples of breast cancers from The Cancer Genome Atlas (TCGA) RNA-sequencing experiments[70], and performed counts per million (CPM) normalization, followed by Trimmed Mean of M-values scaling[71] and log2(x+1) transformation using edgeR R package (v3.38.1). We then adjusted for the batch variables (data generating center, date, and machine as identified in TCGA barcode) and patients’ age by generalized linear model (*glm()* in **stats** R package, v4.2.0). Similarly, we downloaded the log-normalized gene expression data of 1,974 samples from the METABRIC cohort[40], and adjusted for batch and age by generalized linear model. Then, we utilized immunohistochemistry status for estrogen, progesterone and Her2 where available, and labeled ER+, Her2+ and ER+/Her2+ (double positive) and triple negative breast cancer (TNBC; defined as ER-, PR- and Her2-).

For each subtype and all breast cancer samples, we calculated the relative enrichments of M138-specific markers in individual bulk samples by Gene Set Variation Analysis (GSVA)[39] R package (v1.44.1) implemented in R (v4.2.0). We calculated single-sample Gene Set Enrichment Analysis

(ssGSEA) scores by “gsva()” function in GSVA R package with method=”ssgsea” parameter, and used the ssGSEA scores as the proxy for presence of the capillary ECs captured by M138 in the bulk samples (**Figure 6E, D**).

DECLARATIONS

Ethics approval and consent to participate

Not applicable

Consent for publication

Not applicable

Availability of data and materials

All of the raw and processed single-cell and bulk RNA sequencing data utilized in this study are available on Synapse with Synapse project ID, the project Synapse ID, syn52966803 (DOI: https://doi.org/10.7303/syn52966803). Each folder under the project is assigned a unique Synapse ID as follows.

- **10x 8k PBMC benchmark data**: The raw and processed count matrix is available on Synapse under synapse IDs: syn52967814 (raw matrix) and syn53009488 (processed Seurat and SingleCellExperiment objects).
- **scRNA-seq of PBMCs from Influenza, COVID-19 infected and healthy control samples from Lee *et al.* 2020**: The data underlying this study are available in Gene Expression Omnibus

(GEO) at https://www.ncbi.nlm.nih.gov/geo/, and can be accessed with accession number, GSE149689. The processed data are available under Synapse ID, syn53058712.

- **scRNA-seq of breast cancer single-cell atlas from Wu *et al* 2021**: The raw count matrix and cell-level meta data were downloaded from the Broad Single-Cell Portal (https://singlecell.broadinstitute.org/single_cell/study/SCP1039). The processed data are available on Synapse under Synapse ID, syn63695719.

- **Breast cancer bulk transcriptome data from TCGA and METABRIC**: The raw count matrix and the pre-processed, log-normalized data of TCGA breast cancer RNA sequencing data are available under Synapse ID, syn64621142. The pre-processed METABRIC data are also available under Synapse ID, syn64621177.

- **Code availability:** The R codes and Multi-scale clustering (MSC) R package underlying this article are available in Zenodo (DOI: https://zenodo.org/doi/10.5281/zenodo.10214485). The developmental version of MSC is available on Github (https://github.com/songlabcodes/MSC).

Competing interests

The authors declare that they have no competing interests.

Funding

Research reported in this study was supported by the National Institutes of Health (NIH) under award numbers R35GM142918, R21AI149013, R01AI170112, RF1AG074010 and U01AG046170.

Author Contributions

Conceptualization: W.M.S. and B.Z.; Methodology: W.M.S.; Data Curation: W.M.S., C.M.; Visualization: W.M.S.; Writing, Original Draft: W.M.S.; Writing, Review & Editing: W.M.S. B.Z.; Investigation: W.M.S., Supervision: W.M.S.; Funding acquisition: W.M.S., C.F., B.Z..

ABBREVIATIONS

ARI: Adjusted Rand Index; CCA: Canonical correlation analysis; CR: Coverage rate; CSN: Cell-cell similarity network; DA: Detection accuracy; EC: Endothelial cells; FACS: Fluorescence-activated cell sorting; FDR: False discovery rate; FET: Fisher's Exact Test; GSVA: Gene Set Variation Analysis; IR: Inclusion rate; kNN : k-nearest neighbor; LEN: Locally embedded network; MAD: Median absolute deviation; METABRIC: Molecular Taxonomy of Breast Cancer International Consortium; MSC: Multi-Scale Clustering; PBMC: Peripheral blood mononuclear cells; PCA: Principal component analysis; QC: Quality controls; RB modularity: Reichardt-Bornholdt modularity; RPCA: Reciprocal principal component analysis; scRNA-seq: Single-cell RNA sequencing; SNN: Shared nearest neighbor; ssGSEA: Single-sample Gene Set Enrichment Analysis; TCGA: The Cancer Genome Atlas; TNBC: Triple-negative breast cancer; tSNE: t-distributed stochastic neighbor embedding; UMAP: Uniform Manifold Approximation and Projection; UMI: Unique molecular identifier

ACKNOWLEDGEMENTS

Not applicable

FIGURES

Figure 1. MSC workflow. A. Locally embedded network (LEN) construction. (I). Cell-wise local embedding, $\boldsymbol{\omega}_i^f$ (left), is combined into the ensemble, Θ(right). **(II).** Low quality cell links are screened as outliers (marked orange, left) in the curve of cell-cell correlation coefficient (ρ) vs mutually shared gene expressions by Jaccard index (J), and redundant links with no improvements in mutual neighbor ratio, M_{nm}, after link removal (marked brown, right). The filtered links (marked in brown and orange) are discarded to obtain the final LEN. **B Iterative top-down splitting. (I)** For each split, the clustering resolution parameter, γ, is tuned to detect the first break point, γ' (marked red), in γ vs K_{in} curve. **(II).** The parent cluster (P) is compared to its child clusters (C_1 & C_2) by cluster compactness and intra-cluster connectivity improvements. **(III)** Upon termination, MSC yields a multi-scale cluster hierarchy of parents and its more compact child clusters. **C. Identification of multi-scale cell subsets and cluster markers by MSC**. Conditioned on each parent cluster (P, marked in the schematic tSNE plot on the left), the child clusters (C1, C2,…,C5) are compared amongst them to evaluate heterogeneous cell group compositions (marked by schematic pie charts) and marker genes with distinct expressions in each child cluster (illustrated by the schematic heatmap).

Figure 2. Performance evaluation of single-cell clustering methods on simulated data by multivariate Gaussian generators with various clustering structures in the correlation matrices. A-C. Heatmaps of **A.** single-layer clustering structure with intra-cluster correlation, ρ_{in}**.** **B.** Hierarchical clustering structures with two-layers (L1: the inner layer, L2: the outer layer) whose intra-cluster correlations differ by Δρ and regular cluster sizes and, **C.** Hierarchical clustering structures with two-layers and irregular cluster sizes.**D-F**. Performances on detecting single-layer clustering structure with irregular sizes (scenario I). The evaluation metrics are

inclusion rate (D), coverage rate (E) and detection accuracy (F). **G-I**. Performances on detecting regular sized clusters embedded in two-layer hierarchy (G: Inclusion Rate, H: Coverage rate, I: Detection accuracy). L1 is the inner-layer cluster with higher intra-cluster correlation than L2, and L2 is the outer-layer cluster with a lower intra-cluster correlation. The intra-correlation difference between L1 and L2 is at $\Delta\rho=0.125$. **G-I**. Performances on detecting irregular sized clusters embedded in two-layer hierarchy (**J**: Inclusion Rate, **K**: Coverage rate, **L**: Detection accuracy). The intra-correlation difference between L1 and L2 is at $\Delta\rho=0.125$.

Figure 3. Evaluation of clustering performances in golden-standard data sets. A, B. Evaluations of AdaptSplit and other single-cell clustering methods on gold standard data sets with ground-truth clusters by adjusted Rand Index (ARI, y-axis). ARI scores are shown per data set (**A**) and per method (**B**). **C-E. Inclusion rate (C), Coverage rate (D) and Detection rate (E) of golden standard clusters (y-axis) by different methods (x-axis).** Each dot is a ground-truth cluster, different colors remark different data sets.

Figure 4. Analysis of scRNA-seq of 8k PBMC cells from healthy human donor. A. tSNE plot showing major immune cell types: Different colors represent broad immune cell types, and are labeled respectively. **B. tSNE plot showing the immune subsets**: The immune subsets are annotated into different colors with respective labels. **C-H. Clustering results from various methods**: Including AdaptSplit results from MSC (C), the clustering results are shown as different colors per panel. **I**. **Detection of major immune cell types,** evaluated by inclusion rate (Top), coverage rate (middle) and detection accuracy (bottom). J. **Detection of immune cell subtypes,** evaluated by inclusion rate (Top), coverage rate (middle) and detection accuracy

(bottom). **K**. Number of detected immune subsets by different methods (y-axis) and detection accuracy threaholds (x-axis).

Figure 5. Application of MSC to scRNA-seq of PBMC from influenza infected, COVID-19 infected and healthy control samples. A, B. UMAP plots showing the first split clusters by MSC (in **A**) and inferred cell types (in **B**)**.** The cell type colors are specified in the legend in **C**. **C, D. MSC cluster hierarchy plots:** Each node shows inferred cell type composition (in **C**) or sample compositions (in **D**). **E**. **Performance evaluation of MSC and SNN-based clustering at different resolutions**. Top: Inclusion rate, Middle: Coverage rate, Bottom: Detection accuracy. **F-J**. Sunburst plots showing MSC cluster branches enriched for asymptomatic COVID-19 patients (in **F**), healthy controls (in **G**), influenza patients (in **H**), mild COVID-19 patients (in **I**) and severe COVID-19 patients (in **J**)

Figure 6. Unsupervised multi-scale clustering of breast cancer single-cell transcriptome atlas from Wu *et al.* 2021[35]. A. UMAP plots to show major cell types (top left), minor cell types (top middle), first layer clustering by MSC (top right), SNN-based Louvain clustering at γ=0.4 (bottom left), 0.8 (bottom middle) and 1.2 (bottom right). **B.** Number of detected cell types at different resolutions (left: major cell types, middle: minor cell types, right: cell subsets by supervised subclustering) by unsupervised clustering approaches (y-axis) at different detection accuracy thresholds (x-axis). **C.** Hierarchy of cell clusters and subsets identified by MSC. Each piechart shows major cell type composition of individual cluster, as annotated by Wu *et al.* 2021, and the central piechart summarizes the overall major cell type composition in the whole data set. MSC-unique clusters showing Jaccard Index < 10% with the annotated cell types and

subsets, and clusters by SNN-based Louvain clustering at different resolutions are labeled with red. **D**. MSC identifies M138 as a unique endothelial subset (UMAP on left), compared to the annotated subsets by Wu *et al.* 2021 (UMAP on right). **E.** Dotplot of M138-specific marker genes in endothelial cells. **F.** Composition of breast cancer subtypes by ER, Her2 or triple-negative breast cancer (TNBC) status in the whole endothelial cells (left) and M138 (right). **G.** Kaplan-Meier plots of METABRIC breast cancer patients of different subtypes (left: ER+, middle: TNBC, right: the whole METABRIC cohort) stratified by the median ssGSEA score of M138-specific markers in individual transcriptome samples.

TABLES

Table 1. List of golden and silver standard data sets with known clustering structures[27]

Dataset	# features	# cells	Protocol	Description
Koh	33922	531	SMARTer	9 FACS purified differentiation stages
Kumar	41930	246	SMARTer	Mouse ESC cultured in 3 different conditions
Zhengmix4eq	10434	3994	10x	Mixtures of FACS purified PBMCs
Zhengmix4uneq	11369	6498	10x	Mixtures of FACS purified PBMCs
Zhengmix8eq	10600	3994	10x	Mixtures of FACS purified PBMCs
mixology10x3cl	16208	902	10x	Mixture of 3 cancer cell lines from CellBench
mixology10x5cl	11786	3918	10x	Mixture of 5 cancer cell lines from CellBench
simMix1	3696	2500	10x-based	Simulation of 10 human cell subpopulations
simMix2	8893	3000	10x-based	Simulation of 9 mouse cell subpopulations

SUPPLEMENTARY FIGURES

Supplemental Figure 1. Scatter plot of pairwise Pearson's correlation (ρ) against the proportion of commonly expressed genes in the respective cell pairs in LEN for PBMC 8k.

Supplemental Figure 2. Scatter plot to calculate the compactness scaling parameter (α) for PBMC 8k data set. X-axis: Module sizes randomly sampled from selecting random nodes and traversing two links to identify closely connected nodes. **Y-axis**: Scaling parameters with $\boldsymbol{\upsilon(\alpha_o) = 1}$.

Supplemental Figure 3. Computational complexity analysis for different clustering methods. Different methods are labeled in different colors, and different single-cell data are labeled as different shapes as shown in the bottom legend. **A.** Plot of runtime for different clustering methods (y-axis) against single-cell transcriptome data sets with varying numbers of cells (x-axis). The axes are in log10 scales. The scaling exponents (η) for the runtimes at different numbers of cells are labeled for each method. **B**. Plot of memory (y-axis) against single-cell transcriptome data sets with varying numbers of cells (x-axis).

Supplemental Figure 4. Detection rates of individual ground-truth clusters, stratified by sizes.

Supplemental Figure 5. Detection of clusters with two-layer hierarchy (layers labeled as L1 and L2), separating different layers by Δρ=0.25. For the simulated cluster hierarchy with regular clusters, the inclusion rate (**A**), coverage rate (**B**) and accuracy rate (**C**) are shown in **A-C**. For the simulated cluster hierarchy with irregular clusters, the inclusion rate (**D**), coverage rate (**E**) and accuracy rate (**F**) are shown in **D-F**.

Supplemental Figure 6. Sparsity of LENs and SNNs for different gold standard scRNA-seq data sets. Sparsities (C_s) of LENs constructed from Pearson's correlations (LEN:Correlation), Euclidean distance (LEN:Euclidean) and SNNs are shown.

Supplemental Figure 7. Inferred cell types of Lee data set by SingleR with the Monaco collection as the reference set.

Supplemental Figure 8. Number of detected immune subsets by different methods (y-axis) and detection accuracy threaholds (x-axis) for Lee data set.

Supplemental Figure 9. UMAP plots showing marker expressions for platelet subpopulations identified by MSC. Respective gene names are shown on top of each panel, and the child clusters of the major platelet cluster M16 in **Figure 5A** are marked.

Supplemental Figure 10. UMAP plots to show M138-specific marker expressions in endothelial cells.

Supplemental Figure 11. Kaplan-Meier plots to show prognostic significance of stratifying breast cancer patients by median expressions of M138-specific markers in predicting relapse-free survival across bulk transcriptome of 7,830 samples from 55 independent studies[41]. Four markers (CA4 (also known as RP17), ATOH8, TIMP4 and TNMD) out of the 6 tested genes with significant stratification by logrank p-value < 0.05 are shown.

SUPPLEMENTARY DATA

Supplemental Data 1. A. Meta data for individual cells from PBMC 8k data set. It includes inferred cell types in column, "inferred.cell.type.broad", for major cell types from PBMC, and more specific subtypes in "inferred.cell.type.fine". **B**. Multi-scale clusters identified MSC in .GMT format. **C**. Table of MSC identified clusters. For each cluster in each row, it specifies its parent cluster, cluster compactness and size.

Supplemental Data 2. Meta data for individual cells from Lee *et al.* 2020 data set. It includes inferred cell types in column, "inferred.cell.type.broad", for major cell types from PBMC, and more specific subtypes in "inferred.cell.type.fine". **B**. Multi-scale clusters identified MSC in .GMT format. **C**. Table of MSC identified clusters. For each cluster in each row, it specifies its parent cluster, cluster compactness and size. **D**. Enrichments of individual samples in MSC clusters by Fisher's Exact Test (FET).

Supplemental Data 3. A. Meta data for single-cell transcriptome of breast cancers from Wu *et al.* 2021. **B**. Clustering results from SNN-based Louvain clustering at γ=0.4, 0.8 and 1.2. **C**. Multi-scale clusters identified MSC in .GMT format. **D**. Table of MSC identified clusters. For each cluster in each row, it specifies its parent cluster, cluster compactness and size. **E**. Jaccard index between MSC clusters and best mapped cell types, minor cell types and subsets by supervised subclustering in Wu *et al.* 2021. **F**. Jaccard index between MSC clusters and best mapped SNN-based Louvain clusters at different resolutions. **G**. Differential expression statistics of M138-specific markers. Only includes list of significant markers genes (FDR < 0.05, fold change > 1) for M138 within endothelial cells. **H**. Clinical meta data for TCGA breast cancer cohort. Last columns include ssGSEA scores within each subtype and all primary tumor samples. **I**. Clinical meta data for METABRIC breast cancer cohort. . Last columns include ssGSEA scores within each subtype and all primary tumor samples.

REFERENCES

1. Nguyen AT, Wang K, Hu G, Wang X, Miao Z, Azevedo JA, Suh E, Van Deerlin VM, Choi D, Roeder K, et al: **APOE and TREM2 regulate amyloid-responsive microglia in Alzheimer's disease.** *Acta Neuropathol* 2020, **140:**477-493.
2. Masuda T, Sankowski R, Staszewski O, Bottcher C, Amann L, Sagar, Scheiwe C, Nessler S, Kunz P, van Loo G, et al: **Spatial and temporal heterogeneity of mouse and human microglia at single-cell resolution.** *Nature* 2019, **566:**388-392.
3. Keren-Shaul H, Spinrad A, Weiner A, Matcovitch-Natan O, Dvir-Szternfeld R, Ulland TK, David E, Baruch K, Lara-Astaiso D, Toth B, et al: **A Unique Microglia Type Associated with Restricting Development of Alzheimer's Disease.** *Cell* 2017, **169:**1276-1290 e1217.
4. Jerby-Arnon L, Shah P, Cuoco MS, Rodman C, Su MJ, Melms JC, Leeson R, Kanodia A, Mei S, Lin JR, et al: **A Cancer Cell Program Promotes T Cell Exclusion and Resistance to Checkpoint Blockade.** *Cell* 2018, **175:**984-997 e924.
5. Andrews TS, Hemberg M: **Identifying cell populations with scRNASeq.** *Mol Aspects Med* 2018, **59:**114-122.
6. Pirim H, Eksioglu B, Perkins A, Yuceer C: **Clustering of High Throughput Gene Expression Data.** *Comput Oper Res* 2012, **39:**3046-3061.
7. Han S, Toker A, Liu ZQ, Ohashi PS: **Turning the Tide Against Regulatory T Cells.** *Front Oncol* 2019, **9:**279.

8. Levine JH, Simonds EF, Bendall SC, Davis KL, Amir el AD, Tadmor MD, Litvin O, Fienberg HG, Jager A, Zunder ER, et al: **Data-Driven Phenotypic Dissection of AML Reveals Progenitor-like Cells that Correlate with Prognosis.** *Cell* 2015, **162:**184-197.
9. Xu C, Su Z: **Identification of cell types from single-cell transcriptomes using a novel clustering method.** *Bioinformatics* 2015, **31:**1974-1980.
10. Stuart T, Butler A, Hoffman P, Hafemeister C, Papalexi E, Mauck WM, 3rd, Hao Y, Stoeckius M, Smibert P, Satija R: **Comprehensive Integration of Single-Cell Data.** *Cell* 2019, **177:**1888-1902 e1821.
11. McCarthy DJ, Campbell KR, Lun AT, Wills QF: **Scater: pre-processing, quality control, normalization and visualization of single-cell RNA-seq data in R.** *Bioinformatics* 2017, **33:**1179-1186.
12. Baran Y, Bercovich A, Sebe-Pedros A, Lubling Y, Giladi A, Chomsky E, Meir Z, Hoichman M, Lifshitz A, Tanay A: **MetaCell: analysis of single-cell RNA-seq data using K-nn graph partitions.** *Genome Biol* 2019, **20:**206.
13. Newman ME: **Modularity and community structure in networks.** *Proc Natl Acad Sci U S A* 2006, **103:**8577-8582.
14. Reichardt J, Bornholdt S: **Statistical mechanics of community detection.** *Phys Rev E Stat Nonlin Soft Matter Phys* 2006, **74:**016110.
15. Wang M, Song W-m, Ming C, Wang Q, Zhou X, Xu P, Krek A, Yoon Y, Ho L, Orr ME, et al: **Guidelines for bioinformatics of single-cell sequencing data analysis in Alzheimer's disease: review, recommendation, implementation and application.** *Molecular Neurodegeneration* 2022, **17:**17.
16. Kiselev VY, Andrews TS, Hemberg M: **Challenges in unsupervised clustering of single-cell RNA-seq data.** *Nat Rev Genet* 2019, **20:**273-282.
17. Zhou Y, Song WM, Andhey PS, Swain A, Levy T, Miller KR, Poliani PL, Cominelli M, Grover S, Gilfillan S, et al: **Human and mouse single-nucleus transcriptomics reveal TREM2-dependent and TREM2-independent cellular responses in Alzheimer's disease.** *Nat Med* 2020, **26:**131-142.
18. Fortunato S, Barthelemy M: **Resolution limit in community detection.** *Proc Natl Acad Sci U S A* 2007, **104:**36-41.
19. Lu X, Cross B, Szymanski BK: **Asymptotic resolution bounds of generalized modularity and multi-scale community detection.** *Information Sciences* 2020, **525:**54-66.
20. Kiselev VY, Kirschner K, Schaub MT, Andrews T, Yiu A, Chandra T, Natarajan KN, Reik W, Barahona M, Green AR, Hemberg M: **SC3: consensus clustering of single-cell RNA-seq data.** *Nat Methods* 2017, **14:**483-486.
21. Lin P, Troup M, Ho JW: **CIDR: Ultrafast and accurate clustering through imputation for single-cell RNA-seq data.** *Genome Biol* 2017, **18:**59.
22. Duo A, Robinson MD, Soneson C: **A systematic performance evaluation of clustering methods for single-cell RNA-seq data.** *F1000Res* 2018, **7:**1141.
23. Yu L, Cao Y, Yang JYH, Yang P: **Benchmarking clustering algorithms on estimating the number of cell types from single-cell RNA-sequencing data.** *Genome Biology* 2022, **23:**49.
24. Tumminello M, Aste T, Di Matteo T, Mantegna RN: **A tool for filtering information in complex systems.** *Proc Natl Acad Sci U S A* 2005, **102:**10421-10426.
25. Song W-M, Di Matteo T, Aste T: **Hierarchical information clustering by means of topologically embedded graphs.** *PloS one* 2012, **7:**e31929.
26. El Ayeb S, Hemery B, Jeanne F, Cherrier E, Charrier C: **Evaluation Metrics for Overlapping Community Detection.** In *2022 IEEE 47th Conference on Local Computer Networks (LCN)*. IEEE; 2022: 355-358.

27. Germain PL, Sonrel A, Robinson MD: **pipeComp, a general framework for the evaluation of computational pipelines, reveals performant single cell RNA-seq preprocessing tools.** *Genome Biol* 2020, **21:**227.
28. Su S, Tian L, Dong X, Hickey PF, Freytag S, Ritchie ME: **CellBench: R/Bioconductor software for comparing single-cell RNA-seq analysis methods.** *Bioinformatics* 2020, **36:**2288-2290.
29. Jain AK, Murty MN, Flynn PJ: **Data clustering: a review.** *ACM computing surveys (CSUR)* 1999, **31:**264-323.
30. Aran D, Looney AP, Liu L, Wu E, Fong V, Hsu A, Chak S, Naikawadi RP, Wolters PJ, Abate AR, et al: **Reference-based analysis of lung single-cell sequencing reveals a transitional profibrotic macrophage.** *Nat Immunol* 2019, **20:**163-172.
31. Monaco G, Lee B, Xu W, Mustafah S, Hwang YY, Carre C, Burdin N, Visan L, Ceccarelli M, Poidinger M, et al: **RNA-Seq Signatures Normalized by mRNA Abundance Allow Absolute Deconvolution of Human Immune Cell Types.** *Cell Rep* 2019, **26:**1627-1640 e1627.
32. Tailor IK, Alshehry NF, Zaidi SZ, Marei MA, Motabi IH, Alfayez M, Altaf SY: **Outcome of Myeloma Patients with COVID-19 on Active Lenalidomide-Based Therapy: Does Lenalidomide Protect From Severe COVID-19?** *Hematol Oncol Stem Cell Ther* 2023, **16:**88-90.
33. Tran T, Lavillegrand JR, Lereverend C, Esposito B, Cartier L, Montabord M, Tran-Rajau J, Diedisheim M, Gruel N, Ouguerram K, et al: **Mild dyslipidemia accelerates tumorigenesis through expansion of Ly6C(hi) monocytes and differentiation to pro-angiogenic myeloid cells.** *Nat Commun* 2022, **13:**5399.
34. Xu F, Wang G, Zhao F, Huang Y, Fan Z, Mei S, Xie Y, Wei L, Hu Y, Wang C, et al: **IFITM3 Inhibits SARS-CoV-2 Infection and Is Associated with COVID-19 Susceptibility.** *Viruses* 2022, **14**.
35. Wu SZ, Al-Eryani G, Roden DL, Junankar S, Harvey K, Andersson A, Thennavan A, Wang C, Torpy JR, Bartonicek N, et al: **A single-cell and spatially resolved atlas of human breast cancers.** *Nat Genet* 2021, **53:**1334-1347.
36. Aran D, Hu Z, Butte AJ: **xCell: digitally portraying the tissue cellular heterogeneity landscape.** *Genome Biol* 2017, **18:**220.
37. Schupp JC, Adams TS, Cosme C, Jr., Raredon MSB, Yuan Y, Omote N, Poli S, Chioccioli M, Rose KA, Manning EP, et al: **Integrated Single-Cell Atlas of Endothelial Cells of the Human Lung.** *Circulation* 2021, **144:**286-302.
38. Ghandour MS, Langley OK, Zhu XL, Waheed A, Sly WS: **Carbonic anhydrase IV on brain capillary endothelial cells: a marker associated with the blood-brain barrier.** *Proc Natl Acad Sci U S A* 1992, **89:**6823-6827.
39. Hanzelmann S, Castelo R, Guinney J: **GSVA: gene set variation analysis for microarray and RNA-seq data.** *BMC Bioinformatics* 2013, **14:**7.
40. Curtis C, Shah SP, Chin SF, Turashvili G, Rueda OM, Dunning MJ, Speed D, Lynch AG, Samarajiwa S, Yuan Y, et al: **The genomic and transcriptomic architecture of 2,000 breast tumours reveals novel subgroups.** *Nature* 2012, **486:**346-352.
41. Gyorffy B: **Survival analysis across the entire transcriptome identifies biomarkers with the highest prognostic power in breast cancer.** *Comput Struct Biotechnol J* 2021, **19:**4101-4109.
42. Fernandez CA, Moses MA: **Modulation of angiogenesis by tissue inhibitor of metalloproteinase-4.** *Biochem Biophys Res Commun* 2006, **345:**523-529.
43. Fang F, Wasserman SM, Torres-Vazquez J, Weinstein B, Cao F, Li Z, Wilson KD, Yue W, Wu JC, Xie X, Pei X: **The role of Hath6, a newly identified shear-stress-responsive transcription factor, in endothelial cell differentiation and function.** *J Cell Sci* 2014, **127:**1428-1440.
44. Charlestin V, Fulkerson D, Arias Matus CE, Walker ZT, Carthy K, Littlepage LE: **Aquaporins: New players in breast cancer progression and treatment response.** *Front Oncol* 2022, **12:**988119.

45. Ali YB, Carriere F, Verger R, Petry S, Muller G, Abousalham A: **Continuous monitoring of cholesterol oleate hydrolysis by hormone-sensitive lipase and other cholesterol esterases.** *J Lipid Res* 2005, **46:**994-1000.
46. Trudeau RJ, Trudeau RJ: *Introduction to graph theory.* New York: Dover Pub.; 1993.
47. Song WM, Zhang B: **Multiscale Embedded Gene Co-expression Network Analysis.** *PLoS Comput Biol* 2015, **11:**e1004574.
48. Song W-M, Agrawal P, Von Itter R, Fontanals-Cirera B, Wang M, Zhou X, Mahal LK, Hernando E, Zhang B: **Network models of primary melanoma microenvironments identify key melanoma regulators underlying prognosis.** *Nature communications* 2021, **12:**1-14.
49. Song WM, Lin X, Liao X, Hu D, Lin J, Sarpel U, Ye Y, Feferman Y, Labow DM, Walsh MJ: **Multiscale network analysis reveals molecular mechanisms and key regulators of the tumor microenvironment in gastric cancer.** *International journal of cancer* 2020, **146:**1268-1280.
50. Nakagawa S, Wei L, Song WM, Higashi T, Ghoshal S, Kim RS, Bian CB, Yamada S, Sun X, Venkatesh A: **Molecular liver cancer prevention in cirrhosis by organ transcriptome analysis and lysophosphatidic acid pathway inhibition.** *Cancer cell* 2016, **30:**879-890.
51. Choi H, Song W-m, Wang M, Sram RJ, Zhang B: **Benzo [a] pyrene is associated with dysregulated myelo-lymphoid hematopoiesis in asthmatic children.** *Environment international* 2019, **128:**218-232.
52. Wang M, Li A, Sekiya M, Beckmann ND, Quan X, Schrode N, Fernando MB, Yu A, Zhu L, Cao J, et al: **Transformative Network Modeling of Multi-omics Data Reveals Detailed Circuits, Key Regulators, and Potential Therapeutics for Alzheimer's Disease.** *Neuron* 2021, **109:**257-272.e214.
53. Wang Q, Zhang Y, Wang M, Song W-M, Shen Q, McKenzie A, Choi I, Zhou X, Pan P-Y, Yue Z: **The landscape of multiscale transcriptomic networks and key regulators in Parkinson's disease.** *Nature communications* 2019, **10:**1-15.
54. Wang M, Li A, Sekiya M, Beckmann ND, Quan X, Schrode N, Fernando MB, Yu A, Zhu L, Cao J: **Molecular networks and key regulators of the dysregulated neuronal system in Alzheimer's disease.** *bioRxiv* 2019**:**788323.
55. Forst CV, Zeng L, Wang Q, Zhou X, Vatansever S, Xu P, Song WM, Tu Z, Zhang B: **Multiscale network analysis identifies potential receptors for SARS-CoV-2 and reveals their tissue-specific and age-dependent expression.** *FEBS Lett* 2023, **597:**1384-1402.
56. Hicks SC, Townes FW, Teng M, Irizarry RA: **Missing data and technical variability in single-cell RNA-sequencing experiments.** *Biostatistics* 2018, **19:**562-578.
57. Cleveland WS, Devlin SJ: **Locally weighted regression: an approach to regression analysis by local fitting.** *Journal of the American statistical association* 1988, **83:**596-610.
58. Jarvis RA, Patrick EA: **Clustering Using a Similarity Measure Based on Shared Near Neighbors.** *IEEE Transactions on Computers* 1973, **C-22:**1025-1034.
59. Traag VA, Waltman L, van Eck NJ: **From Louvain to Leiden: guaranteeing well-connected communities.** *Sci Rep* 2019, **9:**5233.
60. Song WM, Di Matteo T, Aste T: **Building complex networks with Platonic solids.** *Phys Rev E Stat Nonlin Soft Matter Phys* 2012, **85:**046115.
61. Hicks SA, Strumke I, Thambawita V, Hammou M, Riegler MA, Halvorsen P, Parasa S: **On evaluation metrics for medical applications of artificial intelligence.** *Sci Rep* 2022, **12:**5979.
62. Lun AT, Bach K, Marioni JC: **Pooling across cells to normalize single-cell RNA sequencing data with many zero counts.** *Genome Biol* 2016, **17:**75.
63. Lun AT, McCarthy DJ, Marioni JC: **A step-by-step workflow for low-level analysis of single-cell RNA-seq data with Bioconductor.** *F1000Res* 2016, **5:**2122.

64. McGinnis CS, Murrow LM, Gartner ZJ: **DoubletFinder: Doublet Detection in Single-Cell RNA Sequencing Data Using Artificial Nearest Neighbors.** *Cell Syst* 2019, **8:**329-337 e324.
65. Linderman GC, Zhao J, Roulis M, Bielecki P, Flavell RA, Nadler B, Kluger Y: **Zero-preserving imputation of single-cell RNA-seq data.** *Nat Commun* 2022, **13:**192.
66. Hafemeister C, Satija R: **Normalization and variance stabilization of single-cell RNA-seq data using regularized negative binomial regression.** *Genome Biol* 2019, **20:**296.
67. Butler A, Hoffman P, Smibert P, Papalexi E, Satija R: **Integrating single-cell transcriptomic data across different conditions, technologies, and species.** *Nat Biotechnol* 2018, **36:**411-420.
68. Mabbott NA, Baillie JK, Brown H, Freeman TC, Hume DA: **An expression atlas of human primary cells: inference of gene function from coexpression networks.** *BMC Genomics* 2013, **14:**632.
69. Finak G, McDavid A, Yajima M, Deng J, Gersuk V, Shalek AK, Slichter CK, Miller HW, McElrath MJ, Prlic M, et al: **MAST: a flexible statistical framework for assessing transcriptional changes and characterizing heterogeneity in single-cell RNA sequencing data.** *Genome Biol* 2015, **16:**278.
70. Cancer Genome Atlas N: **Comprehensive molecular portraits of human breast tumours.** *Nature* 2012, **490:**61-70.
71. Robinson MD, Oshlack A: **A scaling normalization method for differential expression analysis of RNA-seq data.** *Genome Biol* 2010, **11:**R25.

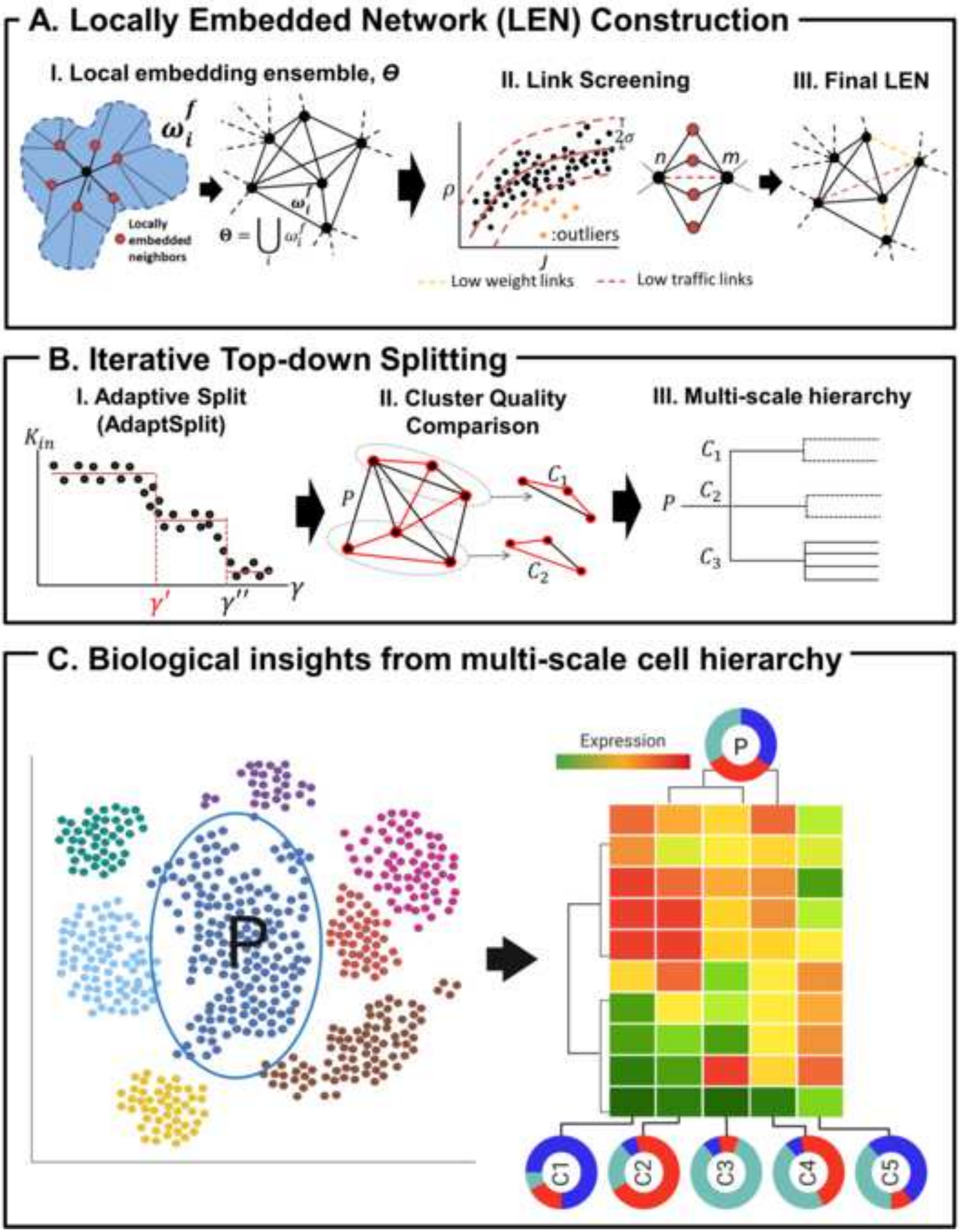
A. Locally Embedded Network (LEN) Construction
I. Local embedding ensemble, Θ
II. Link Screening
III. Final LEN
Locally embedded neighbors
:outliers
Low weight links
Low traffic links
B. Iterative Top-down Splitting
I. Adaptive Split (AdaptSplit)
II. Cluster Quality Comparison
III. Multi-scale hierarchy
C. Biological insights from multi-scale cell hierarchy
Expression
P
C1
C2
C3
C4
C5

Figure 2

Click here to access/download;Figure;Figure2.tiff

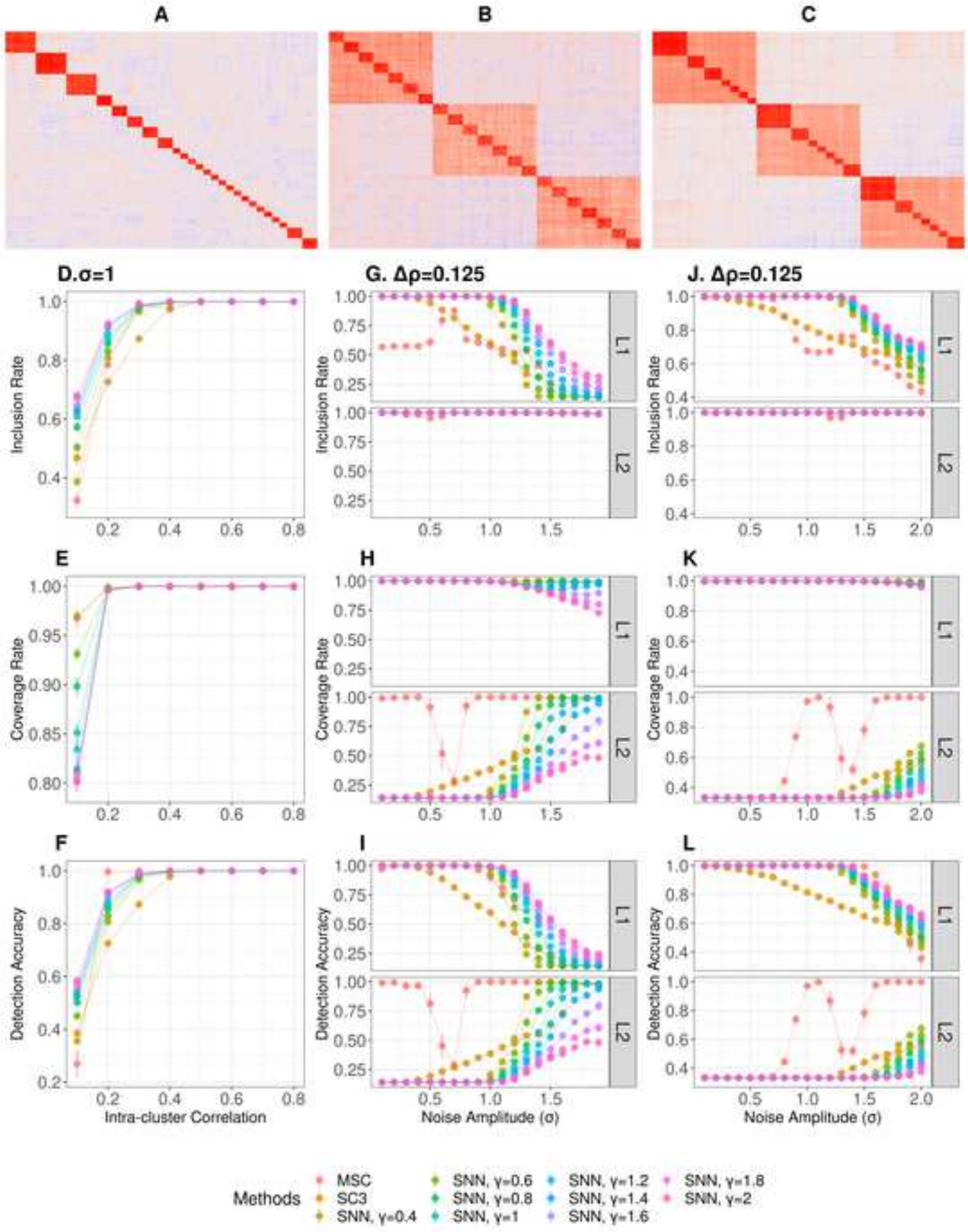

Figure 3

Click here to access/download;Figure;Figure3.tiff

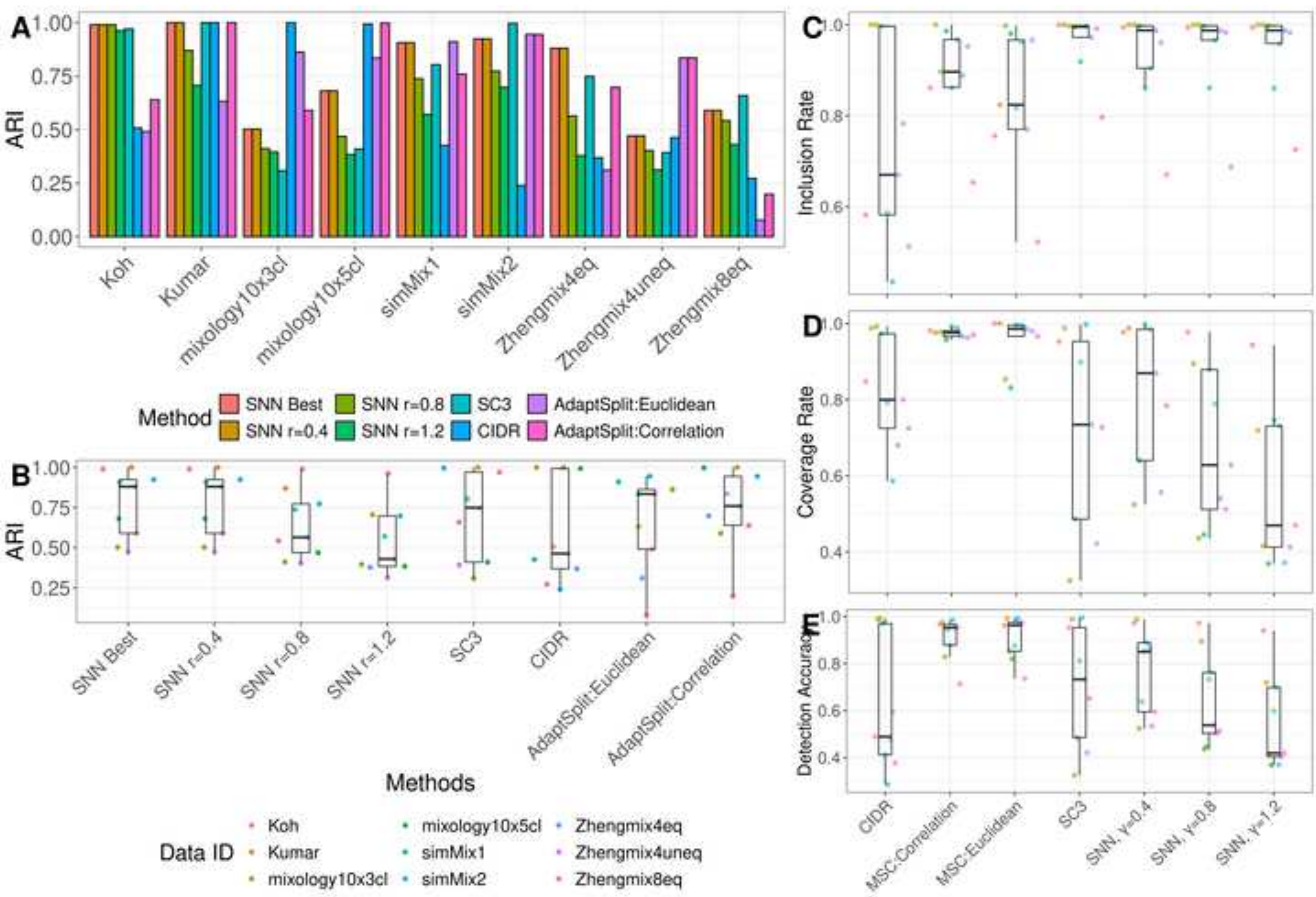

Figure 4 Click here to access/download;Figure;Figure4.tiff

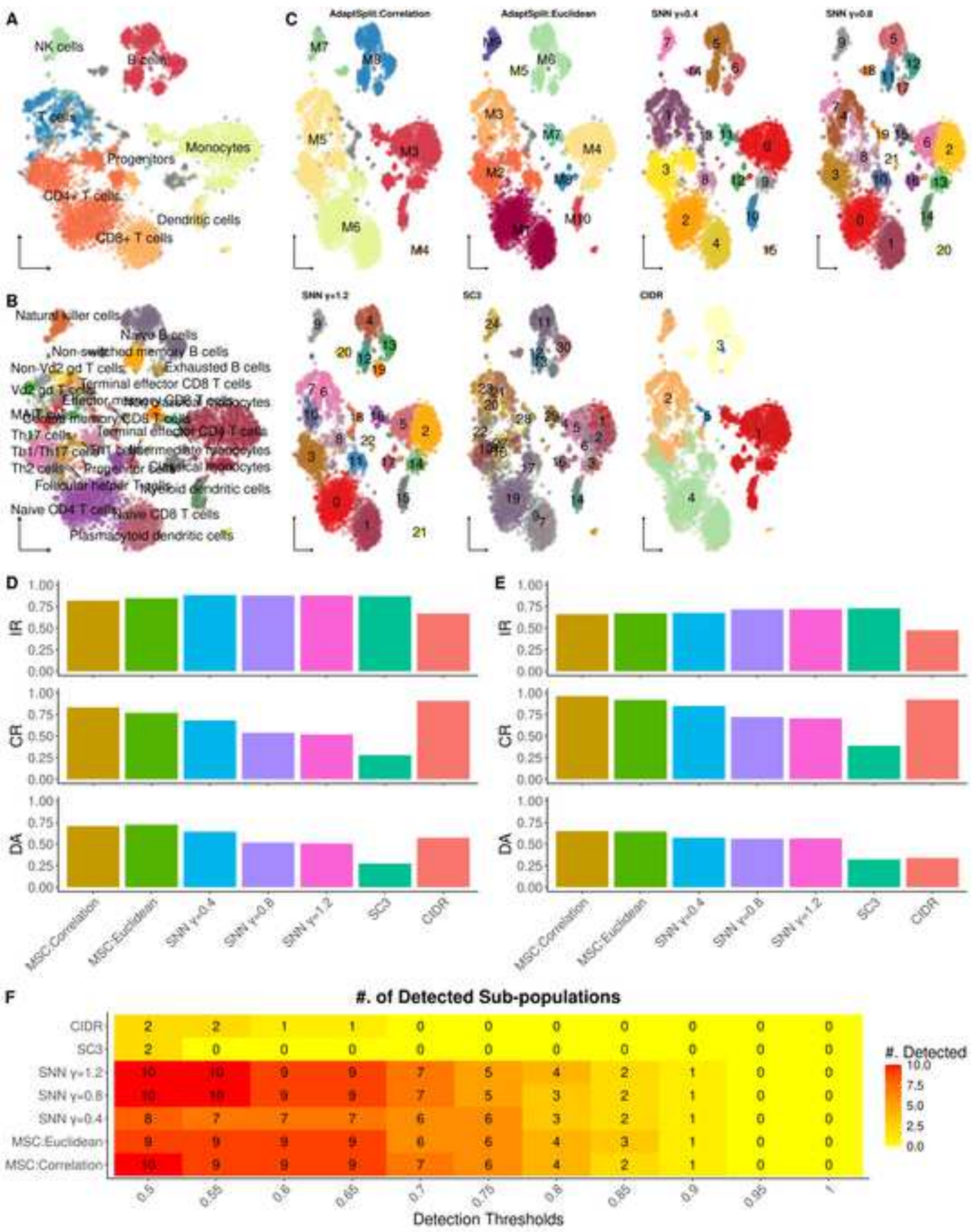

Figure 5 Click here to access/download;Figure;Figure5.tiff

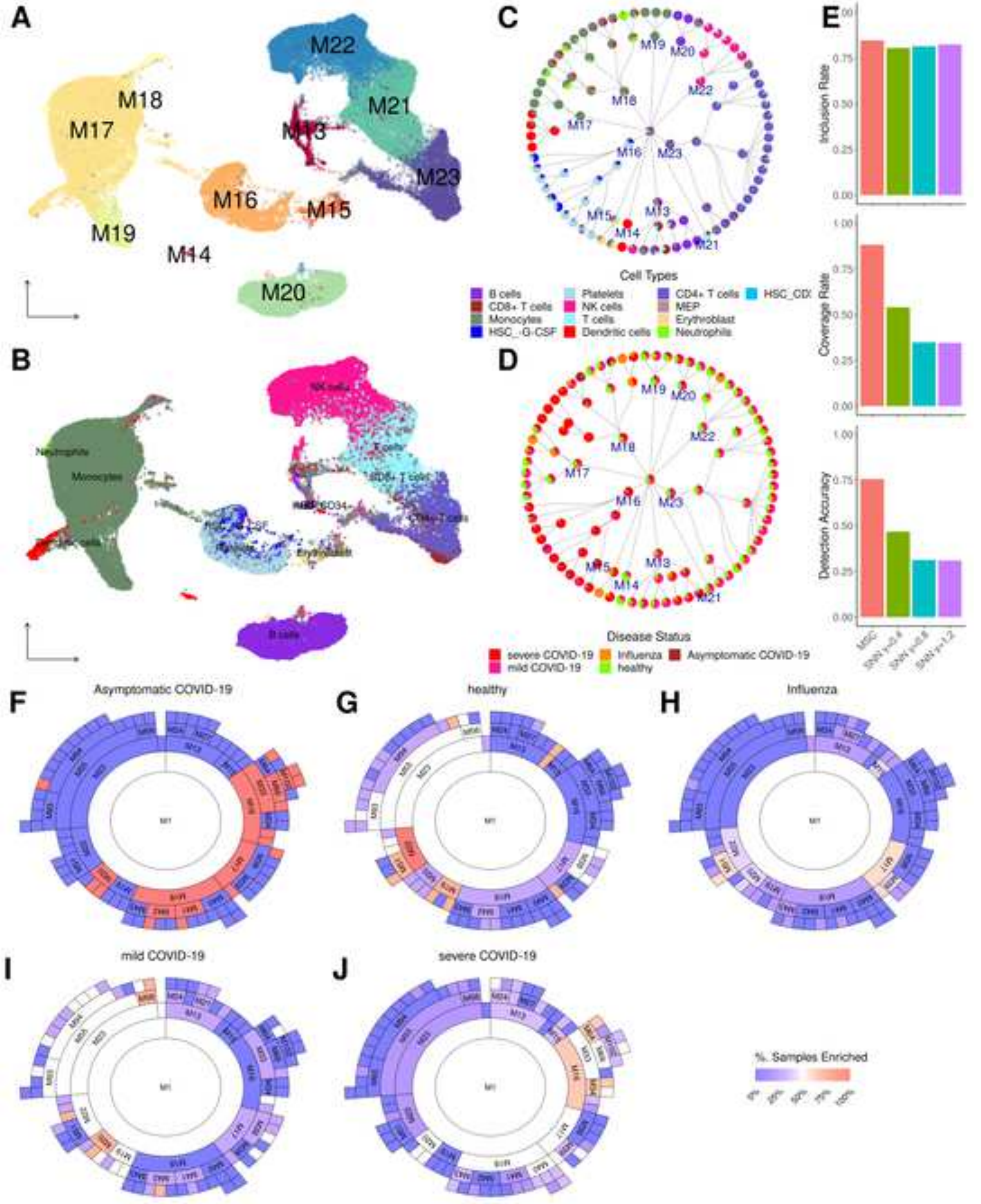

Figure 6

Click here to access/download;Figure;Figure6.tif

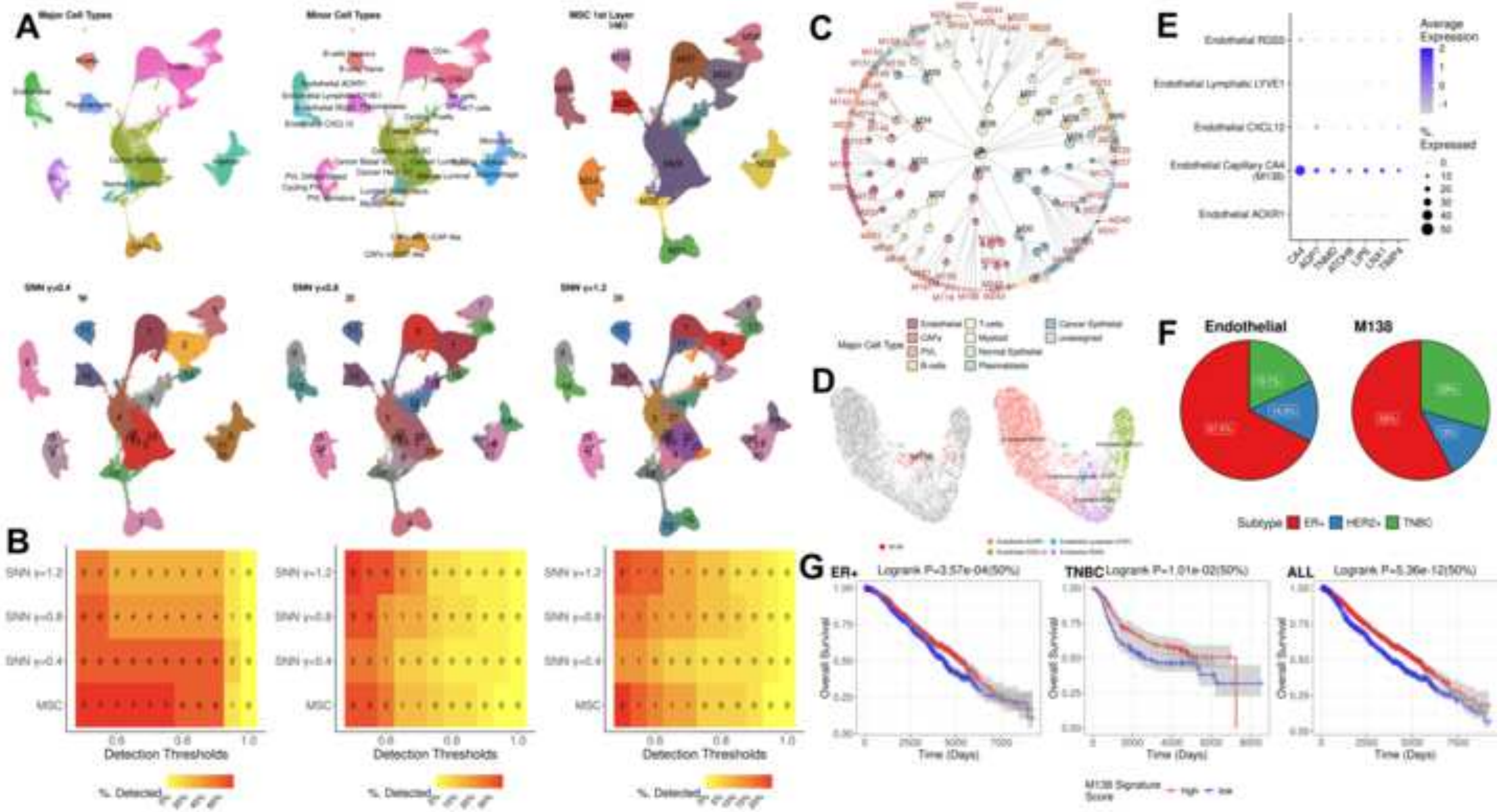

Click here to access/download
Supplementary Material
SUPPLEMENTARY MATERIAL.pdf

Supplemental Data 1

Click here to access/download

Supplementary Material

Supplementary_Data_1.xlsx

Supplemental Data 2

Click here to access/download
Supplementary Material
Supplementary_Data_2.xlsx

Supplemental Data 3

Click here to access/download
Supplementary Material
Supplementary_Data_3.xlsx

Cover Letter Click here to access/download;Personal Cover;CoverLetter.pdf

Icahn School of Medicine at Mount Sinai

Won-Min Song, PhD
One Gustave L. Levy Place, Box 1498
New York, NY 10029
T +1-212-585-6137
won-min.song@mssm.edu

January, 13th, 2025

Attn: GigaScience editor(s)

Dear editors,

Please find the manuscript titled, "**Unsupervised multi-scale clustering of single-cell transcriptomes to identify hierarchical structures of cell subtypes**" submitted by Won-Min Song, PhD, as the contact correspondence from the Icahn School of Medicine at Mount Sinai, New York, NY.

This manuscript includes a new unsupervised clustering approach to single-cell transcriptome data, multi-scale clustering (MSC). While the increasing abundance of accessible single-cell sequencing data is advancing our knowledge on cell type architectures in various tissues and conditions, discovery of novel cell types and subtypes is bottlenecked. Often, cell architectures are explored by supervised approaches guided by user bias and prior knowledge, or rare cell subtype discoveries are shadowed by inherent resolution limits in graph-theoretic clustering approaches adopted in popular toolkits such as Seurat.

To this end, MSC addresses these obstacles through a new innovative cell similarity network construction method to improve the resolution limits in cell subtype detections, and an iterative top-down clustering approach to discover *de novo* cell type hierarchy and subtypes in an unsupervised manner. We have performed comparative evaluation of MSC with existing benchmark single-cell

Won-Min Song, PhD
One Gustave L. Levy Place, Box 1498
New York, NY 10029
T +1-212-585-6137
won-min.song@mssm.edu

clustering methods (SC3, SNN based approaches, CIDR) using simulated data, golden standard data with known ground truth clusters, and silver standard data with inferred cell types. We have further shown MSC’ s capacity to detect novel cell subsets by applying it to scRNA-seq data of influenza/COVID-19 infected samples and breast cancers. These novel findings include a new platelet subtype enriched in severe COVID-19 patients, and a novel CA4-high endothelial subset in breast cancer.

To complement the results, we have also provided the developmental version of MSC in github with vignettes to reproduce the results presented in the manuscript.

Please feel free to contact me at any time if I can be of further assistance.

Sincerely,

Won-Min Song, Ph.D.
Associate Professor of Genetics and Genomic Sciences
Icahn School of Medicine at Mount Sinai
won-min.song@mssm.edu
