# Supplementary material for: Unsupervised multiscale clustering of single-cell transcriptomes to identify hierarchical structures of cell subtypes
Source: Gigascience. 2025 Oct 9;14:giaf111. doi: 10.1093/gigascience/giaf111 (PMC12509883; doi:10.1093/gigascience/giaf111)
Supplement: giaf111_GIGA-D-25-00020_Revision_2 [file giaf111_giga-d-25-00020_revision_2.pdf]

GigaScience

Unsupervised multi-scale clustering of single-cell transcriptomes to identify hierarchical structures of cell subtypes

--Manuscript Draft--

**Manuscript Number:**	GIGA-D-25-00020R2
**Full Title:**	Unsupervised multi-scale clustering of single-cell transcriptomes to identify hierarchical structures of cell subtypes
**Article Type:**	Research
**Funding Information:**	National Institute of General Medical Sciences (R35GM142918) — Associate Professor Won-Min Song<br>Division of Microbiology and Infectious Diseases, National Institute of Allergy and Infectious Diseases (R21AI149013) — Associate Professor Won-Min Song, Associate Professor Christian Forst<br>Division of Microbiology and Infectious Diseases, National Institute of Allergy and Infectious Diseases (R01AI170112) — Associate Professor Christian Forst<br>National Institute on Aging (RF1AG074010) — Professor Bin Zhang<br>National Institute on Aging (U01AG046170) — Professor Bin Zhang<br>National Institute on Aging (R01AG085182) — Professor Bin Zhang<br>U.S. Department of Defense (HT94252510001) — Professor Bin Zhang<br>Parkinson's Disease Foundation (PF-RC-936279) — Professor Bin Zhang
**Abstract:**	Background: Cell clustering is an essential step in uncovering cellular architectures in single cell RNA-sequencing (scRNA-seq) data. However, the existing cell clustering approaches are not well designed to dissect complex structures of cellular landscapes at a finer resolution.<br>Results: Here, we develop a multi-scale clustering (MSC) approach to construct sparse cell-cell correlation network for unsupervised identification of de novo cell types and subtypes across multiple resolutions. Based upon simulated, silver and gold standard data as well as real scRNA-seq data in diseases, MSC demonstrates significantly improved performance compared to established benchmark methods and reveals biologically meaningful cell hierarchy to facilitate the discovery of novel disease associated cell subtypes and mechanisms.<br>Conclusion: We present MSC as a new single-cell multi-scale clustering framework as a powerful tool for advancing discoveries in disease associated cell populations using single-cell sequencing data.
**Corresponding Author:**	Won-Min Song<br>Icahn School of Medicine at Mount Sinai<br>New York, New York UNITED STATES
**Corresponding Author Secondary Information:**	
**Corresponding Author's Institution:**	Icahn School of Medicine at Mount Sinai
**Corresponding Author's Secondary Institution:**	
**First Author:**	Won-Min Song
**First Author Secondary Information:**	
**Order of Authors:**	Won-Min Song<br>Chen Ming

Powered by Editorial Manager® and ProduXion Manager® from Aries Systems Corporation

	Christian Forst
	Bin Zhang
**Order of Authors Secondary Information:**	
**Response to Reviewers:**	The requested revision items have been addressed in the main text and the responses are detailed in the "Responses_to_editorial_comments.v1.pdf" in this revision set.
**Additional Information:**	
**Question**	**Response**
Are you submitting this manuscript to a special series or article collection?	No
**Experimental design and statistics**<br><br>Full details of the experimental design and statistical methods used should be given in the Methods section, as detailed in our Minimum Standards Reporting Checklist. Information essential to interpreting the data presented should be made available in the figure legends.<br><br>Have you included all the information requested in your manuscript?	Yes
**Resources**<br><br>A description of all resources used, including antibodies, cell lines, animals and software tools, with enough information to allow them to be uniquely identified, should be included in the Methods section. Authors are strongly encouraged to cite Research Resource Identifiers (RRIDs) for antibodies, model organisms and tools, where possible.<br><br>Have you included the information requested as detailed in our Minimum Standards Reporting Checklist?	Yes
**Availability of data and materials**<br><br>All datasets and code on which the conclusions of the paper rely must be either included in your submission or deposited in publicly available repositories	Yes

Powered by Editorial Manager® and ProduXion Manager® from Aries Systems Corporation

(where available and ethically appropriate), referencing such data using a unique identifier in the references and in the “Availability of Data and Materials” section of your manuscript.<br><br>Have you have met the above requirement as detailed in our Minimum Standards Reporting Checklist?	
GigaScience has policies and guidelines in place for the use of generative AI-writing tools such as ChatGPT. If you have used such writing tools to assist with writing the manuscript this must be declared and cited in the text. Authors should not list AI-writing tools and other AI-assisted technologies as an author or co-author and should acknowledge that they are fully responsible for text generated or refined by AI-writing tools.<p><br><br>A summary of use (particularly in the introduction or among methods) needs to be included at the end of the paper, and the outputs should also be included as a supplementary file hosted in GigaDB or other open repositories. Please read our guidelines for more information. <p><br><br>By submitting to GigaScience, you are aware of the journal's AI-writing tools policy, and if you have declared use of such tools below, you have acknowledged this where appropriate in your manuscript and have made a summary of use and outputs available. <p><br><b>AI-assisted writing tools have been used in the preparation of this manuscript?	No

Manuscript

Click here to access/download;Manuscript;manuscript_revised_v6_final.pdf

Unsupervised multi-scale clustering of single-cell transcriptomes to identify hierarchical structures of cell subtypes

Won-Min Song[1,2*§], Chen Ming[4], Christian V. Forst[1,2,3], Bin Zhang[1,2]

[1] Department of Genetics and Genomic Sciences, Icahn School of Medicine at Mount Sinai, One Gustave L. Levy Place, New York, NY 10029, USA

[2] Mount Sinai Center for Transformative Disease Modeling, Icahn School of Medicine at Mount Sinai, One Gustave L. Levy Place, New York, NY 10029, USA

[3] Department of Microbiology, Icahn School of Medicine at Mount Sinai, One Gustave L. Levy Place, New York, NY 10029, USA

[4] Faculty of Health Sciences, University of Macau, Avenida da Universidade, Taipa, Macau, China

[*] First author

[§] Corresponding author:

Won-Min Song, Ph.D.

Associate Professor, Department of Genetics & Genomic Sciences

Member, Mount Sinai Center for Transformative Disease Modeling
Icahn School of Medicine at Mount Sinai,
1399 Park Avenue, Suite 4-429, New York, NY 10029,
Tel: (332) 243-7070, Email: won-min.song@mssm.edu
Won-Min Song [0000-0003-0948-119X], Chen Ming [0000-0003-3295-301X], Bin Zhang [0000-0002-9549-5653], Christian Forst [0000-0003-0229-9459];

ABSTRACT

Background: Cell clustering is an essential step in uncovering cellular architectures in single cell RNA-sequencing (scRNA-seq) data. However, the existing cell clustering approaches are not well designed to dissect complex structures of cellular landscapes at a finer resolution.

Results: Here, we develop a multi-scale clustering (MSC) approach to construct sparse cell-cell correlation network for unsupervised identification of *de novo* cell types and subtypes across multiple resolutions. Based upon simulated, silver and gold standard data as well as real scRNA-seq data in diseases, MSC demonstrates significantly improved

performance compared to established benchmark methods and reveals biologically meaningful cell hierarchy to facilitate the discovery of novel disease associated cell subtypes and mechanisms.

Conclusion: We present MSC as a new single-cell multi-scale clustering framework as a powerful tool for advancing discoveries in disease associated cell populations using single-cell sequencing data.

Keywords: multi-scale clustering, scRNA-seq, bioinformatics, similarity network

BACKGROUND

Single-cell sequencing enables the extraction of molecular features at the cellular resolution to elucidate heterogeneous cellular landscapes in various tissues under different conditions (e.g., development and disease). Cellular heterogeneity often manifests as distinct subtypes within certain cell types, and some of these are associated with certain conditions under a study. For example, previous studies have identified expanded inflammatory monocytes in COVID-19 patients, microglia subtype associated with Alzheimer's Disease (AD)(1,2), and exclusion of cytotoxic T-cells in tumors(3). Unsupervised cell clustering analysis is crucial to capturing these heterogeneous cellular landscapes in various conditions, especially to identify novel cell populations(4,5).

Graph-theoretic approaches have been popular for understanding clustering structures in scRNA-seq to identify meaningful subpopulation architectures. These graph-theoretic approaches often utilize k-nearest neighbor (kNN) network and its variant shared nearest neighbor (SNN) networks to construct the cell similarity networks(6–8), followed by the search for closely connected subnetworks by Reichardt-Bornholdt (RB) modularity (Q_{RB}) optimization. Q_{RB} is a variant of Newman's modularity (Q_N) modularity to quantify close connections within a subnetwork, compared to randomly connected subnetworks as the

null reference(9). A unique feature of Q_{RB} is the resolution parameter (γ) to control the resolution of the optimal clustering solutions (10) and Q_{RB} is defined as,

$$Q_{RB}(\gamma) = \frac{1}{2m_o} \sum_c \left(e_c - \gamma \frac{K_c^2}{2m_o} \right)$$

where γ >0 is clustering resolution parameter, m_o is the total number of links, e_c is number of links in cluster c, K_c is the sum of degree of nodes in cluster c. By choosing various γ, it allows the natural adaptation of multi-scale detection of cell clusters(4,7,11).

However, the multi-scale cell type architectures have been primarily explored by supervised approaches, thus guided by prior knowledge and user bias. These are exemplified by user guided selection of several crucial parameters such as kNN and γ. These parameters often takedefault values such as kNN=20 and γ=1 or are determined through visual inspection of the clustering results across different parameter values via UMAP or tSNE embedding(4,11). Also, the searches for cell subtypes are often hypothesis-driven. Based on prior knowledge, supervised subclustering is performed on cell types of interest to identify subtypes at finer resolutions (1,3,12), but it could also shadow discovery for novel subtypes with little or no prior knowledge.

Further, Q_{RB} suffers from the inherent resolution limit that fundamentally restrict the detection of fine clustering structures in a network. Within a network with *m* links, the resolution limit dictates the detection of closely connected subnetworks with an internal number of links, e_c, only upto $e_c = \sqrt{2m_o}$(13), and the resolution limit persists regardless of γ(14). The dependency of resolution limit on *m* exacerbates in many kNN networks which often yield densely connected cell networks/subnetworks (i.e. $m_o \sim N_o^2$), and these could shadow rare but distinct cell subtypes present in the tissues.

Herein, we introduce an unsupervised multi-scale clustering (MSC) approach for single-cell transcriptome analysis to resolve the issues in supervised clustering approaches and the resolution limit. Within MSC, we have developed a new cell similarity network method, locally embedded network (LEN), to construct sparse and clustered cell networks and improve the sparsity-driven resolution limit in the modularity optimization problem. We have also implemented a new top-down clustering approach to iteratively split a parent network into more coherent and compact subnetworks, and eventually construct a cell hierarchy as the data-driven model of cell types and subtypes to facilitate the novel cell population discovery.

We systematically evaluated MSC's performances. Firstly, we comparatively tested LEN's performance to capture ground-truth clusters under various noise sources in scRNA-seq data. Then, we evaluated clustering performances by MSC on simulated data with hierarchical structures, golden standard data with known ground-truth clusters, and cross-platform PBMC data as silver standard data to check robust performances acrosss different sequencing platforms. Ground-truth clusters allow an objective performance comparison of MSC with widely used benchmark single-cell clustering methods such as SNN-based Louvain clustering approaches with varying γ in Seurat(7), SC3(15) and CIDR(16), which have been identified as among the best performing single-cell clustering methods(17). In addition, we have included the latest methods across different categories for comparisons, including adaptive kNN graph-based aKNNO(18), RaceID3 (designed for rare cell type identification)(19), and neural network-based scCAN(20). Then, we apply MSC to several disease scRNA-seq datasets from different tissue types to demonstrate its capacity to identify novel cell subpopulations and biological mechanisms. Overall, we present MSC as a valuable unsupervised single-cell transcriptome clustering method to understand complex cell architectures.

RESULTS

Overview of Multi-Scale Clustering (MSC) analysis framework

MSC consists of two major steps, including construction of cell similarity (also termed cell-cell interaction) network (CSN) and and top-down cell clustering on CSN (**Figure 1**). Firstly, MSC employs a novel locally embedded network (LEN) method to construct a sparse cell network without the needs to specify kNN (**Figure 1A**). For a similarity (or dissimilarity) metric of choice, LEN utilizes a graph embedding technique on topological sphere(21) to deterministically identify the nearest neighbors (NNs) for each cell. These locally embedded nearest neighbors (eNNs) are identified by searching for high similarity cell pairs among the cell and its eNNs without edge crossing when drawn on a sphere. In turn, the ensemble of eNNs for all cells constitutes the locally embedded neighbor network (LEN; **Figure 1A**-I), followed by low quality edge filtering through evaluating low similarity and edge centrality (**Figure 1A**-II, III) (see **METHODS** for details of LEN construction).

Then, MSC employs a top-down clustering approach, iteratively splitting a parent cell network into more coherent and compact subnetworks to produce a cell hierarchical

structure of cells. While different clustering solutions may emerge at different resolutions, we aim to identify the most granular clustering solution at each split, exploring cell subpopulations at progressively finer resolutions with each resolution. Specifically, we have developed *AdaptSplit*, an adaptive clustering method to search for the most granular clustering solution at each split. The child clusters from the split are compared to the parent for assessment of improvements in compactness (υ) and intra-cluster connectivity (λ) (**Figure 1B**-II; see **METHODS** for details). The iterative top-down split continues until no child cluster shows improved cluster qualities than its predecessors, completing the search for the cell hierarchy (**Figure 1B**-III). The cell hierarchy then informs data-driven biological insights into the cell subsets with distinct molecular characteristics (**Figure 1C**).

Evaluation of Locally Embedded Network (LEN) to capture cell clusters under various noises in scRNA-seq

scRNA-seq data are often noisy, and suffer from dropout reads and low library sizes to interfere with the underlying cellular landscapes(11,22). Subsequently, these noises disrupt the cell-cell connections in similarity networks and limit their capacity to capture the meaningful cell types and subtypes. Herein, we systematically evaluated the impacts of

these noises on LENs and other established benchmark similarity networks, sSNN(7) and aKNNO(18) through simulated scRNA-seq data. We utilized splatter framework(23) to generate scRNA-seq data of three clusters of sizes 50, 35 and 15 cells, across varying degrees of dropout rates and cellwise library sizes (**Figure 2A**). Specifically, *splatter* is a model-based scRNA-seq simulation framework to allow controls over expected library sizes through library size location parameter and dropout probabilities through dropout midpoints parameter (see **Methods**)(23). We varied dropout midpoints in [0,1] to adjust dropout rates, and library size locations in [5,15] to adjust the overall read depths for the simulated data.

Firstly, we evaluated the impacts of the noises on the resulting network sparsity, as the ratio of numbers of edges and nodes. Sparsity directly impacts the inherent resolution limits to detect clusters in networks(13), and we observed that LEN consistently produced the sparsest networks across all ranges of the noise parameters (**Figure 2B, C**).

In tandem, we observed that LEN consistently captured the true clusters with varying sizes across broad windows of the noise parameters. Using intra-cluster connectivity (the ratio between within-cluster and between-cluster edges) as the measure of preserving the true

clustering structures in these networks(24), we observed that aKNNOs and LENs showed comparable performances and outperformed SNNs across all parameter ranges (**Figure 2D, E**). We also observed that the smaller cluster (i.e. Group 3 in **Figure 2A**) was more severely penalized by increasing noise levels in all networks. Particularly, the impacts of library sizes were more visible than the dropout rates where library size location > 10 served as the transition point to mark the detection limits for the true clusters (**Figure 2E**).

Overall, we observed that LEN is the sparsest similarity network that can effectively capture the true clustering structures across a broad spectrum of noises in scRNA-seq. We also remark that aKNNO has been also effective to capture the true clustering structures, but at the expense of higher edge densities that are 5 — 10-folds greater than LEN.

Performance Evaluation on Simulated Data with Cluster Hierarchies

Simulated data are useful to evaluate performances of clustering methods by providing the ground-truth clusters and gain insights on how these methods behave under different scenarios by varying noises, cluster sizes and hierarchies(25). However, there are

currently no tools to simulate single-cell sequencing data with careful controls over hierarchical structures and noise parameters. To mitigate this, we utilized the multivariate Gaussian model, $X = N(\mu, \Sigma)$, with Gaussian noises, ϵ, as the stochastic data generator, $X' = X + \epsilon$. This framework allows us to instill various clustering structures including hierarchies by specifying the covariance matrix (Σ) with a higher intra-cluster covariance than the inter-cluster covariance, and have been successfully utilized in our previous study(25).

Utilizing X', we simulated stochastic data with two-layer hierarchical structure in which more correlated inner layer (L_{in}) is nested in less correlated outer layer (L_{out}) (**Figure 3A**). Two structural scenarios were considered: (I) a two-layer clustering structures with regular cluster sizes to mimic cluster hierarchy (left, **Figure 3A**) and (II) a two-layer clustering structures with irregular cluster sizes (right, **Figure 3A**). The data were simulated with varying noises amplitudes (σ) and intra-cluster correlations at different increments ($\Delta\rho = \rho_{in} - \rho_{out}$) at $\Delta\rho = 0.125$ and 0.25 as the factors shadowing the true clustering structures (see **METHODS** for details). Then, we performed MSC with Pearson's correlations across

the variable genes (MSC^{COR}) and Euclidean distances in variable PCs (MSC^{EUC}) along with other benchmark methods.

Comparing MSC^{COR} to MSC^{EUC}, MSC^{COR} outperformed MSC^{EUC} with higher cophenetic correlations and detection accuracies to identify the ground-truth hierarchies (**Figure 3B, C**). Regardless of $\Delta\rho$, one distinctive difference between the similarity metrics is low cophenetic correlations for higher noises ($\sigma \geqslant 0.75$) for the results from MSC^{EUC}, compared to the results from MSC^{COR}. We remark that this is in contrast to the outstanding performance of MSC^{EUC} over MSC^{COR} from other gold standard scRNA-seq data in the later sections (**Figure 4, 5**). Knowing that Pearson's correlation directly estimates the underlying covariance structure in the multivariate Gaussian X', we suspect that this has served beneficial to the outstanding performance of Pearson's correlation in the simulated data sets.

With the right choice of the similarity metric, we observed that MSC was able to capture the full hierarchy at different noise levels. Utilizing cophenetic correlations between MSC-inferred and ground-truth hierarchies to evaluate the concordances at individual cell levels (see **METHODS**), MSC^{COR} showed outstanding performances to detect the full hierarchy

across all noise levels at the low Δρ =0.125 and across higher noises ($\sigma \geqslant 1$) at the high Δρ = 0.25 with high cophenetic correlations over 0.9, compared to MSC^{EUC} (**Figure 3B**).

At the cluster level, MSC^{COR} outperformed the other benchmark methods in detecting the ground–truth clusters in both layers simultanesouly. We utilized detection accuracy for L_{in} and L_{out} to check the overall detection of ground–truth clusters at different layers separately. For data generated with Δρ=0.125, all clustering methods captured the full hierarchies at lower noise levels, followed by missing the detection of clusters at L_{in} at higher noise levels (bottom, **Figure 3C**). These suggest that the higher noises disrupts the ground–truth hierarchy to blend the smaller clusters at L_{in} into the larger clusters at L_{out}, and this pattern was commonly observed for all clustering methods. Nevertheless, MSC^{COR} was among the methods that captured the most clusters at L_{in} while detecting almost all clusters at L_{out} across all noise levels. These yielded the noise window, $0 \leqslant \sigma \leqslant 0.75$, that MSC^{COR} could detect the clusters at both of L_{in} and L_{out}, and MSC^{COR} was the only method that can detect the full hierarchy.

For data generated with Δρ=0.25, it revealed another unique pattern that the clustering methods only detect ground–truth clusters at L_{in} at lower noises, followed by detecting

both layers at the higher noises (top, **Figure 3C**). Of them, MSC^{COR} was among the methods that detected the most ground-truth clusters at L_{in} while managing to detect meaningful ground-truth clusters at L_{out} for $\sigma \geqslant 1.25$. We observed similar patterns of shifting noise windows to identify the ground-truth clusters at both levels by $\Delta\rho$ through evaluating the clusterability of MSC-inferred clusters with Phiclust framework(26) (see **METHODS**; **Supplemental Figure 1**). Overall, these suggest that MSC^{COR} missed the higher order structure in L_{out} at low noises when more distinctive hierarchical structure is present with larger $\Delta\rho=0.25$, and in this case, the larger noises facilitated the realization of the higher order structure. Together, we observed that MSC^{COR} could detect the clusters at both of L_{in} and L_{out} in $0.9 \leqslant \sigma \leqslant 1.2$ and MSC^{COR} was the only method to detect the full hierarchy at some noise windows.

Also, regularities of ground-truth clusters significantly affected the hierarchy detection. For most clustering methods, regular cluster sizes in L_{in} expanded the noise windows under which they are accurately detected (**Figure 1C**), compared to the irregular cluster sizes.

On the other hand, none of the resolution-based clustering with aKNNO or SNN graphs was capable of detecting both layers simultaneously. Regardless of different γ values,

these methods were not able to capture the clusters at L_{out} for $\sigma \leqslant 1$ (**Figure 1C**). Rather, higher γ imposed lower detection accuracies for clusters at L_{out} for $\sigma > 1$, and similar results were observed for $\Delta\rho$=0.125 and 0.25.

We observed similar qualitative results from a benchmark scRNA-seq of 8,381 peripheral blood mononuclear cells (PBMC) from a healthy donor from 10x website (See **Availability of data and materials**). Using the annotated cell types as a silver standard ground-truth clusters, that the multi-scale clustering results, $MSC_{ML}^{COR}$and MSC_{ML}^{EUC}, consistently captured the most similar clusters at all hierarchical levels and within different major immune types, compared to other methods (**Supplemental Figure 2, 3**). In contrast, the SNN- and aKNNO-based clustering with various resolutions emphasized detection of cell subtypes at the third level and failed to realized more granular structures in spite of the varying resolutions (See **Supplemental Results** for details).

Overall, the simulated study allowed exploring various scenarios across varying noises, cluster coherence and presence of hierarchical structures. The results demonstrate the

advantages in MSC for improved detection of clusters and hierarchy compared to benchmark methods. The simulation study also outlines several clear limitations. At certain noise windows, MSC failed to detect the hierarchical structure. When noise levels are relatively low ($\sigma \leqslant 1$), all clustering methods including MSC tend to detect the more correlated inner clusters at L_{in}. On the other hand, larger noise levels ($\sigma \leqslant 1$) tend to favor the detection of the less correlated outer cluster at L_{out}. These suggest the roles of noises in determining detectable clusters, and warrant further studies.

Cluster Compactness, *u(a),* Serves Instrumental To Probe Subclusters

We observed that cluster compactness can effectively serve to identify meaningful subcluster structures in the simulated data sets. Using Phiclust framework(26), we checked the clusterability of the parent clusters in L_{out} that, if detected, there parent clusters should yield significant Phiclust score with larger compactness compared to the child clusters in L_{in}. Testing this for simulated data from $\Delta\rho$=0.25 with regular clusters, it indeed showed that the detected parent clusters by MSC^{COR} showed larger compactness with significant clusterability (i.e. ϕ> 0.9) than the respective child clusters with unsignificant clusterability with ϕ~ 0 (**Supplemental Figure 4**).

Within MSC, we utilized the cluster compactness measure, $\upsilon(\alpha) = \overline{SPD} / log\left(N_c\right)^{\alpha}$, to determine meaningful subcluster structures, where $\overline{SPD}$ is the average of shortest path distances of all cell pairs in a network, α is the compactness scaling parameter, and N_c is the number of nodes in cluster c(27). α' values at which the parent and its child compactness coincides (i.e. $\upsilon_{parent}(\alpha') = \upsilon_{child}(\alpha')$) serve as the break points that, for α < α', the parent clusters are deemed more compact than the child clusters and, for α > α', the child clusters are more compact than the parent clusters(27). Utilizing the simulated data with different cluster sizes and hierarchies, we observed that these breakpoints varied across different cluster coherence and structures. More coherent and regular cluster sizes yielded higher α' (see **Supplemental Figure 5**; see **Supplemental Results** for details). Overall, we observed that α' reflected structural characteristics in underlying clusters.

Performance Evaluation with Gold Standard Data

We collected a number of gold standard data sets generated from independent studies, whose ground-truth clusters are known through model simulation under various scenarios, FACS-sorted cell populations and different ratio of mRNA mixtures from distinct cell lines(28,29) (**Table 1**). Using the ground-truth clusters, we sought to evaluate if the first

split by MSC can effectively distinguish the major ground-truth clusters. To this end, we identified the first split clustering by MSC using Pearson's correlations on the variable features (MSC_{1L}^{COR}) or Euclidean distances on the principal components (MSC_{1L}^{EUC}) and compared these splits to the ground truth clusters by various cluster quality metrics. We utilized adjusted Rand index (ARI)(30) and normalized mutual information (NMI)(31) measuring the similarity between the ground truth clusters and computed clusters as discrete partitions (upper panels, **Figure 4A**).

With the perfect agreements correspond to 1 in these measures, ARI and NMI showed MSC_{1L}^{EUC} and neural learning-based scCAN were the top-performing methods to capture the ground-truth partitions in these data, followed by aKNNO-based Louvain clusters at different resolutions. We have calculated entropy-based measures such as cluster purity and accuracy(32) (lower panels, **Figure 4A**) to evaluate if the computed clusters are composed of unique ground-truth clusters (i.e. purity) or if the ground-truth clusters are composed of unique computed clusters (i.e. accuracy). While the optimal clusters correspond to 0 in the entropy-based measures, we observed that MSC_{1L}^{EUC} and scCAN were the top performing methods again. We remark that MSC_{1L}^{COR} exhibited among the

best cluster purity with poor accuracy, indicative of over-clustering. Conversely, SC3, SNN and aKNNO clusters exhibited among the best accuracy with poor purity, indicative of under-clustering.

Given that MSC multi-scale clustering yields overlapping clusters, we adopted performance metrics capable of handling the overlaps (**Figure 4B**). To this end, we adopted inclusion rate (IR), equivalent to the precision measure showing correctly classified cells in an inferred cluster, coverage rate (CR), equivalent to the recall measure showing correctly classified cells in a ground-truth cluster, and detection accuracy (DA), equivalent to the accuracy measure to identify the best match between a ground-truth cluster and a inferred cluster(33) (see **METHODS** for details). Overall, the multi-scale clustering results from the Euclidean distances (MSC^{EUC}_{ML}) was among the best performing method with improved DA and CR over the first split, MSC^{EUC}_{1L} while decreased the IR. These imply that the multi-scale clustering identifies more accurate and correct clusters close to the ground-truth clusters, while the decreased IR is attributed to the increased numbers of parental clusters including members of multiple ground-truth clusters.

In contrast to the simulated data by Gaussian multivariate generator, the correlation-based MSC results, MSC^{COR}_{1L} and MSC^{COR}_{ML}, under-performed in comparison to the Euclidean-based MSC results. While the correlations were calculated across the variable genes over the cells, the Euclidean distances were calculated within the top 20 principal components from the variable genes. These imply the dimension reduction through PCA is the more effective approach to cluster the cells, and avoid negative impacts by the single-cell specific noises. On the other hand, the correlation-based results were prone to these noises.

Further, we observed LENs were consistently sparse across all gold standard data sets. The sparsity of a network can be formulated by the relationship, $m=c_sN_o$ where m is the total number of links, N_o is the number of cells, and c_s is a scaling factor to define the network sparsity. From the golden standard data sets, LENs showed $3 \leqslant c_s \leqslant 5$. On the contrary, SNN networks showed $28 \leqslant c_s \leqslant 40$, indicating LENs are substantially sparser than the SNN networks to facilitate the small yet meaningful cluster detections (**Supplemental Figure 6**).

Performance Evaluation with Silver Standard Data in PMBC Data Sets across Different Sequencing Platforms

We comparatively evaluated MSC with other single-cell clustering methods to identify meaningful cell types and subtypes from different sequencing technologies. We utilized the single-cell transcriptomes of PBMC across different sequencing platforms including 10x Chromium (v2 and v3), CEL-Seq2, Drop-seq, inDrops, Seq-Well and Smart-seq2, across technical replicates from 10x Chromium (v2) from Ding *et al.* 2020(34) (**Figure 5A, B**). We performed the clustering analyses per each platform per technical/biological replicate to test if MSC and other clustering methods can robustly detect different cell types and subtypes. Firstly, we tested the first layer split in MSC from Euclidean distances and Pearson's correlations (MSC_{1L}^{EUC},MSC_{1L}^{COR}) in comparison to the other clustering methods (**Figure 5C**).

As expected, MSC_{1L}^{EUC}andMSC_{1L}^{COR}tend to better detect the major cell types than the subtypes, and demonstrate that the first split in MSC detects the coarse-grained clustering solutions in the data across different platforms. Also, we observed slightly better

performance of MSC_{1L}^{EUC} over MSC_{1L}^{COR}. Comparing to other benchmark methods, we observed MSC_{1L}^{EUC} and MSC_{1L}^{COR} are among the best performing methods to detect the major cell types, while the cell subtype detections were sub-optimal and showed similar performances to aKNNO- or SNN-based clustering at low resolution (γ=0.4).

To evaluate the multi-scale clusters in MSC (MSC_{ML}^{EUC},MSC_{ML}^{COR}), we employed the DA, CR and IR metrics capable of handling non-overlapping clusters (**Figure 5D**). Comparing the first splits to the multi-scale clusters in MSC, multi-scale clustering improved the detection accuracy of the major cell types and subtypes in both metrics (bottom, **Figure 5D**), indicating the multi-scale search strategy succeeds in discovering more ground-truth clusters. These are also indicated in the high coverage rates from the MSC clusters (middle, **Figure 5D**), indicating that the ground-truth clusters were correctly classified into unique clusters. On the other hand, the inclusion rates were sub-optimal for MSCs to indicate the computed clusters contain different ground-truth clusters (top, **Figure 5D**). This is expected for MSCs as the coarse-grained, parent clusters in the multi-scale search inevitably include the larger clusters housing multiple ground-truth clusters.

Overall, these trends were robustly observed across different platforms and replicates for all clustering methods including MSC. These indicate that MSC can robustly detect the multi-scale cell type landscapes in different experimental and technical settings.

Applications to influenza and COVID-19 infected PBMC scRNA-seq: MSC identifies novel *CRBN/RBX1*-high platelet subpopulations in severe COVID-19

To assess the utility of MSC to study cellular landscapes in infectious diseases, we processed and analyzed single-cell transcriptome of 62,301 cells from 20 PBMC samples, comprised of 5 influenza infected patients, 11 COVID-19 infected patients with varying range of severity and 4 healthy controls from Lee *et al.* 2020(35) (see **METHODS** for data processing details).

MSC clusters systematically identified several branches of immune/blood cell types associated with influenza and COVID-19 infections. Using the finalized cell type annotations (**Figure 6B;** see **METHODS** for cell type annotations; **Supplemental Data 1A**), the MSC cluster hierarchy (**Supplemental Data 1B, C**) captured the most of the major cell types in the clusters at the first split, and the child clusters subsequently

compartmentalized into more distinct immune cell subtypes (**Figure 6A–C**), characterized by enrichments of different disease conditions (**Figure 6D**). Particularly, MSC outperformed SNN-based Louvain clustering at varying resolutions in detecting the annotated cell types and subtypes with greater IR, CR and DA (**Figure 5E**; **Supplemental Figure 11**). We note that other benchmark methods were not successfully executed due to the requirements for large computational resources by these methods, hence were omitted in the comparisons.

Several unique cell subtypes identified by MSC were associated with severe COVID-19 samples. Many cell clusters showed preferential enrichments for individuals from specific disease conditions (**Figure 5F–J**; **Supplemental Data 1D**). One example is the expansion of platelets in severe COVID-19 samples (**Figure 5J**), comprised of *CRBN/RBX1*-high (M33) and *IFITM3*-high (M34) subpopulations (**Supplemental Figure 12**). Recently, Lenalidomide, a CRBN/RBX1 inhibitor, has shown protective roles in multiple COVID-19 infected myeloma patients against progressing into severe infections(36), and suggests the emergence of this particular platelet subpopulation may drive the disease severity in COVID-19 infection. On the contrary, *IFITM3* is IFN-induced antiviral protein and its expressions are shared with monocytes/macrophages. Polymorphism in IFITM3 has been associated with COVID-

19 and severity(37), its expression inhibits COVID-19 infection(37) and these suggest M34 is a protective platelet subtype under pro-inflammatory environments. Overall, the MSC identified distinct platelet subtypes with functionally distinct characteristics, and these warrant further investigations for novel COVID-19 therapeutics.

Applications to breast cancer single-cell atlas: MSC identifies a novel protective endothelial subset in breast cancer

We expanded MSC applications to a large-scale study of breast cancer single-cell transcriptomes to explore heterogeneous tumor microenvironments and novel cell subtypes in solid tumors. Specifically, we performed MSC on single-cell transcriptome atlas of breast cancer by Wu *et al.* 2021(38), encompassing 26 breast cancer primary tumors of diverse subtypes by hormonal status (estrogen receptor (ER), progesterone receptor (PR) status), Her2 signaling status (Her2 amplification/deletion) and by molecular PAM50 subtyping(38). This study has identified major cell types and the subsets through adapting supervised approaches to infer known cell types by xCell(39) and subcluster within known major cell types by SNN-based Louvain clustering in Seurat (**Supplemental Data 2A**).

After quality controls (QC; see **METHODS** for data processing details), we processed 92,232 cells, analyzed and enumerated distinct cell populations. Firstly, we performed MSC and SNN-based clustering at varying resolutions (γ=0.4, 0.8 and 1.2) (**Figure 7A, B**), and compared the clustering results to the annotated major cell types and subsets from the published study as the silver standard ground-truth clusters (**Supplemental Data 2B-D**). We remark that many benchmark methods could not be carried out due to their excessive memory requirements. The first-split cell clusters from MSC readily captured the major cell types without supervision, while SNN-based clustering requires the fine-tuning of the resolution (**Figure 7A**). Further, MSC consistently detected higher numbers of the ground-truth clusters of major cell types and subtypes, compared to the SNN-based Louvain clustering (**Figure 7B**).

As the cell types and subtypes identified by Wu *et al.* 2021 are primarily by supervised approaches(38), we anticipated that unsupervised clustering results by MSC could potentially identify novel cell subtypes which were overlooked in the supervised approaches, and provide insights to the breast cancer biology. To this end, we leveraged the Jaccard index (JI) as a normalized overlap metric to assess MSC-unique clusters with

low overlaps against the annotated cell types/subsets, and the SNN-based Louvain clusters at different resolutions with JI < 10% (**Supplemental Data 2E**, **F**) . These yielded a large number of MSC-unique clusters, primarily as subtypes within major cell types in the cell hierarchy(**Figure 7C**).

Among these, M138 captured a unique endothelial subset that was overlooked in the previous study (**Figure 6D**). While the previous study identified the subsets characterized by ACKR1, LYVE1, CXCL12 and RGS5 (right, **Figure 6D**), M138 is a unique subset of capillary endothelial cells (ECs) characterized CA4 expressions (**Figure 7E**)(40,41), and is present in ER+, Her2+ and triple-negative breast cancer (TNBC) subtypes with enrichment of cells from TNBC, compared to the pool of all ECs (**Figure 7F;** FET p-value = 8.71E-5, EFC = 1.62).

We observed that presence of M138 EC subset in breast cancers is robustly predictive of good prognosis. To estimate the relative abundance of M138 EC subset, we identified M138-specific marker expressions (**Figure 7E**; **Supplemental Figure 13**; see **Methods** for marker identification), and performed single-sample Gene Set Enrichment Analysis (ssGSEA) score(42) as the proxy for the relative abundances of M138 ECs in METABRIC

bulk transcriptome cohort(43) (see **Methods** for METABRIC data processing). Stratifying patients by median M138 ssGSEA scores, stronger enrichments of M138 cells were significantly associated with good prognosis in ER+, TNBC and all METABRIC cohort with logrank p-value < 0.05 (**Figure 7G**). We also observed higher expressions of several M138 marker genes were significantly associated to better relapse-free survival in independent breast cancer transcriptomes from previously published studies(44)(**Supplemental Figure 14**). Reported functions of the marker genes in the literature are also supportive of the protective roles of the capillary ECs against breast cancer. These include TIMP4 (an inhibitor of capillary EC invasion(45)), TNMD (an angiogenesis inhibitor), ATOH8 (transcription factor to regulate endothelial cell proliferation(46)), AQP7(47) and LIPE(48) (regulators of fatty acid metabolism).

Overall, these results demonstrate that MSC can effectively facilitate the discovery of novel cell subsets in exploratory studies, as exemplified by M138. M138 signifies a unique capillary endothelial subset characterized by CA4 over-expressions, and its presence is robustly predictive of good prognosis in breast cancer.

Computational complexity of MSC

We analyzed the overall computational complexity, $O(n) \sim n^{\eta}$ (η is the scaling factor), of different methods through measuring the runtimes of MSC and the benchmark methods scales across data with varying sizes (n). We curated a set of publicly available scRNA-seq data whose sizes vary from small sized cohorts (< 10,000 cells) to atlas-sized cohorts (> 100,000 cells). We utilized parallel computations with 8 cores for methods with available parallel functionalities (SC3 and MSC), and assigned 8GB of memory per each core. Overall, MSC is a scalable clustering method to analyze from small to atlas-sized single-cell cohorts with feasible computational resources on personal machines. MSC and SNN-based clustering were among the most scalable methods showing $\eta \sim 1.3$, while SC3 showed $\eta \sim 2$ and CIDR showed $\eta \sim 2.7$ (**Supplemental Figure 5A**).

The memory usage was also a crucial factor for applicability. While memory usages by MSC and SNN-based clustering scaled similarly across different data sets with tractable < 50GB usages, CIDR and SC3 failed to perform due to excessive memory usage for 10,000 > cells (**Supplemental Figure 5B**). With access to high performance computing, MSC can be further parallelized to improve the overall runtime (see **Supplemental Results** for detailed analysis).

DISCUSSION

In this study, we have developed a new multi-scale cell clustering (MSC) approach. Firstly, we introduced a novel method for constructing cell similarity network, named LEN. LEN is a deterministic method that does not require user-defined parameters such as kNN and guarantees the generation of sparse cell networks owing to the utilization of embedding the nearest neighbors on a topological sphere, which imposes a hard upper bound on the number of links in the locally embedded network, m_{local}, by Euler's relation, where $m_{local} \leqslant 3(N_{local}-2)$ for such embedded networks(21). This upper bound implies the local sparsity (c_s^{local}) is restricted upto 3, and this translated to the global sparsity in $3 \leqslant c_s \leqslant 5$.

Such sparsity can inherently improve the cluster detection resolution limit via lowering the overall number of links (m_o), restricting the detection of cell clusters with the number of internal links, $e_c = \sqrt{2m_o}$(13).

We also introduced a new multi-scale clustering (MSC) algorithm, which detects meaningful cell cluster hierarchy in a LEN, and improves detection accuracy of the underlying clustering structures in the single-cell transcriptome data. The performance of

MSC was evaluated in simulated data by multivariate Gaussian models with noises. Overall, MSC outperformed other benchmark single-cell clustering methods by detecting the true clusters with greater accuracy under various scenarios simulating presence of cluster hierarchy, varying noise amplitudes, and irregular cluster sizes (**Figure 3**).

Interestingly, MSC was the only method capable of simultaneously detecting clusters at different hierarchical layers (**Figure 3B, C**). The top-down iterative clustering approach allowed detection of the nested, inner layer clusters at L_{in} after successfully detecting the outer layer clusters at L_{out}. However, depending on the cluster size regularity, different windows of noise amplitudes allowed the simultaneous detection of clusters at both layers. This is in contrast to the kNN-based clustering results detecting only one layer of clusters, regardless of the varying cluster resolution parameter, γ. Rather, the noise amplitudes were the main determinants of the kNN-based clustering results. The lower noise amplitudes favored detection of the inner layer clusters at L_{in}, and higher noise amplitudes favored the outer layer clusters at L_{out}. These translated to detecting major immune cell types and subtypes in scRNA-seq of 8,381 PBMC cells, in which MSC captured the immune cell types at different hierarchy levels most accurately among the clustering

methods (**Supplemental Figure 3**). Overall, these exemplify the benefits of multi-scale cluster detection in MSC by the top-down approach, otherwise controlling for γ alone is not capable of exploring the cluster hierarchy.

Further, we showed that MSC consistently outperformed other benchmark single-cell clustering methods across different scRNA-seq platforms. MSC showed greater detection accuracy and concordances to the ground-truth clusters in gold standard benchmark data sets from FACS sorting, or mRNA mixtures from different cell lines from different scRNA-seq platforms (**Figure 4**), and PBMC scRNA-seq from different sequencing platforms (**Figure 5**).

These superior performance of MSC is evident when applied to detect cell types in real-world scRNA-seq data from various diseases and tissues. Using inferred cell types as the silver standard, MSC detected the highest number of major cell types and their subtypes in PBMC from influenza and COVID-19 infected patients (**Figure 6**) and breast cancer (**Figure 7**). We demonstrated that MSC is capable of identifying novel cell populations associated with various disease etiologies. From the PBMC of influenza and COVID-19 infected patients, MSC identified two platelet subpopulations expanded in severe COVID-19

patients, namely, *CRBN/RBX1*–high (M33) and *IFITM3*–high (M34) cells. Particularly, the over–expression of CRBN/RBX1 exemplified the potential therapeutic implication of Lenalidomide, a CRBN/RBX1 inhibitor, in severe COVID–19 patients, where CRBN/RBX1 inhibitor were reported as protective against severe COVID–19 in several myeloma patients whose standard–of–care included Lenalidomide(36).

MSC also facilitated detection of novel cell subtypes in breast cancers. While the supervised subclustering of the endothelial cells in the published study remarked four subsets characterized by *ACKR1, LYVE1, CXCL12* and *RGS5* expressions, MSC readily identified another distinct capillary EC subset characterized by CA4 expressions. Enrichment of the capillary EC subset was robustly associated with good prognosis in multiple breast cancers bulk transcriptome cohorts, and demonstrate the utility of MSC for novel cell subset discovery in diseased tissues.

CONCLUSIONS

We have presented MSC as a new single–cell multi–scale clustering framework that integrates an innovative algorithm for constructing cell–cell similarity networks with a

multi-scale clustering strategy. MSC shows superior performance over several state-of-the-art single-cell clustering methods through an objective evaluation using a broad spectrum of simulated and real-world data with ground-truth clusters. MSC is a powerful tool for advancing discoveries in disease associated cell populations using single-cell sequencing data.

METHODS

Overview of Multi-Scale Clustering (MSC)

MSC is a two-step process consisting of cell-cell similarity network construction by locally embedded network (LEN), followed by iterative top-down splits of the cell network to realize a hierarchy of parent and child clusters (**Figure 1**).

I. Locally embedded network (LEN) construction: In many complex real-world networks, the network topologies amongst a node and its immediate neighbors are often planar, such as star graphs and wheel graphs(49). Further, planarity networks are sparse networks due to the topologically enforced upper limit on the number of links, $m = 3(N - 2)$, where N = number of nodes, by the Euler's relation(49). Taken together, this implies that the

planarity constraint could be sufficient to realize the true interacting neighbors for a node and guarantee sparsity in the resulting local network. Indeed, we have translated the planarity constraint to construct gene interaction networks(27), and these networks have been validated to capture true gene interactions and facilitated discoveries of novel regulators of disease pathways such as cancers(50–52), asthma(53), neurodegenerative diseases(54–56) and infectious diseases(57). Herein, we sought to translate the utility of the planar network to effectively construct clustered and sparse cell similarity networks.

(i) Search for locally embedded neighbors for individual cells: We leveraged the planarity constraint to determine the nearest neighboring cells to construct sparse and clustered cell similarity networks. Using a cell similarity of choice, S, LEN first searches for k most similar cells (NN^i_k), $NN^i_k = \{j|S(i,j) \leq S_k(i)\}$ where $S_k(i)$ = *k*th nearest similarity from each cell, *i*. Then, a planar maximally filtered graph (PMFG) amongst the cells in NN^i_k is constructed to identify a planar graph, P^i_k, with the maximal number of links, $3(NN^i_k - 2)$, that maximize the overall similarity among the connected cells(21) (**Figure 1A**–I). As we gradually increase *k* in $\left[3, \sqrt{N_o}\right]$($N_o$ = number of cells in the data set), the neighbors immediately connected to *i* in P^i_k saturates to a plateau at *k'* to yield the finalized nearest

neighbors, $NN^{i}_{k'} = NN^{i}$ as the locally embedded neighbors. In practice, we find $k' \sim log\left(N_o\right)$ to reach the plateau. Finally, the locally embedded network of each cell, P^{i}, is realized by connecting to its embedded neighbors, NN^{i}, and the overall locally embedded network is constructed through the ensemble across all cells, $G' = U_{i} P^{i}$.

(ii) Low quality link screening: As the local embedding explores directly linked cells, i.e. the 1st order connections, the higher order network structures such as local clustering and node centralities are overlooked in the initial network, and as results, low quality links to shadow the higher structures can be introduced in G'. Further, scRNA-seq are often noisy and may result in introducing low quality cell-cell links to further shadow the network topology. To mitigate these, we have implemented link screening steps to filter out links with low similarities and low centralities:

- *Low similarity screen*: The sparsity of single-cell transcriptome is a major source of noises and is detrimental to inferring the cell clustering structure[16, 56]. To this end, we observed the single-cell transcriptome sparsity manifested into the varying number of commonly expressed genes between two cells across a broad range, and this affected the pairwise cell similarities, S_{ij}, to vary dependently on the size of commonly

expressed genes (**Supplemental Figure 8**). Thus, we modeled the relationship between the number of common genes and the cell-cell similarity with LOESS regression(58), and identified the noisy links as the outliers from the fitted curve. Specifically, we calculated the proportion of commonly expressed genes between two cells over the union of all expressed genes in both cells, J_{ij}. Then, we evaluate the relationship between J_{ij} and S_{ij} via LOESS regression to identify the sparsity-dependent similarity thresholds as the two standard deviations away from the fitted mean (left, **Figure 1A**-II).

- *Low centrality screen*: The ratio of shared nearest neighbors between two cells, M_{ij}, is a useful 2nd order centrality measure to evaluate the local clustering structures[58]. We calculate the M_{ij} for all pairs of connected cells in G', and contest the lower quantile cell pairs by the cell-cell similarity for removal. For each contested cell pair, we evaluate if removal of the cell link improves M_{ij}. If improved, the cell link is removed and this removal occurs iteratively for all contested cell pairs. The cell link removal iteratively occurs for the similarity-sorted cell links (middle, **Figure 1A**-II).

Altogether, the local embedding and link screening yields the finalized locally embedded network (LEN), G_o.

II. Iterative top-down clustering: The clustering structure in G_o is probed by iteratively splitting parent networks into several child clusters with improved cluster qualities including connectivity (i.e. coherent clusters) and compactness (i.e. tightly connected clusters). The iterative splits terminate when no further child clusters are discovered with improved cluster qualities, and eventually identify a cell hierarchy of parent and child clusters as the data-driven model of cellular architecture in the single-cell transcriptome.

Adaptive network split (*AdaptSplit*) to search for granular clustering solutions: Each split purposely searches for the most granular clusters so that the child clusters represent the immediate subtypes of its parent cell type. These granular clusters may be defined at varying resolutions, dependent on the parent network's topology. To address this, we devised *AdaptSplit* method to adaptively search for the granular clustering solution. Specifically, *AdaptSplit* first identifies clustering solutions in $\gamma' \in (0,2]$ on a parent network, $G_o(V_o,E_o)$, by Leiden's clustering(59). The range of γ' is purposely set to explore the clustering solutions around the neutral resolution, γ'=1(9,60), and include widely used $\gamma' \leq 1.2$ in single-cell clustering(7,11).

We hypothesized that a stable, granular clustering solution should maintain stable intra–cluster connectivity at low resolutions (i.e. low γ' values). To test this, we examined the overall intra–cluster connectivity, $K_{in} = \sum_{i,j\in\Theta_c} A_{ij}$ where A_{ij}=1 if *i* and *j* are connected for a clustering solution by Louvain clustering at γ',$\Psi(\gamma = \gamma') = \{\Theta_c | \Theta_c \subseteq V_o\}$ with the disjoint conditions ($\Theta_c \cap \Theta_{c'} = \emptyset, c \neq c'$ and $U_c \Theta_c = V_o$), to maintain stable values for a range of γ' values. Typically, more fragmented and smaller clusters yield smaller K_{in}, and often, stable clustering solutions manifest as stable K_{in} to across a certain range of $\gamma' \leq \gamma \leq \gamma''$, at the break points, γ' and γ'' (**Figure 1B**–I). The break points are systematically identified by logistic regression to fit step functions incorporating the discrete K_{in} values at different γ regimes with *rpart* R package (v4.1.19). The first regime, $\gamma < \gamma'$ (highlighted in **Figure 1B**–I), is identified as the stable clustering solutions with granular clusters, and the clustering solution with median resolution in the regime, γ_r, is selected as the final clustering result for *AdaptSplit*.

Comparative evaluations of child clusters to its parent clusters for cluster quality improvements: Then, the child clusters are compared to its respective parent cluster for improved cluster qualities. This comparison assumes that the split is meaningful only if it

yields more well-defined clusters than the parent cluster, and this rationale serves to determine the termination when no further improved child clusters are detected. Specifically, we utilize (I) compactness and (II) intra-cluster connectivity as the cluster quality metrics:

I Compactness comparison: We have previously developed Multi-scale Embedded Gene co-Expression Analysis (MEGENA) that utilizes an iterative top-down clustering approach on planar gene networks(27). Within MEGENA, we established a cluster compactness measure, $\upsilon(\alpha) = \overline{SPD} / log\left(N_c\right)^{\alpha}$, where $\overline{SPD}$ is the average of shortest path distances of all cell pairs in a network, α is the compactness scaling parameter, and N_c is the number of nodes in cluster c. When comparing compactness of child clusters to the parent cluster, we showed that $\upsilon(\alpha)$ can effectively identify compact child clusters, and detect biologically meaningful cluster hierarchy of parent and child clusters(27). However, its direct translation to LEN is limited as α varies in a narrow range for planar networks(27,61). To this end, we adapted the compactness measure by fine-tuning α. In MSC workflow, α serves as the scaling parameter for $\overline{SPD}$, and determines the role of cluster sizes in calculating the compactness. To identify the suitable α for a given network, we randomly sample 100 subnetworks by propagating 3-layer neighborhoods of 100 randomly chosen nodes. Standardizing $\upsilon\left(\alpha_o\right) = 1$ as the normalized compactness where α_o serves as the reference scaling parameter, we can derive the expression for the reference scaling parameter as $\alpha_o = log\left(\overline{SPD}\right) / log\left(log\left(N_c\right)\right)$. In N_c -vs- α_o plot, α_o converged towards a constant value < 2 (See **Supplemental Figure 9**) in most cases, and this convergent value was used as the compactness scaling parameter for parent-child cluster comparisons.

II Intra-cluster connectivity comparison: In addition to the compactness comparison between the parent and child clusters, we evaluated the significance of intra-cluster density among the child clusters to ensure probing for coherent clustering structures. Within each parent cluster, *p*, the intra-cluster connectivity of each child cluster, *c*, can be defined as: $\lambda_c = e^p_{cc} / e^p_c$, where e^p_c is the number of links connected to any cells in *c*, e^p_{cc} is the number of links connecting cells within cluster *c*.

We evaluated the statistical significance of λ_c by randomly permuting 10% cells across different child clusters 100 times, and calculated the permuted intra-cluster density λ'_{cc} as the random reference values to calculate the significance p-value. With the density p-value < 0.05, the child clusters were identified as significantly coherent.

scRNA-seq simulation using Splatter framework: Splatter is a model-based scRNA-seq simulation framework that allows to control various noise sources through parametrized models(23). Within this framework, the library size is modeled through log-normal distribution, $lnN(\mu_i, \sigma_i)$, where μ_i = library size location, σ_i = library size scale. We varied

μ_i in [5,15] to shift the overall dropout rates while fixing σ_i at the default value of 0.2. We have also experimented with varying dropout rates. Splatter models the dropout probability by a logistic function, $\pi_{ij} = 1\left(1 + exp\left(-k\left(ln\left(\lambda_{ij}\right) - x_0\right)\right)\right)$, where x_0 =dropout midpoint, k= dropout shape. We varied x_0 in [0,1] while fixing k=-1 to control the overall dropout rates in the simulated data. While varying the library size locations, we fixed x_0 at the default value, 0. Likewise, we fixed μ_i at the default value of 11 while varying the dropout rates. For each unique set of parameters, we generated 10 replicates to ensure the robustness of the findings.

Disease group enrichment analysis

We performed Fisher's Exact Test (FET) to evaluate enrichment of individual cell clusters in individual samples. A sample was deemed enriched for a cell cluster if the respective FDR adjusted FET p-value (FET FDR) < 0.05. Then, for each disease condition and each cell cluster, we calculated the proportion of samples showing the enrichments, and labeled cell clusters where at least 50% of samples from a respective disease condition as enriched.

Data Simulation

We generated simulated data using multivariate Gaussian model, $X \sim N(\mu, \Sigma), X \in R^N$ with $\mu = E(X)$ is the N-dimensional mean vector, and $\Sigma_{ij} = E\left((X_i - \mu_i)(X_j - \mu_j)\right)$ is the covariance between *i*th and *j*th values in X. Then, we added data Gaussian noises ($\epsilon \sim N(\mu, 0)$) to this model, hence $X' = X + \epsilon$. Throughout the simulations, we also imposed $\Sigma_{ii} = 1$ and $\mu_i = 0$ for all i to ensure the covariance becomes synonymous with the correlation, ρ.

In this formulation, we have customized the correlation matrix to impose several clustering scenarios in the simulated data.

Two scenarios include:

I A hierarchical clustering structure of regular cluster sizes (left, **Figure 3A**): We defined two layers of clustering structures by imposing different correlation strengths at different layers. Specifically, we started by defining 21 seed clusters of size 50, constituting the inner layer clustering structure (L_{in}), with an intra-cluster correlation, ρ_{in}. Then, we adjoined six seed clusters to construct the outer layer clustering structure (L_{out}), with a weaker intra-cluster correlation, ρ_{out} with $\rho_{in} > \rho_{out} > 0$. The inter-cluster coefficients were fixed at 0. We explored two different

sub–scenarios by controlling $\Delta\rho = \rho_{in} - \rho_{out}$ at 0.125 and 0.25, to simulate different definitions in the hierarchy. Having defined the hierarchical correlation matrix, we varied the amplitude of the Gaussian noises via $\sigma \in [0.1,2]$.

II A hierarchical clustering structures of irregular cluster sizes (right, **Figure 2A**): Similar to scenario I, we imposed two–layer hierarchy with ρ_{out}=0.125 and 0.25, where the seed clusters were heterogeneous in sizes at L_{in}, including 12 clusters of size 25, 6 clusters of size 50,and 3 clusters of size 100. At L_{out}, we imposed the higher layer clustering structure by merging 4 seed clusters of size 25, 2 seed clusters of size 50, and 1 seed cluster of size 100 with ρ_{out}. Similar to scenario I, we generated $\Delta\rho = 0.125, 0.25$ with varying Gaussian noise amplitudes, $\sigma \in [0.1,2]$.

For each set of parameter, we generated 10 random replicates, across 500 features. While each scenario generates data across ~1000 cells, the number of features was deliberately selected to be much smaller than the number of cells, as observed many scRNA–seq studies(4). These simulations were performed using *MASS* R package (v7.3–57).

Evaluation Metrics

As MSC yields overlapping clusters from its parent-child cluster hierarchy, we evaluated the agreements of clustering results with the true clusters by adopting the evaluation metrics for overlapping clusters. Traditionally, for a clustering results, $\Psi' = \{\Theta'_i | i = 1, \ldots, k'\}$, and a ground-truth clusters, $\Psi^o = \{\Theta^o_j | j = 1, \ldots, k^o\}$, precision and recall were used to evaluate performances of non-overlapping cluster results. Precision represents the number of correctly classified cells over the volume of a result cluster (i.e. $P\left(\Theta'_i, \Theta^o_j\right) = \left|\Theta^o_j \cap \Theta'_i\right| / \left|\Theta'_i\right|$), and recall is the number of correctly classified cells over the volume of ground-truth (i.e. $R\left(\Theta'_i, \Theta^o_j\right) = \left|\Theta^o_j \cap \Theta'_i\right| / \left|\Theta^o_j\right|$)(33). Their extensions to overlapping clusters have been proposed by El Ayeb *et al.* 2022, as inclusion rate and coverage rate, respectively(33).

Briefly, inclusion rate (IR) evaluates the embeddedness of the result clusters to the ground-truth clusters. For each result cluster, $IR\left(\Theta'_i\right) = \max_j P\left(\Theta'_i, \Theta^o_j\right)$ defines the individual IR. Then, the overall IR is defined as the weighted sum of individual IR, . $IR(\Psi') = \sum_i IR\left(\Theta'_i\right)\left|\Theta'_i\right| / \sum_i \left|\Theta'_i\right|$On the other hand, the coverage rate (CR) evaluates the

embeddedness of the ground–truth clusters, and the individual CR is $CR\left(\Theta_j^o\right) = \max_i R\left(\Theta_i', \Theta_j^o\right)$. Then, the overall CR is $CR\left(\Psi^o\right) = \sum_j CR\left(\Theta_j^o\right)\left|\Theta_j^o\right| \Big/ \sum_j \left|\Theta_j^o\right|$.

IR and CR were shown to be highly complementary, where IR is an indicator of how similar the result clusters are to the ground–truth, and CR is an indicator of how well the ground–truth clusters are represented in the result clusters(33). However, CR values are inflated when the clustering results are under–segmented, and IR values are inflated when the clustering results are over–segmented. To this end, we devised an cluster accuracy measure to handle overlapping clusters. For each results cluster and ground–truth cluster, we calculated the ratio between their intersection and union, known as Jaccard Index (JI), as $JI\left(\Theta_i', \Theta_j^o\right) = \left|\Theta_i' \cap \Theta_j^o\right| \Big/ \left|\Theta_i' \cup \Theta_j^o\right|$. JI yields $JI\left(\Theta_i', \Theta_j^o\right)$=1 if $\Theta_i' = \Theta_j^o$, and $JI\left(\Theta_i', \Theta_j^o\right) = 0$ if there is no overlap. In analogy with CR, for each ground–truth cluster, we then defined the individual detection accuracy (DA) as the ideal overlap with the clustering results, $DA\left(\Theta_j^o\right) = \max_i JI\left(\Theta_i', \Theta_j^o\right)$. Then, the overall DA is $DA\left(\Psi^o\right) = \sum_j DA\left(\Theta_j^o\right)\left|\Theta_j^o\right| \Big/ \sum_j \left|\Theta_j^o\right|$. We used IR, CR and DA jointly to evaluate the concordance between the clustering results and ground–truth clusters.

Calculating cophenetic correlations between MSC cluster and ground-truth hierarchies: We wanted to evaluate the overall concordance between the cluster hierarchy from MSC and the ground-truth hierarchy. We utilized the cophenetic distance to calculate pairwise distances amongst the elements where the cluster compactness served as the distance metric in the cluster hierarchy dendrogram. Likewise, the cophenetic distances among the ground-truth clusters were calculated using the correlation distance, $d = \sqrt{2(1-\rho)}$. The cophenetic correlations between the cluster and ground-truth hierarchies were then calculated by Spearman's correlations between the two distance matrices.

Checking ground-truth cluster detections at different hierarchy layers: To study the impacts of noises in detecting clusters at different hierarchical layers, we evaluated the overlaps between the inferred clusters and the ground-truth clusters at L_{in} and L_{out} by Jaccard index. Jaccard index measures the proportion of intersection between two sets, A and B, to its respective union by $J(A, B) = |A \cap B| / |A \cup B|$, and this can serve to measure how identical two clusters are. We applied $J > 0.8$ to identify ground-truth clusters captured in the inferred clusters. In addition, we explored clusterability of inferred clusters, a statistical measure of significant clustering structure in a group of cells, by utilizing

Phiclust framework(26). We expected that ground-truth clusters in L_{out} should be further clusterable and imposed Phiclust score, ϕ> 0.9 as the recommended threshold by Phiclust. For ground-truth clusters in Lin, we expected that they should not be further clusterable, and imposed ϕ< 0.8 as the recommended threshold by Phiclust(26). In summary, ground-truth clusters in L_{out} were deemed as detected in inferred clusters with J > 0.8 andϕ> 0.9, and ground-truth clusters in L_{in} were deemed as detected with J > 0.8 andϕ< 0.8.

Data processing for single-cell transcriptomes of gold standard data, Lee *et al.* 2020 (influenza/COVID-19 infected PBMC) and PBMC 8k data

We performed rigorous data pre-processing and quality controls on scRNA-seq using Seurat workflow(7). First, we removed low-quality cells with mitochondrial reads > 20%, median absolute deviation (MAD) > 3 and average count > 0[62, 63]. The doublets were identified by DoubletFinder(62) and removed. The dropout reads were inferred using Adaptively thresholded Low-Rank Approximation (ALRA)(63). The filtered data will then be normalized and log-transformed by SCTransform(64). Where applicable, we integrated the single-cell transcriptome across different conditions, individuals or batches by canonical correlation analysis (CCA)(65).

Then, we selected highly variable genes as the features for cell clustering by calculating gene dispersions. Using *modelGeneVar()* function from scran package(8), we calculated biological variances of individual gene expressions from the log-normalized, pre-processed data by modeling mean-variance curve as the technical variance(66). We selected genes with biological variance p-value < 0.05 as the variable features for cell clustering. The Pearson's correlation across the selected features was used to calculate the cell similarity and perform MSC. The top 20 principal components (PCs) from the selected features were used to calculate the Euclidean distances.

Cell type identification in PBMC 8k: The cell types were annotated by applying *SingleR* (v2.2.0)(67) with bulk RNA-seq of sorted immune cell populations, also known as the Monaco collection (GSE107011), as the reference transcriptome(68). The Monaco collection data was provided through *celldex* R package (v1.6.0)(67), and accessed through *MonacoImmuneData()* function.

Cell type identification in *Lee et al.* 2020: Similar to 8k PBMC data set, most of the major cell types were annotated by *SingleR* (v2.2.0) by using the Monaco collection as the reference through *MonacoImmuneData()* function in *celldex* R package (v1.6.0). However,

the Monaco collection included immune cells only, erroneously annotated many cells as progenitors, expected to be present at 1–2% in PBMC under normal circumstances and missed out on detecting platelets and red blood cells as reported in Lee *et al.* 2020(35) (**Supplemental Figure 10**). To this end, we utilized human primary cell atlas (HPCA)(69), a microarray collection of broader blood cell types, as the reference to supplement the cell type annotations (**Figure 6B**). Similar to the Monaco collection, HPCA was accessed through *HumanPrimaryCellAtlasData()* function in *celldex* R package.

Data processing and analysis for Wu *et al.* 2021 breast cancer single-cell transcriptome atlas: Wu *et al.* 2021 data included over 90,000 cells, and the several steps in data pre-processing applied in gold standard and Lee *et al.* 2020 data sets were computational prohibitive. These include dropout read imputations by ALRA, generation of integrated and normalized gene expression data by CCA, and calculation of cell similarity by Pearson's correlation across the selected features. To this end, we performed a separate data pre-processing using computational efficient reciprocal PCA (RPCA) framework in Seurat v5 workflow(7), and the Euclidean distances in RPCA-based reduced dimension (top 50 PCs) was used to perform MSC. Specifically, we performed:

Data processing and marker analysis: The raw count matrices of single-cell transcriptomes across 20 samples from Wu *et al.* 2021(38) were downloaded from the Broad Single-Cell Portal study SCP1039. We removed low-quality cells with mitochondrial reads > 20%, median absolute deviation (MAD) > 3 and average count > 0[62, 63]. The doublets were identified by DoubletFinder and removed[64]. Considering the large number of cells (~100,000 cells) and samples to perform the integration of samplewise single-cell transcriptomes, we utilized a fast implementation of CCA, reciprocal PCA (RPCA) in Seurat v5 workflow (v5.1) in R (v4.2.0) to integrate top 50 PCs across different samples to embed them into a common reduced dimension. UMAP embeddings were subsequently calculated from the RPCA integrated coordinates for further analysis. In tandem, we normalized the samplewise single-cell transcriptomes by SCTransformation approach using "*SCTransform()*" in Seurat v5, and the normalized expressions were re-corrected by synchronizing the median UMI across different samples by "*PrepSCTFindMarkers()*" in Seurat v5 workflow. The re-corrected data were utilized for calculating cluster markers by adopting MAST framework(70) in "FindMarkers()". Ribosomal, mitochondrial rates and cellwise UMI counts served as the latent variables, and markers were identified by FDR <

0.05, and requiring a greater proportion of cells in a cell cluster/group of interest to express a marker gene than the control cell groups.

M138-specific marker identification: We first compared M138 against the rest of endothelial cells (ECs) using “*FindMarkers()*” with MAST framework as implemented in Seurat v5 workflow. We applied FDR < 0.05 and required the marker genes to be expressed in at least 10% of cells in M138, and expressed in less than 5% of the rest of ECs. We then checked if M138-specific markers within ECs were also endothelial markers by comparing their expressions in other major cell types. Similarly, we required the marker genes to be expressed in at least 10% of ECs, and expressed in less than 5% of the rest of cells.

Enrichment analysis of M138-specific program in bulk samples with good prognosis**:** We downloaded the raw count matrix for 1,080 primary tumor samples of breast cancers from The Cancer Genome Atlas (TCGA) RNA-sequencing experiments[70], and performed counts per million (CPM) normalization, followed by Trimmed Mean of M-values scaling(71) and log2(x+1) transformation using edgeR R package (v3.38.1). We then adjusted for the batch variables (data generating center, date, and machine as identified in TCGA barcode)

and patients' age by generalized linear model (*glm()* in **stats** R package, v4.2.0). Similarly, we downloaded the log-normalized gene expression data of 1,974 samples from the METABRIC cohort(43), and adjusted for batch and age by generalized linear model. Then, we utilized immunohistochemistry status for estrogen, progesterone and Her2 where available, and labeled ER+, Her2+ and ER+/Her2+ (double positive) and triple negative breast cancer (TNBC; defined as ER-, PR- and Her2-).

For each subtype and all breast cancer samples, we calculated the relative enrichments of M138-specific markers in individual bulk samples by Gene Set Variation Analysis (GSVA)(42) R package (v1.44.1) implemented in R (v4.2.0). We calculated single-sample Gene Set Enrichment Analysis (ssGSEA) scores by "*gsva()*" function in GSVA R package with method="ssgsea" parameter, and used the ssGSEA scores as the proxy for presence of the capillary ECs captured by M138 in the bulk samples (**Figure 7E, D**).

Data Availability

All of the raw and processed single-cell and bulk RNA sequencing data utilized in this study are available on Synapse with Synapse project ID, the project Synapse ID,

syn52966803(72). Each folder under the project is assigned a unique Synapse ID as follows.

- **10x 8k PBMC benchmark data**: The raw and processed count matrix is available on Synapse under synapse IDs: syn52967814 (raw matrix) and syn53009488 (processed Seurat and SingleCellExperiment objects).

- **scRNA-seq of PBMCs from Influenza, COVID-19 infected and healthy control samples from Lee *et al.* 2020**: The data underlying this study are available in Gene Expression Omnibus (GEO)(73), and can be accessed with accession number, GSE149689. The processed data are available under Synapse ID, syn53058712.

- **scRNA-seq of breast cancer single-cell atlas from Wu *et al* 2021**: The raw count matrix and cell-level meta data were downloaded from the Broad Single-Cell Portal(74) under the study ID, SCP1039. The processed data are available on Synapse under Synapse ID, syn63695719.

- **Breast cancer bulk transcriptome data from TCGA and METABRIC**: The raw count matrix and the pre-processed, log-normalized data of TCGA breast cancer RNA

sequencing data are available under Synapse ID, syn64621142. The pre-processed METABRIC data are also available under Synapse ID, syn64621177.

Availability of source code and requirements

Project name: Single-cell multi-scale clustering
Project home page: https://github.com/songlabcodes/MSC
Operating system(s): Platform independent
Programming language: R
Other requirements: R 4.2.0 or higher.
License: Data files for examples are distributed under the CC0 1.0 Universal (CC0 1.0) Otherwise, the codes are distrubuted under GPL-3.0 license as creative works.
Any restrictions to use by non-academics: None.
RRID: SCR_027342
bio.tools ID: single-cell_multi-scale_clustering_msc

The R codes and Multi-scale clustering (MSC) R package underlying this article are available in Zenodo(75). A snapshot of our GitHub project is archived in Software Heritage(76), and the workflow is also available in Workflow hub(77). The developmental version of MSC is available on Github(78). All additional supporting data are available in the GigaScience repository, GigaDB [80].

ABBREVIATIONS

ARI: Adjusted Rand Index; CCA: Canonical correlation analysis; CR: Coverage rate; CSN: Cell-cell similarity network; DA: Detection accuracy; EC: Endothelial cells; FACS:

Fluorescence-activated cell sorting; FDR: False discovery rate; FET: Fisher's Exact Test; GSVA: Gene Set Variation Analysis; IR: Inclusion rate; kNN : k-nearest neighbor; LEN: Locally embedded network; MAD: Median absolute deviation; METABRIC: Molecular Taxonomy of Breast Cancer International Consortium; MSC: Multi-Scale Cell Clustering; NMI: Normalized Mutual Information; PBMC: Peripheral blood mononuclear cells; PCA: Principal component analysis; QC: Quality controls; RB modularity: Reichardt-Bornholdt modularity; RPCA: Reciprocal principal component analysis; scRNA-seq: Single-cell RNA sequencing; SNN: Shared nearest neighbor; ssGSEA: Single-sample Gene Set Enrichment Analysis; TCGA: The Cancer Genome Atlas; TNBC: Triple-negative breast cancer; tSNE: t-distributed stochastic neighbor embedding; UMAP: Uniform Manifold Approximation and Projection; UMI: Unique molecular identifier

DECLARATIONS

Ethics approval and consent to participate

Not applicable

Consent for publication

Not applicable

Competing interests

The authors declare that they have no competing interests.

Funding

Research reported in this study was supported by the National Institutes of Health (NIH) under award numbers R35GM142918, R21AI149013, R01AI170112, R01AG085182, HT94252510001, PF-RC-936279, RF1AG074010 and U01AG046170.

Author Contributions

Conceptualization: W.M.S. and B.Z.; Methodology: W.M.S.; Data Curation: W.M.S., C.M.; Visualization: W.M.S.; Writing, Original Draft: W.M.S.; Writing, Review & Editing: W.M.S., B.Z., C.V.F; Investigation: W.M.S., Supervision: W.M.S.; Funding acquisition: W.M.S., C.V.F., B.Z..

ACKNOWLEDGEMENTS

Not applicable

FIGURES

Figure 1. MSC workflow. A. Locally embedded network (LEN) construction. (I). Cell-wise local embedding, $\boldsymbol{\omega}_i^f$ (left), is combined into the ensemble, Θ(right). **(II).** Low quality cell links are screened as outliers (marked orange, left) in the curve of cell-cell correlation coefficient (ρ) vs mutually shared gene expressions by Jaccard index (J), and redundant links with no improvements in mutual neighbor ratio, M_{nm}, after link removal (marked brown, right). The filtered links (marked in brown and orange) are discarded to obtain the final LEN. **B Iterative top-down splitting. (I)** For each split, the clustering resolution parameter, γ, is tuned to detect the first break point, γ' (marked red), in γ vs K_{in} curve. **(II).** The parent cluster (P) is compared to its child clusters (C_1 & C_2) by cluster compactness and intra-cluster connectivity improvements. **(III)** Upon termination, MSC yields a multi-scale cluster hierarchy of parents and its more compact child clusters. **C. Identification of multi-scale cell subsets and cluster markers by MSC**. Conditioned on each parent cluster (P, marked in the schematic tSNE plot on the left), the child clusters (C1, C2,…,C5) are compared amongst them to evaluate heterogeneous cell group compositions (marked by schematic

pie charts) and marker genes with distinct expressions in each child cluster (illustrated by the schematic heatmap).

Figure 2. Comparative evaluation of locally embedded network (LEN) against noises in scRNA-seq. A. Principal components (PCs) plot for first two PCs for an exemplary scRNA-seq data generated by splatter**(23)** workflow. Three clusters (Group1, 2 and 3) of varying sizes have been generated to evaluate the impact of varying noises in various clusters. **B, C. Sparsity of various similarity networks** (aKNNO: red, LEN: green, SNN: blue) across varying dropout rates (B) and library sizes (C). x-axis: dropout midpoints to define the dropout rates (in B) or library size locations to define the overall cellwise library sizes (in C) in the simulated data. y-axis: Ratio of numbers of edges and nodes in each network as the measure of sparsity. **D, E.** Intra-cluster connectivity of various similarity networks for the three clusters across varying dropouts (**D**) and library sizes (**E**). The intra-cluster connectivity is defined as the ratio of the number of within-cluster edges and the number of between-cluster edges for each cluster.

Figure 3. Evaluation of hierarchy detection in simulated data sets. A. Heatmaps of correlation coefficients amongst the cells from the simulated data. These reflect the

ground–truth hierarchies for regular (left) and irregular (right) size clusters. The inner layer of coherent clusters (L_{in}) and the outer layer of less coherent clusters (L_{out}) are labeled respectively. **B**. Cophenetic distance between ground–truth hierarchy and MSC–inferred hierarchy using Pearson's correlations and Euclidean distances. **C**. Detection accuracy to identify clusters in L_{in} and L_{out} in different scenarios. Different clustering methods are marked by unique colors, and categories by shapes.

Figure 4. Evaluation of various single–cell clustering methods to detect ground–truth clusters in pipeComp data set. A. Evaluation of agreements between the discrete clusters from various methods (in x–axis) **and the ground truth clusters** (labeled in different colors, see legend below) by adjusted rand index (ARI), normalized mutual information (NMI), cluster purity and cluster accuracy. **B. Evaluation of individual clusters from different clustering methods to reproduce the ground–truth clusters** by inclusion rate, coverage rate and detection accuracy. Each dot is a ground–truth cluster, different colors remark different data sets.

Figure 5. Evaluation of clustering performances in PBMC scRNA–seq across different single–cell RNA sequencing platforms from Ding *et al.* 2020(34). A. tSNE plots of

Harmony-integrated(79) PBMC single-cell transcriptome across different sequencing technologies (10x Chromium (v2/v3), CEL-Seq2, Drop-seq, inDrops, Seq-Well and Smart-seq2) and technical replicates (10x Chromium (v2) A and B). Major cell types (left) and subtypes (right) are shown. **B. Performance evaluations of single-cell clustering methods yielding non-overlapping discrete partitions to predict major cell types and subtypes in A.** Different colors correspond to different sequencing technologies and technical replicates. **C. Performance evaluations of the single-cell clustering methods yielding overlapping and non-overlaping solutions.**

Figure 6. Application of MSC to scRNA-seq of PBMC from influenza infected, COVID-19 infected and healthy control samples. A, B. UMAP plots showing the first split clusters by MSC (in **A**) and inferred cell types (in **B**)**.** The cell type colors are specified in the legend in **C**. **C, D. MSC cluster hierarchy plots:** Each node shows inferred cell type composition (in **C**) or sample compositions (in **D**). **E**. **Performance evaluation of MSC and SNN-based clustering at different resolutions**. Top: Inclusion rate, Middle: Coverage rate, Bottom: Detection accuracy. **F-J**. Sunburst plots showing MSC cluster branches enriched for

asymptomatic COVID−19 patients (in **F**), healthy controls (in **G**), influenza patients (in **H**), mild COVID−19 patients (in **I**) and severe COVID−19 patients (in **J**)

Figure 7. Unsupervised multi−scale clustering of breast cancer single−cell transcriptome atlas from Wu *et al.* 2021. A. UMAP plots to show major cell types (top left), minor cell types (top middle), first layer clustering by MSC (top right), SNN−based Louvain clustering at $\gamma=0.4$ (bottom left), 0.8 (bottom middle) and 1.2 (bottom right). **B.** Number of detected cell types at different resolutions (left: major cell types, middle: minor cell types, right: cell subsets by supervised subclustering) by unsupervised clustering approaches (y−axis) at different detection accuracy thresholds (x−axis). **C.** Hierarchy of cell clusters and subsets identified by MSC. Each piechart shows major cell type composition of individual cluster, as annotated by Wu *et al.* 2021, and the central piechart summarizes the overall major cell type composition in the whole data set. MSC−unique clusters showing Jaccard Index < 10% with the annotated cell types and subsets, and clusters by SNN−based Louvain clustering at different resolutions are labeled with red. **D**. MSC identifies M138 as a unique endothelial subset (UMAP on left), compared to the annotated subsets by Wu *et al.* 2021 (UMAP on right). **E.** Dotplot of M138−specific marker genes in endothelial cells. **F.**

Composition of breast cancer subtypes by ER, Her2 or triple-negative breast cancer (TNBC) status in the whole endothelial cells (left) and M138 (right). **G.** Kaplan-Meier plots of METABRIC breast cancer patients of different subtypes (left: ER+, middle: TNBC, right: the whole METABRIC cohort) stratified by the median ssGSEA score of M138-specific markers in individual transcriptome samples.

TABLES

Table 1. List of golden and silver standard data sets with known clustering structures

Dataset	# features	# cells	Protocol	Description
Koh	33922	531	SMARTer	9 FACS purified differentiation stages
Kumar	41930	246	SMARTer	Mouse ESC cultured in 3 different conditions
Zhengmix4eq	10434	3994	10x	Mixtures of FACS purified PBMCs
Zhengmix4uneq	11369	6498	10x	Mixtures of FACS purified PBMCs
Zhengmix8eq	10600	3994	10x	Mixtures of FACS purified PBMCs
mixology10x3cl	16208	902	10x	Mixture of 3 cancer cell lines from CellBench
mixology10x5cl	11786	3918	10x	Mixture of 5 cancer cell lines from CellBench
simMix1	3696	2500	10x-based	Simulation of 10 human cell subpopulations
simMix2	8893	3000	10x-	Simulation of 9 mouse cell

			based	subpopulations

SUPPLEMENTARY FIGURES

Supplemental Figure 1. Proportions of detected clusters by MSC^{COR} and MSC^{EUC} at different ground-truth layers under different Δρ=0.125 and 0.25 (labeled at the top) and cluster regularities (labeled on the right). **X-axis:** Noise amplitudes by σ. **Y-axis:** %. identified ground-truth cluster within L_{in} (upper) or L_{out} (lower).

Supplemental Figure 2. tSNE plots of PBMC 8k data set. A. The major immune cell types (top) and subtypes (bottom) are annotated into different colors with respective labels. **B. The clustering results from various methods**: AdaptSplit results from Pearson's correlations and Euclidean distances are shown along with other benchmark methods.

Supplemental Figure 3. Detection accuracy of immune cell types and subtypes by different clustering methods. Clustering methods are labeled by different colors shown on the bottom right legend. **A. Tree map of the immune cell types and subtypes** present in PBMC 8k data. **B, C. Detection accuracy of the immune cell**

types and subtypes at different stages in A (**B**) and by different major cell types (**C**) by different clustering methods.

Supplemental Figure 4. Compactness differentiates parent and child clusters with distinctions in Phiclust score as a statistical measure of clusterability. Each window represents different noise levels, and each red/blue dot represents a ground–truth child/parent cluster detected in the respective MSC^{COR} results. X–axis: Compactness, Y–axis: Phiclust score.

Supplemental Figure 5. Evaluation of compactness as a function of the exponent α, $\upsilon(\alpha)$, by different hierarchical structures and similarity measures. A, B. Compactness for ground–truth parent clusters (green) and child clusters (red) in LENs computed from Pearson's correlation (**A**) and Euclidean distance (**B**) on simulated data with hierarchy among irregular sized clusters. across various α in [0,3] across 10 random replicates. The horizontal dotted lines show the transition points, α', where $\upsilon_{parent}(\alpha') = \upsilon_{child}(\alpha')$. On the far right, the boxplot of α' values is shown. **C, D**. Similar plots as **A** for LENs computed from Pearson's correlation (**C**) and Euclidean distance (**D**) on simulated data with hierarchy among regular sized clusters.

Supplemental Figure 6. Sparsity of LENs and SNNs for different gold standard scRNA-seq data sets. Sparsities (C_s) of LENs constructed from Pearson's correlations (LEN:Correlation), Euclidean distance (LEN:Euclidean) and SNNs are shown.

Supplemental Figure 7. Computational complexity analysis for different clustering methods. Different methods are labeled in different colors, and different single-cell data are labeled as different shapes as shown in the bottom legend. **A.** Plot of runtime for different clustering methods (y-axis) against single-cell transcriptome data sets with varying numbers of cells (x-axis). The axes are in log10 scales. The scaling exponents (η) for the runtimes at different numbers of cells are labeled for each method. **B**. Plot of memory (y-axis) against single-cell transcriptome data sets with varying numbers of cells (x-axis).

Supplemental Figure 8. Scatter plot of pairwise Pearson's correlation (ρ) against the proportion of commonly expressed genes in the respective cell pairs in LEN for PBMC 8k.

Supplemental Figure 9. Scatter plot to calculate the compactness scaling parameter (α) for PBMC 8k data set. X-axis: Module sizes randomly sampled from selecting random

nodes and traversing two links to identify closely connected nodes. **Y–axis**: Scaling parameters with $\boldsymbol{\upsilon}(\boldsymbol{\alpha_o}) = \mathbf{1}$.

Supplemental Figure 10. Inferred cell types of Lee data set by SingleR with the Monaco collection as the reference set.

Supplemental Figure 11. Number of detected immune subsets by different methods (y–axis) and detection accuracy threaholds (x–axis) for Lee data set.

Supplemental Figure 12. UMAP plots showing marker expressions for platelet subpopulations identified by MSC. Respective gene names are shown on top of each panel, and the child clusters of the major platelet cluster M16 in **Figure 5A** are marked.

Supplemental Figure 13. UMAP plots show M138–specific marker expressions in endothelial cells.

Supplemental Figure 14. Kaplan–Meier plots to show prognostic significance of stratifying breast cancer patients by median expressions of M138–specific markers in predicting relapse–free survival across bulk transcriptome of 7,830 samples from 55 independent

studies(44). Four markers (CA4 (also known as RP17), ATOH8, TIMP4 and TNMD) out of the 6 tested genes with significant stratification by logrank p-value < 0.05 are shown.

SUPPLEMENTAL DATA

Supplemental Data 1. Meta data for individual cells from Lee *et al.* 2020 data set. It includes inferred cell types in column, “inferred.cell.type.broad”, for major cell types from PBMC, and more specific subtypes in “inferred.cell.type.fine”. **B**. Multi-scale clusters identified MSC in .GMT format. **C**. Table of MSC identified clusters. For each cluster in each row, it specifies its parent cluster, cluster compactness and size. **D**. Enrichments of individual samples in MSC clusters by Fisher’s Exact Test (FET).

Supplemental Data 2. A. Meta data for single-cell transcriptome of breast cancers from Wu *et al.* 2021. **B**. Clustering results from SNN-based Louvain clustering at γ=0.4, 0.8 and 1.2. **C**. Multi-scale clusters identified MSC in .GMT format. **D**. Table of MSC identified clusters. For each cluster in each row, it specifies its parent cluster, cluster compactness and size. **E**. Jaccard index between MSC clusters and best mapped cell types, minor cell types and subsets by supervised subclustering in Wu *et al.* 2021. **F**. Jaccard index between

MSC clusters and best mapped SNN–based Louvain clusters at different resolutions. **G**. Differential expression statistics of M138–specific markers. Only includes a list of significant markers genes (FDR < 0.05, fold change > 1) for M138 within endothelial cells. **H**. Clinical meta data for TCGA breast cancer cohort. Last columns include ssGSEA scores within each subtype and all primary tumor samples. **I**. Clinical meta data for METABRIC breast cancer cohort. The last columns include ssGSEA scores within each subtype and all primary tumor samples.

REFERENCES

1. Keren-Shaul H, Spinrad A, Weiner A, Matcovitch-Natan O, Dvir-Szternfeld R, Ulland TK, et al. A Unique Microglia Type Associated with Restricting Development of Alzheimer’s Disease. Cell. 2017 June 15;169(7):1276-1290.e17.
2. Masuda T, Sankowski R, Staszewski O, Böttcher C, Amann L, Sagar null, et al. Spatial and temporal heterogeneity of mouse and human microglia at single-cell resolution. Nature. 2019 Feb;566(7744):388–92.
3. Jerby-Arnon L, Shah P, Cuoco MS, Rodman C, Su MJ, Melms JC, et al. A Cancer Cell Program Promotes T Cell Exclusion and Resistance to Checkpoint Blockade. Cell. 2018;175(4).
4. Kiselev VY, Andrews TS, Hemberg M. Challenges in unsupervised clustering of single-cell RNA-seq data. Nat Rev Genet. 2019 May;20(5):273–82.
5. Andrews TS, Hemberg M. Identifying cell populations with scRNASeq. Mol Aspects Med. 2018 Feb;59:114–22.
6. Levine JH, Simonds EF, Bendall SC, Davis KL, Amir E ad D, Tadmor MD, et al. Data-Driven Phenotypic Dissection of AML Reveals Progenitor-like Cells that Correlate with Prognosis. Cell. 2015 July 2;162(1):184–97.
7. Stuart T, Butler A, Hoffman P, Hafemeister C, Papalexi E, Mauck WM, et al. Comprehensive Integration of Single-Cell Data. Cell. 2019 June 13;177(7):1888-1902 e21.

8. McCarthy DJ, Campbell KR, Lun ATL, Wills QF. Scater: pre-processing, quality control, normalization and visualization of single-cell RNA-seq data in R. Bioinformatics. 2017 Apr 15;33(8):1179–86.

9. Newman ME. Modularity and community structure in networks. Proc Natl Acad Sci U S A. 2006 June 6;103(23):8577–82.

10. Reichardt J, Bornholdt S. Statistical mechanics of community detection. Phys Rev E Stat Nonlin Soft Matter Phys. 2006 July;74(1 Pt 2):016110.

11. Wang M, Song W min, Ming C, Wang Q, Zhou X, Xu P, et al. Guidelines for bioinformatics of single-cell sequencing data analysis in Alzheimer's disease: review, recommendation, implementation and application. Molecular neurodegeneration. 2022 Mar 2;17(1):17.

12. Zhou Y, Song WM, Andhey PS, Swain A, Levy T, Miller KR, et al. Human and mouse single-nucleus transcriptomics reveal TREM2-dependent and TREM2-independent cellular responses in Alzheimer's disease. Nat Med. 2020 Jan;26(1):131–42.

13. Fortunato S, Barthelemy M. Resolution limit in community detection. Proc Natl Acad Sci U S A. 2007 Jan 2;104(1):36–41.

14. Lu X, Cross B, Szymanski BK. Asymptotic resolution bounds of generalized modularity and multi-scale community detection. Information Sciences. 2020 July 1;525:54–66.

15. Kiselev VY, Kirschner K, Schaub MT, Andrews T, Yiu A, Chandra T, et al. SC3: consensus clustering of single-cell RNA-seq data. Nat Methods. 2017 May;14(5):483–6.

16. Lin P, Troup M, Ho JWK. CIDR: Ultrafast and accurate clustering through imputation for single-cell RNA-seq data. Genome Biol [Internet]. 2017 Dec [cited 2025 July 11];18(1). Available from: http://genomebiology.biomedcentral.com/articles/10.1186/s13059-017-1188-0

17. Yu L, Cao Y, Yang JYH, Yang P. Benchmarking clustering algorithms on estimating the number of cell types from single-cell RNA-sequencing data. Genome Biol. 2022 Feb 8;23(1):49.

18. Li J, Shyr Y, Liu Q. aKNNO: single-cell and spatial transcriptomics clustering with an optimized adaptive k-nearest neighbor graph. Genome Biol. 2024 Aug 1;25(1):203.

19. Rosales-Alvarez RE, Rettkowski J, Herman JS, Dumbović G, Cabezas-Wallscheid N, Grün D. VarID2 quantifies gene expression noise dynamics and unveils functional heterogeneity of ageing hematopoietic stem cells. Genome Biol. 2023 June 23;24(1):148.

20. Tran B, Tran D, Nguyen H, Ro S, Nguyen T. scCAN: single-cell clustering using autoencoder and network fusion. Sci Rep [Internet]. 2022 June 17 [cited 2025 July 11];12(1). Available from: https://www.nature.com/articles/s41598-022-14218-6

21. Tumminello M, Aste T, Di Matteo T, Mantegna RN. A tool for filtering information in complex systems. Proc Natl Acad Sci U S A. 2005 July 26;102(30):10421–6.

22. Haque A, Engel J, Teichmann SA, Lönnberg T. A practical guide to single-cell RNA-sequencing for biomedical research and clinical applications. Genome Med. 2017 Dec;9(1):75.

23. Zappia L, Phipson B, Oshlack A. Splatter: simulation of single-cell RNA sequencing data. Genome Biol. 2017 Dec;18(1):174.

24. Dong J, Horvath S. Understanding network concepts in modules. BMC Syst Biol. 2007 June 4;1:24.

25. Song WM, Di Matteo T, Aste T. Hierarchical information clustering by means of topologically embedded graphs. PloS one. 2012;7(3):e31929.

26. Mircea M, Hochane M, Fan X, Chuva De Sousa Lopes SM, Garlaschelli D, Semrau S. Phiclust: a clusterability measure for single-cell transcriptomics reveals phenotypic subpopulations. Genome Biol [Internet]. 2022 Dec [cited 2025 July 9];23(1). Available from: https://genomebiology.biomedcentral.com/articles/10.1186/s13059-021-02590-x

27. Song WM, Zhang B. Multiscale embedded gene co-expression network analysis. PLoS computational biology. 2015;11(11):e1004574.

28. Germain PL, Sonrel A, Robinson MD. pipeComp, a general framework for the evaluation of computational pipelines, reveals performant single cell RNA-seq preprocessing tools. Genome Biol. 2020 Sept 1;21(1):227.

29. Su S, Tian L, Dong X, Hickey PF, Freytag S, Ritchie ME. CellBench: R/Bioconductor software for comparing single-cell RNA-seq analysis methods. Bioinformatics. 2020 Apr 1;36(7):2288–90.

30. Jain AK, Murty MN, Flynn PJ. Data clustering: a review. ACM computing surveys (CSUR). 1999;31(3):264–323.

31. Mahmoudi A, Jemielniak D. Proof of biased behavior of Normalized Mutual Information. Sci Rep. 2024 Apr 19;14(1):9021.

32. Tian L, Dong X, Freytag S, Lê Cao KA, Su S, JalalAbadi A, et al. Benchmarking single cell RNA-sequencing analysis pipelines using mixture control experiments. Nat Methods. 2019 June;16(6):479–87.

33. El Ayeb S, Hemery B, Jeanne F, Cherrier E, Charrier C. Evaluation Metrics for Overlapping Community Detection. In: 2022 IEEE 47th Conference on Local Computer Networks (LCN) [Internet]. Edmonton, AB, Canada: IEEE; 2022 [cited 2025 June 4]. p. 355–8. Available from: https://ieeexplore.ieee.org/document/9843473/

34. Ding J, Adiconis X, Simmons SK, Kowalczyk MS, Hession CC, Marjanovic ND, et al. Systematic comparison of single-cell and single-nucleus RNA-sequencing methods. Nat Biotechnol. 2020 June 1;38(6):737–46.

35. Lee JS, Park S, Jeong HW, Ahn JY, Choi SJ, Lee H, et al. Immunophenotyping of COVID-19 and influenza highlights the role of type I interferons in development of severe COVID-19.

Sci Immunol [Internet]. 2020 July 3 [cited 2025 July 14];5(49). Available from: https://www.science.org/doi/10.1126/sciimmunol.abd1554

36. Tailor IK, Alshehry NF, Zaidi SZ, Marei MA, Motabi IH, Alfayez M, et al. Outcome of Myeloma Patients with COVID-19 on Active Lenalidomide-Based Therapy: Does Lenalidomide Protect From Severe COVID-19? Hematol Oncol Stem Cell Ther. 2023 Jan 12;16(1):88–90.

37. Xu F, Wang G, Zhao F, Huang Y, Fan Z, Mei S, et al. IFITM3 Inhibits SARS-CoV-2 Infection and Is Associated with COVID-19 Susceptibility. Viruses. 2022 Nov 18;14(11):2553.

38. Wu SZ, Al-Eryani G, Roden DL, Junankar S, Harvey K, Andersson A, et al. A single-cell and spatially resolved atlas of human breast cancers. Nat Genet. 2021 Sept;53(9):1334–47.

39. Aran D, Hu Z, Butte AJ. xCell: digitally portraying the tissue cellular heterogeneity landscape. Genome Biol. 2017 Nov 15;18(1):220.

40. Schupp JC, Adams TS, Cosme C, Raredon MSB, Yuan Y, Omote N, et al. Integrated Single-Cell Atlas of Endothelial Cells of the Human Lung. Circulation. 2021 July 27;144(4):286–302.

41. Ghandour MS, Langley OK, Zhu XL, Waheed A, Sly WS. Carbonic anhydrase IV on brain capillary endothelial cells: a marker associated with the blood-brain barrier. Proc Natl Acad Sci U S A. 1992 Aug 1;89(15):6823–7.

42. Hänzelmann S, Castelo R, Guinney J. GSVA: Gene set variation analysis for microarray and RNA-Seq data. BMC Bioinformatics. 2013;14.

43. Curtis C, Shah SP, Chin SF, Turashvili G, Rueda OM, Dunning MJ, et al. The genomic and transcriptomic architecture of 2,000 breast tumours reveals novel subgroups. Nature. 2012 Apr 18;486(7403):346–52.

44. Győrffy B. Survival analysis across the entire transcriptome identifies biomarkers with the highest prognostic power in breast cancer. Comput Struct Biotechnol J. 2021;19:4101–9.

45. Fernández CA, Moses MA. Modulation of angiogenesis by tissue inhibitor of metalloproteinase-4. Biochem Biophys Res Commun. 2006 June 23;345(1):523–9.

46. Fang F, Wasserman SM, Torres-Vazquez J, Weinstein B, Cao F, Li Z, et al. The role of Hath6, a newly identified shear-stress-responsive transcription factor, in endothelial cell differentiation and function. J Cell Sci. 2014 Apr 1;127(Pt 7):1428–40.

47. Charlestin V, Fulkerson D, Arias Matus CE, Walker ZT, Carthy K, Littlepage LE. Aquaporins: New players in breast cancer progression and treatment response. Front Oncol. 2022;12:988119.

48. Ali YB, Carrière F, Verger R, Petry S, Muller G, Abousalham A. Continuous monitoring of cholesterol oleate hydrolysis by hormone-sensitive lipase and other cholesterol esterases. J Lipid Res. 2005 May;46(5):994–1000.

49. Trudeau RJ, Trudeau RJ. Introduction to graph theory. New York: Dover Pub.; 1993. x, 209 p. p. (Dover books on advanced mathematics).

50. Song WM, Agrawal P, Von Itter R, Fontanals-Cirera B, Wang M, Zhou X, et al. Network models of primary melanoma microenvironments identify key melanoma regulators underlying prognosis. Nature communications. 2021;12(1):1–14.

51. Song WM, Lin X, Liao X, Hu D, Lin J, Sarpel U, et al. Multiscale network analysis reveals molecular mechanisms and key regulators of the tumor microenvironment in gastric cancer. International journal of cancer. 2020;146(5):1268–80.

52. Song WM, Elmas A, Farias R, Xu P, Zhou X, Hopkins B, et al. Multiscale protein networks systematically identify aberrant protein interactions and oncogenic regulators in seven cancer types. J Hematol Oncol. 2023 Dec 15;16(1):120.

53. Choi H, Song W min, Wang M, Sram RJ, Zhang B. Benzo [a] pyrene is associated with dysregulated myelo-lymphoid hematopoiesis in asthmatic children. Environment international. 2019;128:218–32.

54. McKenzie AT, Moyon S, Wang M, Katsyv I, Song WM, Zhou X, et al. Multiscale network modeling of oligodendrocytes reveals molecular components of myelin dysregulation in Alzheimer’s disease. Molecular neurodegeneration. 2017;12(1):1–20.

55. Wang Q, Zhang Y, Wang M, Song WM, Shen Q, McKenzie A, et al. The landscape of multiscale transcriptomic networks and key regulators in Parkinson’s disease. Nature communications. 2019;10(1):1–15.

56. Wang M, Li A, Sekiya M, Beckmann ND, Quan X, Schrode N, et al. Transformative Network Modeling of Multi-omics Data Reveals Detailed Circuits, Key Regulators, and Potential Therapeutics for Alzheimer’s Disease. Neuron. 2021 Jan 20;109(2):257-272.e14.

57. Forst CV, Zhou B, Wang M, Chou TW, Mason G, Song W min, et al. Integrative gene network analysis identifies key signatures, intrinsic networks and host factors for influenza virus A infections. NPJ systems biology and applications. 2017;3(1):1–16.

58. Cleveland WS, Devlin SJ. Locally weighted regression: an approach to regression analysis by local fitting. Journal of the American statistical association. 1988;83(403):596–610.

59. Traag VA, Waltman L, van Eck NJ. From Louvain to Leiden: guaranteeing well-connected communities. Sci Rep. 2019 Mar 26;9(1):5233.

60. Reichardt J, Bornholdt S. Statistical mechanics of community detection. Phys Rev E Stat Nonlin Soft Matter Phys. 2006 July;74(1 Pt 2):016110.

61. Song WM, Di Matteo T, Aste T. Building complex networks with Platonic solids. Physical Review E. 2012;85(4):046115.

62. McGinnis CS, Murrow LM, Gartner ZJ. DoubletFinder: Doublet Detection in Single-Cell RNA Sequencing Data Using Artificial Nearest Neighbors. Cell Syst. 2019 Apr 24;8(4):329-337 e4.

63. Linderman GC, Zhao J, Roulis M, Bielecki P, Flavell RA, Nadler B, et al. Zero-preserving imputation of single-cell RNA-seq data. Nat Commun. 2022 Jan 11;13(1):192.

64. Hafemeister C, Satija R. Normalization and variance stabilization of single-cell RNA-seq data using regularized negative binomial regression. Genome Biol. 2019 Dec 23;20(1):296.

65. Butler A, Hoffman P, Smibert P, Papalexi E, Satija R. Integrating single-cell transcriptomic data across different conditions, technologies, and species. Nat Biotechnol. 2018 June;36(5):411–20.

66. Lun AT, Bach K, Marioni JC. Pooling across cells to normalize single-cell RNA sequencing data with many zero counts. Genome Biol. 2016 Apr 27;17:75.

67. Aran D, Looney AP, Liu L, Wu E, Fong V, Hsu A, et al. Reference-based analysis of lung single-cell sequencing reveals a transitional profibrotic macrophage. Nat Immunol. 2019 Feb;20(2):163–72.

68. Monaco G, Lee B, Xu W, Mustafah S, Hwang YY, Carré C, et al. RNA-Seq Signatures Normalized by mRNA Abundance Allow Absolute Deconvolution of Human Immune Cell Types. Cell Rep. 2019 Feb 5;26(6):1627-1640.e7.

69. Mabbott NA, Baillie JK, Brown H, Freeman TC, Hume DA. An expression atlas of human primary cells: inference of gene function from coexpression networks. BMC Genomics. 2013 Sept 20;14:632.

70. Finak G, McDavid A, Yajima M, Deng J, Gersuk V, Shalek AK, et al. MAST: a flexible statistical framework for assessing transcriptional changes and characterizing heterogeneity in single-cell RNA sequencing data. Genome Biol. 2015 Dec 10;16:278.

71. Robinson MD, Oshlack A. A scaling normalization method for differential expression analysis of RNA-seq data. Genome Biol. 2010;11(3):R25.

72. Song WM. Data sets for single-cell multi-scale clustering workflow [Internet]. Synapse; 2023 [cited 2025 Aug 25]. Available from: https://doi.org/10.7303/SYN52966803

73. Edgar R, Domrachev M, Lash AE. Gene Expression Omnibus: NCBI gene expression and hybridization array data repository. Nucleic Acids Res. 2002 Jan 1;30(1):207–10.

74. Tarhan L, Bistline J, Chang J, Galloway B, Hanna E, Weitz E. Single Cell Portal: an interactive home for single-cell genomics data [Internet]. Scientific Communication and Education; 2023 [cited 2025 Aug 25]. Available from: http://biorxiv.org/lookup/doi/10.1101/2023.07.13.548886

75. Song WM. Single-cell Multi-Scale Clustering (MSC) [Internet]. 2025. Available from: https://doi.org/10.5281/zenodo.16895568

76. Song WM, Ming C, Forst CV, Zhang B. Multiscale clustering (MSC) for scRNA-seq data (Version 0.4) [Internet]. Available from: https://archive.softwareheritage.org/swh:1:snp:9c83a426405d2b946be280f16ecee4b2e87fa224

77. Song WM, Chen M, Forst CV, Zhang B. Multi-scale single-cell clustering (MSC) workflow [Internet]. Available from: https://doi.org/10.48546/WORKFLOWHUB.WORKFLOW.1875.1

78. Song WM. Developmental versions of single-cell multi-scale clustering (MSC) [Internet]. Available from: https://github.com/songlabcodes/MSC

79. Korsunsky I, Millard N, Fan J, Slowikowski K, Zhang F, Wei K, et al. Fast, sensitive and accurate integration of single-cell data with Harmony. Nat Methods. 2019 Dec;16(12):1289–96.

80. Song W; Ming C; Forst CV; Zhang B. Supporting data for "Unsupervised multi-scale clustering of single-cell transcriptomes to identify hierarchical structures of cell subtypes" GigaScience Database; 2025. https://doi.org/10.5524/102753

Figure 1

Click here to access/download;Figure;Figure1.tif

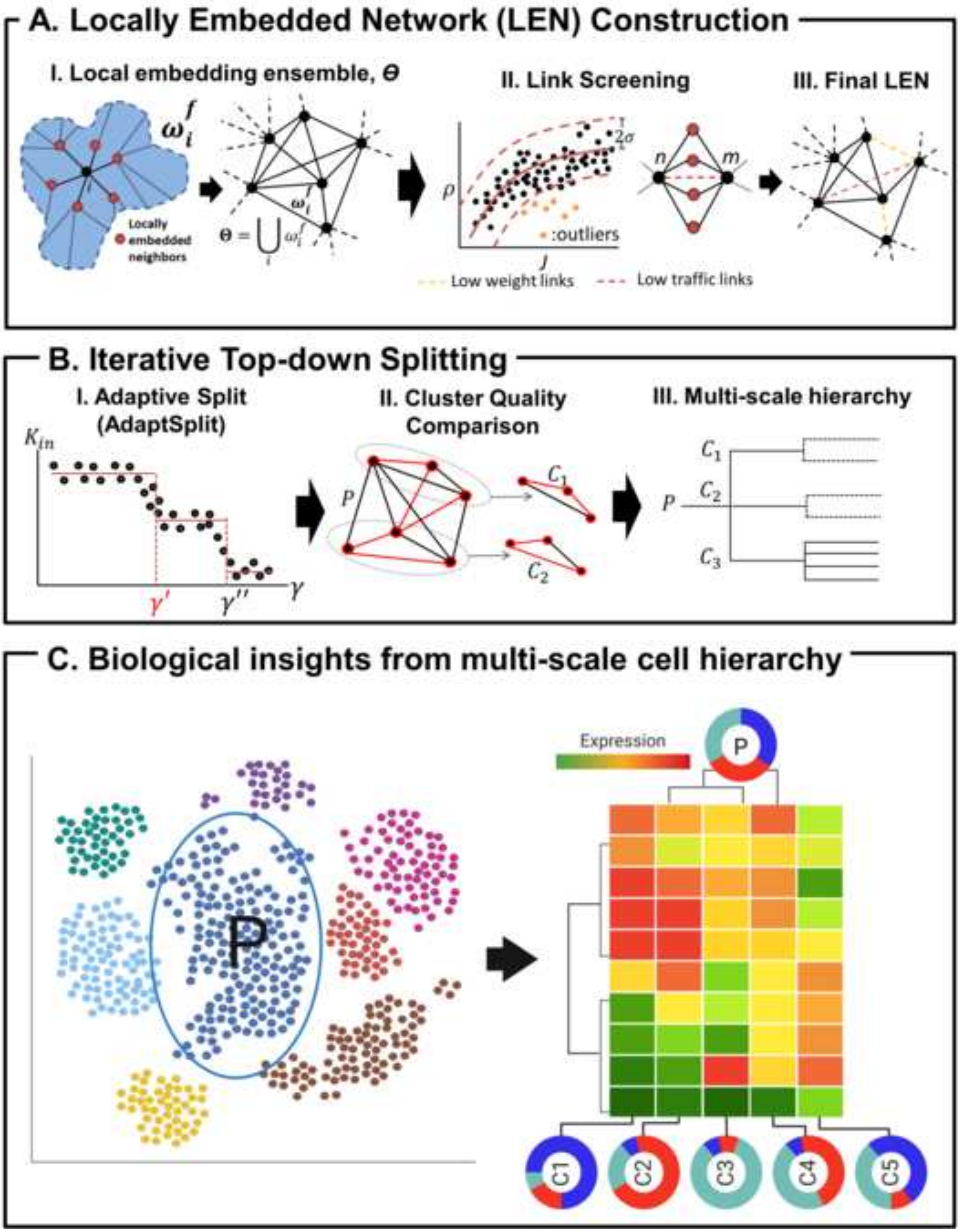

Figure 2

Click here to access/download;Figure;Figure2.pdf

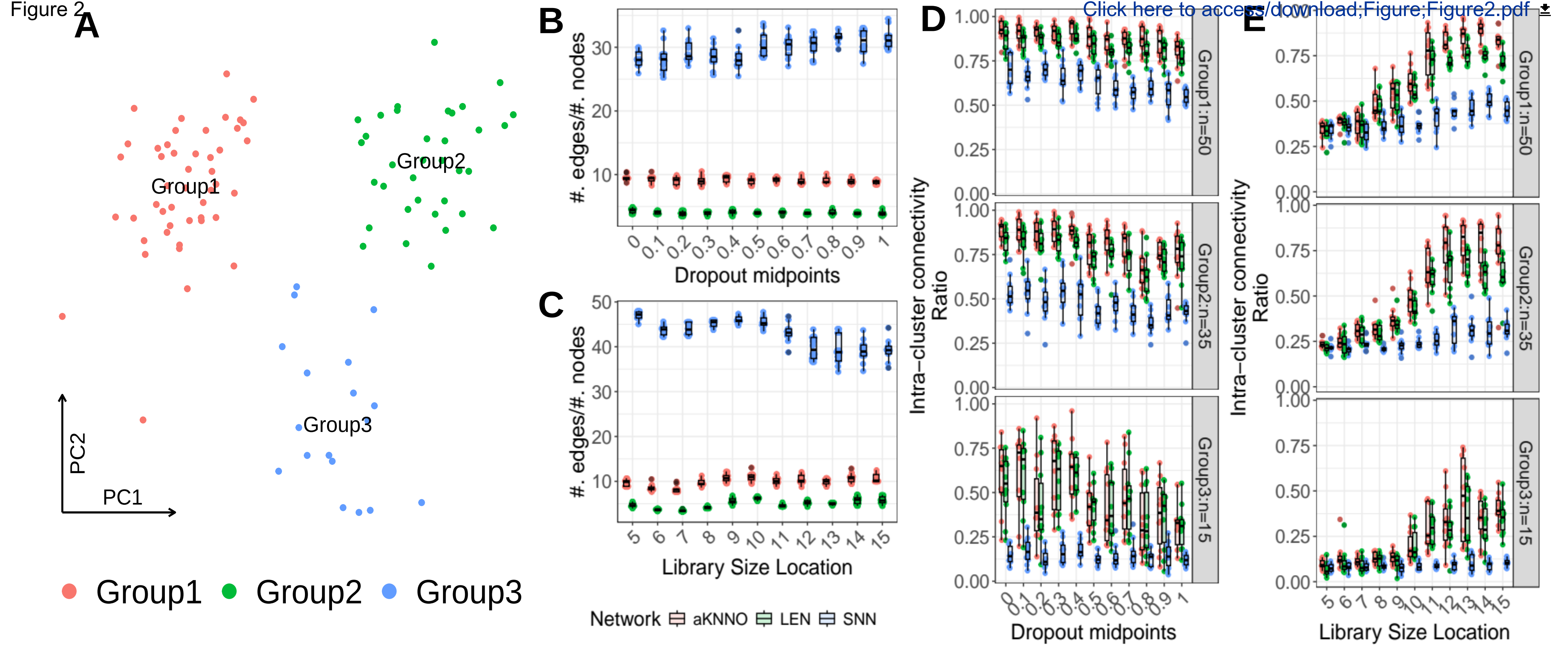

Figure 3

Click here to access/download;Figure;Figure3.pdf

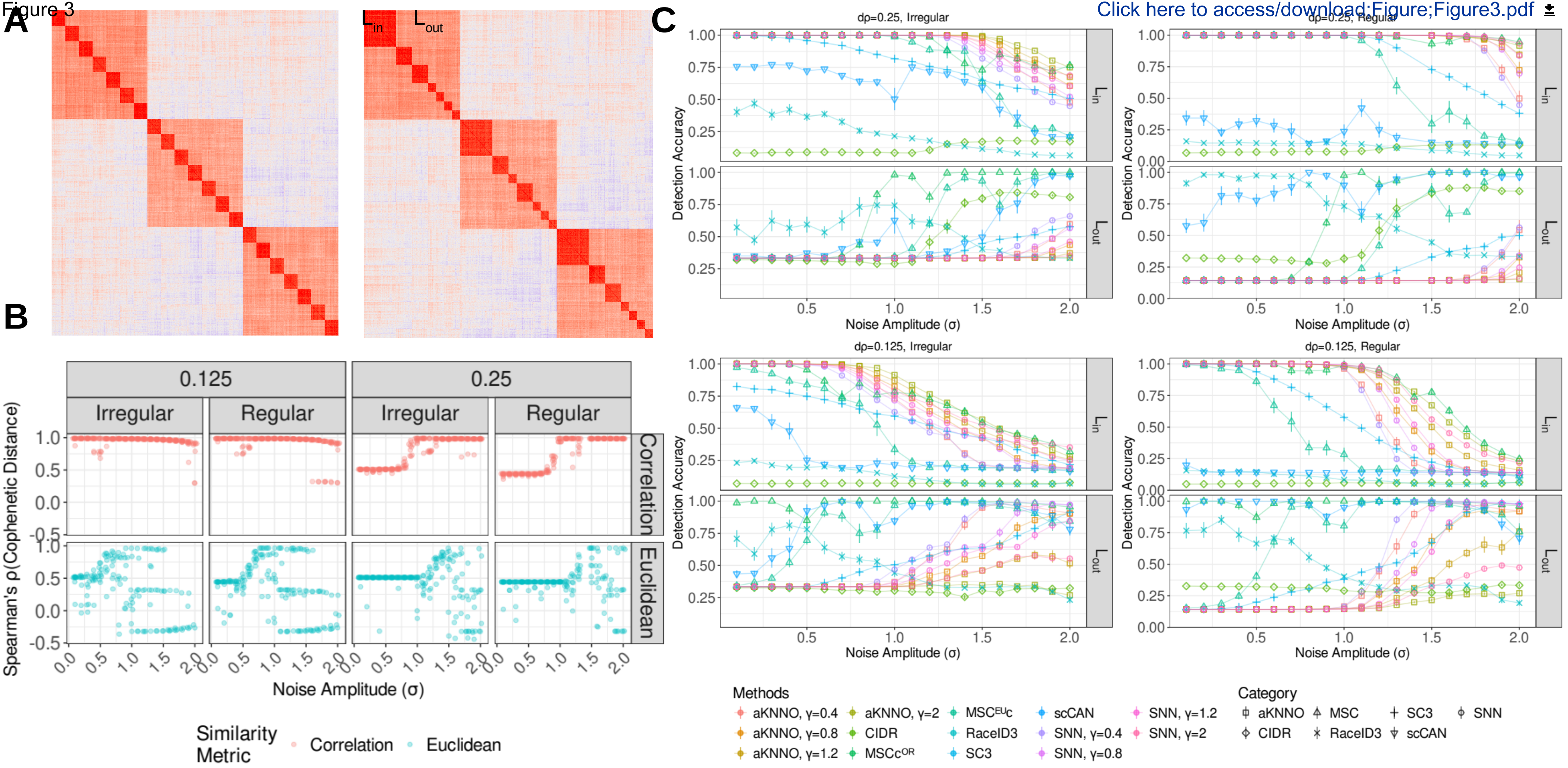

Figure 4

Click here to access/download;Figure;Figure4.pdf

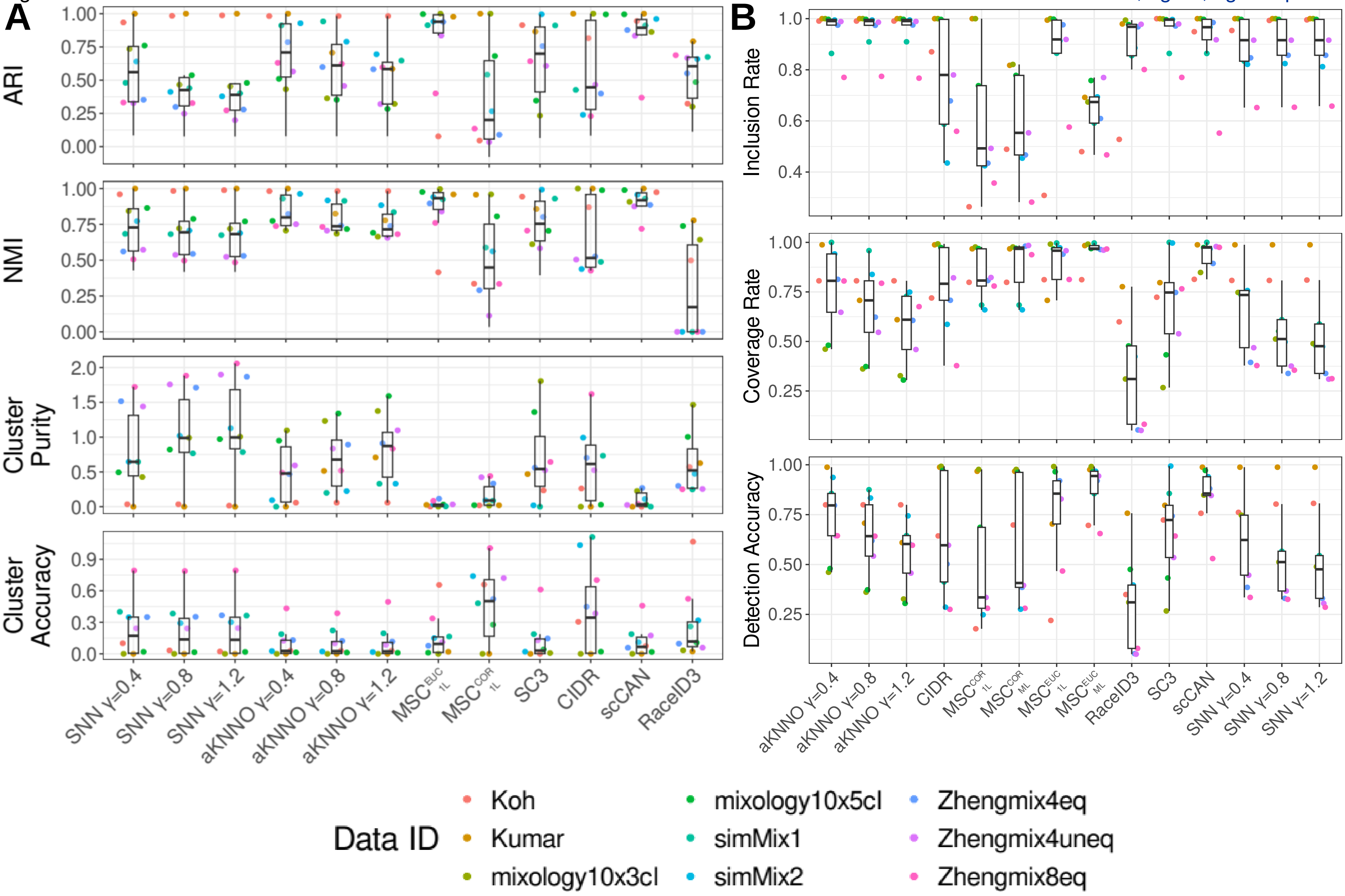

Figure 5

Click here to access/download;Figure;Figure5.pdf

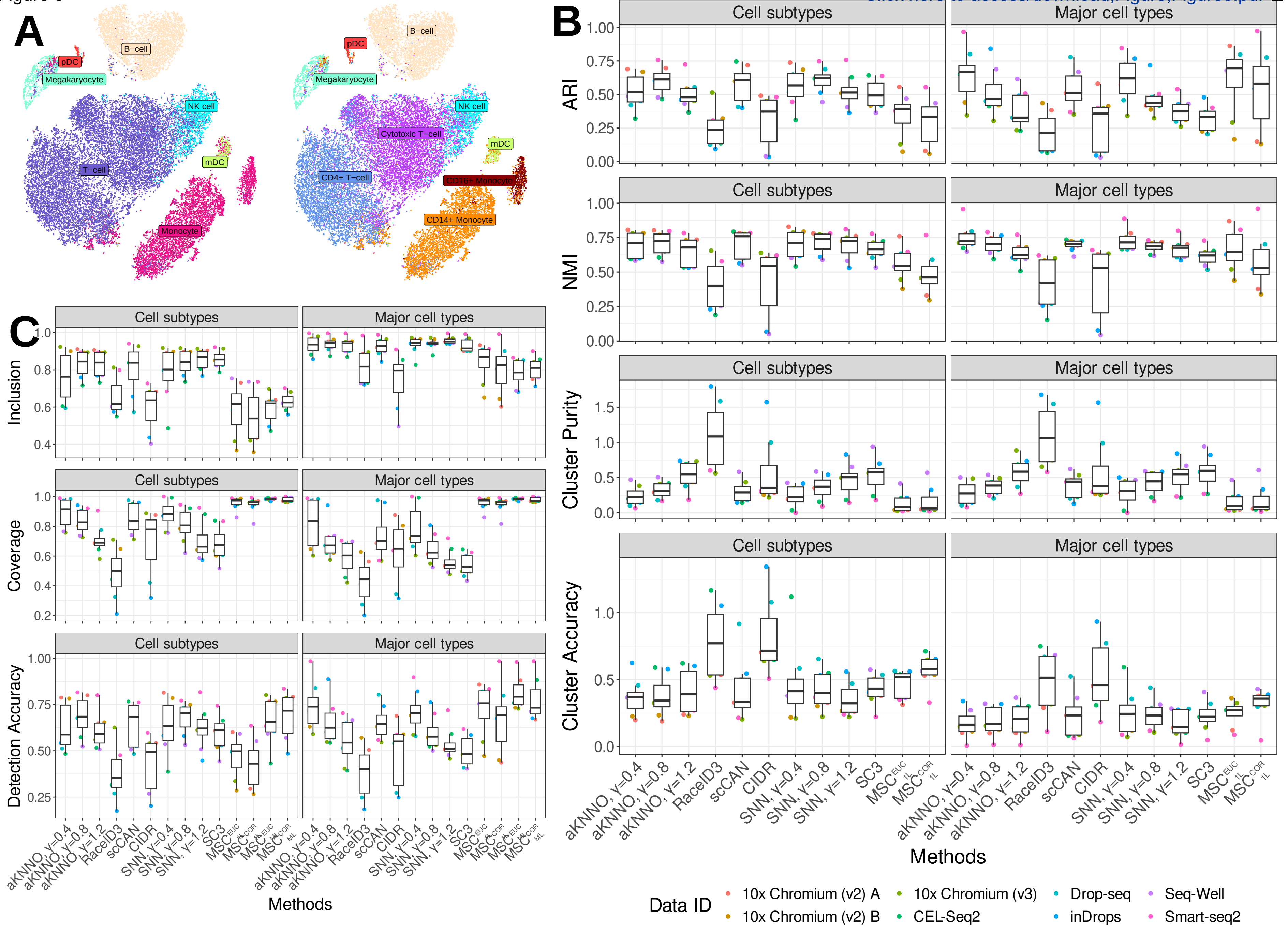

Figure 6

Click here to access/download;Figure;Figure6.tiff

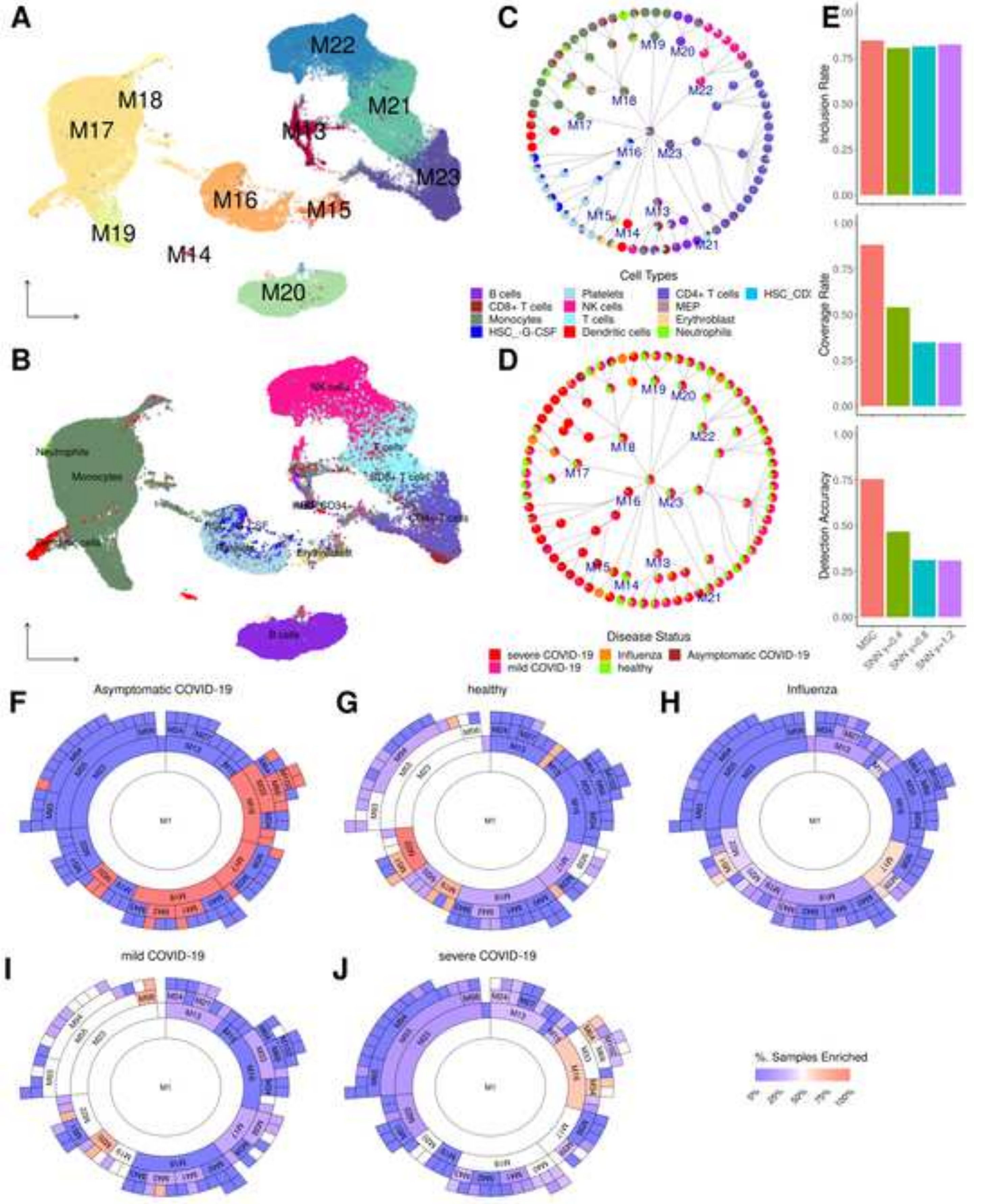

Figure 7

Click here to access/download;Figure;Figure7.tif

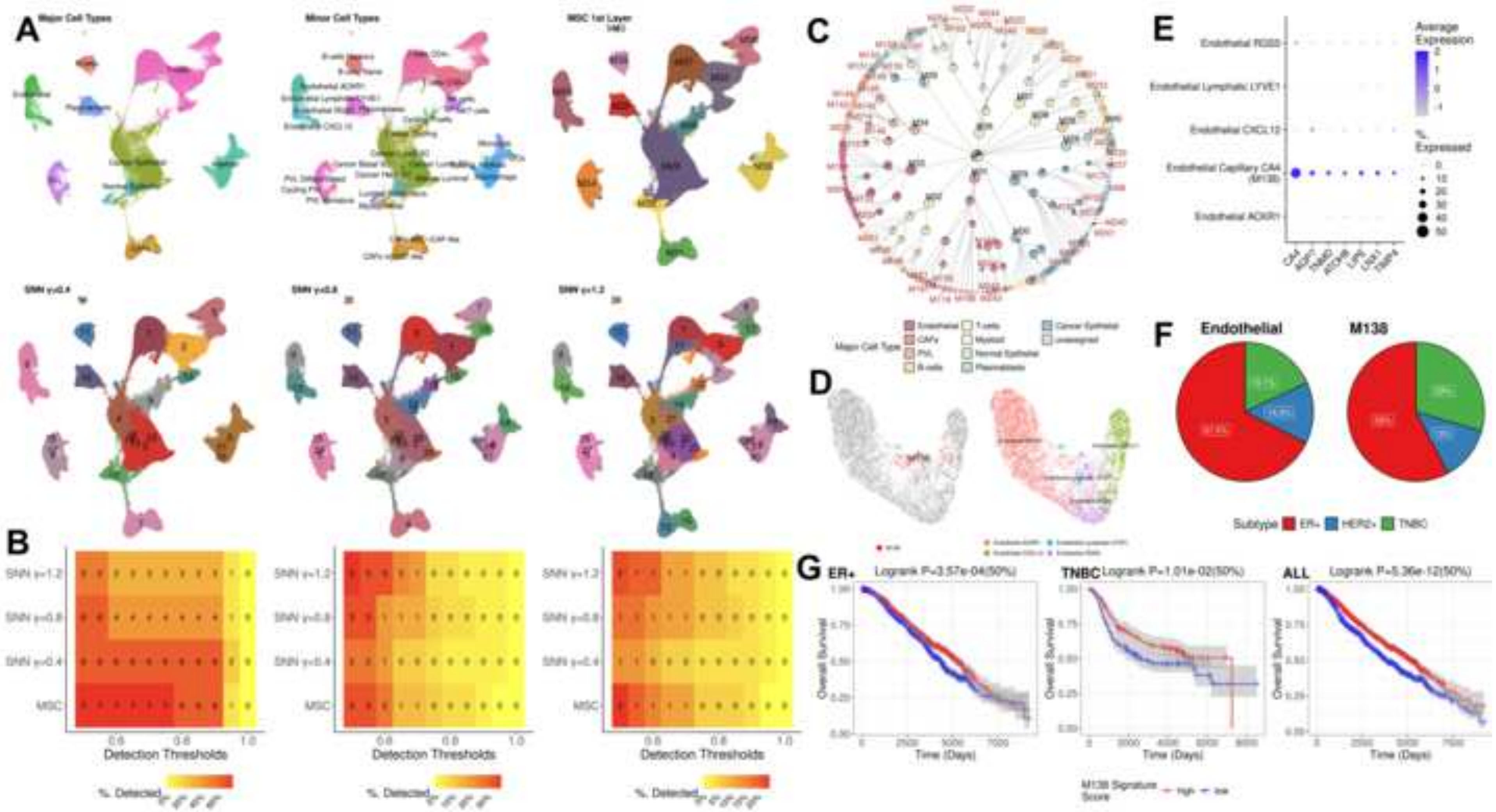

Click here to access/download
Supplementary Material
SUPPLEMENTARY MATERIAL.pdf

Supplementary Data 1

Click here to access/download
Supplementary Material
Supplementary_Data_1.xlsx

Supplementary Data 2

Click here to access/download
Supplementary Material
Supplementary_Data_2.xlsx

Responses to Editorial Comments

We sincerely appreciate the comments, which provide us with further insights and opportunities to further improve the manuscript. We have revised the manuscript according to the comments, and we provide a point-by-point response to each suggestion below. Responses to all of the comments are provided below with our responses given in blue.

1) Please register any new software application to RRID and biotools and Computational workflows should be registered in WorkflowHub. Have the DOIs cited in the relevant places in the manuscript.

We have registered the MSC software to RRID (SCR_027342), biotools (single-cell_multi-scale_clustering_msc) and its workflow to WorkflowHub (DOI: 10.48546/WORKFLOWHUB.WORKFLOW.1875.1).

The DOIs and URLs for example data in Synapse (Ref #. 72, Page 39, line 802), software and example codes in Zenodo (Ref #. 75, Page 40, line 819), Software Heritage (Ref #. 76, Page 40, line 820) the examples codes as workflow in Workflow Hub (Ref #. 77, Page 40, line 820) are cited as respective references in the main text.

The URLs for GEO and Single-cell Portal were replaced with respective references for their white papers for the databases. Specifically, GEO was cited in Ref #. 73, and Single-cell Portal was cited in Ref #. 74 in the main text bibliography.

2) Please upload an editable .docx version of your manuscript, not PDF

We have replaced the the .pdf file for .docx file in this round of revision for the main text.

3) Please reformat abstract to have 3 sections Background, Results, conclusions

We have reformatted the abstract in the requested three sections in Page 2, line 22 – line 34 in the revised main text.

4) Please insert missing sections required in Availability of source code and requirements:

We have inserted “Availability of source code and requirements” section and its contents in Page 40, line 822 – line 833.

5) Move Declaration after "Data Availability"

The “Data Availability” section has been added to Page 39, line 800 – Page 40, line 833.

6) Removal of URLs and adding appropriate citations.

Aforementioned URLs and DOIs in the main text from 1) have been moved to bibliography and have been cross-referenced numerically as instructed.

7) Adding Github project citations
"A snapshot of our GitHub project is archived in Software Heritage [Ref A]. And the workflow is also available in Workflow hub [RefB]."

Citations to add to references in the EXACT format as follows:

Ref A:
Song W, Ming C, Forst CV and Zhang B (2025) Multiscale clustering (MSC) for scRNA-seq data (Version 0.4). [Computer software]. Software Heritage, https://archive.softwareheritage.org/swh:1:snp:9c83a426405d2b946be280f16ecee4b2e87fa224

Ref B:
Song, W.-M. (2025). Multi-scale single-cell clustering (MSC) workflow. WorkflowHub. https://doi.org/10.48546/WORKFLOWHUB.WORKFLOW.1875.1

The sentence has been added into Page 40, line 819 – line 820. The Software Heritage and Workflow Hub were cited as Ref #. 76 and 77 accordingly.
