# Supplementary material for: Unsupervised multiscale clustering of single-cell transcriptomes to identify hierarchical structures of cell subtypes
Source: Gigascience. 2025 Oct 9;14:giaf111. doi: 10.1093/gigascience/giaf111 (PMC12509883; doi:10.1093/gigascience/giaf111)
Supplement: giaf111_Authors_Response_To_Reviewer_Comments_Original_Submission [file giaf111_authors_response_to_reviewer_comments_original_submission.pdf]

# Author's Response To Reviewer Comments

(We have provided a separate .pdf file as the responses reviewers' comments in a more structured letter. Belows are the texts from the responses.)

Reviewer #1: MSC presents a well-structured approach to hierarchical clustering in scRNA-seq data, demonstrating strong performance across multiple datasets. Its ability to identify novel cell subpopulations and disease-relevant mechanisms makes it a useful tool for single-cell analysis. I have some comments as below.

1) While MSC is compared against traditional clustering methods, it would be valuable to assess its performance relative to emerging deep learning-based clustering techniques, such as variational autoencoders or graph neural networks, which have shown promise in single-cell clustering tasks: We appreciate reviewer's insightful suggestion. We have included single-cell Clustering using Autoencoder and Network fusion (scCAN), one of the latest methods on that makes use of stacked variational autoencoder, as one of the benchmark methods for comparative analyses throughout the revised manuscript. These comparative analyses include simulation study (Page 8, line 177 – Page 12, line 272), gold standard data study (Page 13, line 295 – Page 15, line 348) and cross-platform study using Ding et al. 2020 data (Page 15, line 349 – Page17, line 384).

2) Although LEN is designed to construct sparse networks, the study does not extensively discuss its sensitivity to data sparsity or noise in scRNA-seq datasets. A deeper analysis of how LEN performs under varying sequencing depths and dropout rates would be beneficial: We thank the reviewer for the constructive comments. To address this, we have utilized splatter framework, a model-based scRNA-seq data simulator, to generate simulated single-cell data sets with varying sequencing depths and dropout rates. These simulated data facilitated objective evaluations of cell similarity network topology to align with the underlying clustering structure. LEN was compared against the most broadly used shared nearest neighbor (SNN) networks and its variant, adaptive kNN method (aKNNO). The results showed that, while LEN and aKNNO outperformed SNN and comparable performances, LEN was the only sparse network to overcome the inherent resolution limit dictated by the edge density in the networks, and these demonstrate the utility of LEN to show robust performances across varying noise sources while maintaining its sparsity (Page 7, line 141 – Page 8, line 176).

3) The AdaptSplit method dynamically selects the optimal resolution for clustering. However, further justification is needed on how the compactness and intra-cluster connectivity metrics influence the termination of iterative splits. The impact of different parameter settings should be systematically explored: We thank the reviewer's constructive comment. To facilitate objective evaluations of how these factors affect detecting correct cluster hierarchies in data, we utilized multivariate Gaussian generator with hierarchical clusters across varying noises. We have evaluated the behavior of α parameter in the compactness, υ(α). Specifically, the iterative split terminates when the parent and child compactness coincides, and this can be effectively captured by the breaking α′ values for υparent(α′) = υchild(α′). Using the simulated hierarchical data, we observed that more coherent and regular cluster sizes yielded higher α′, hence α′ adaptively reflected the structural characteristics in clustering structures in the data. Further, the simulated data showed that the more compact clusters than their parents showed significant intra-cluster connectivity, and the compactness criteria dictated the termination (Page 12, line 273 – Page 13, line 294).
4) While MSC successfully identifies hierarchical cellular structures, the paper lacks a quantitative assessment of how well the detected hierarchies align with known biological differentiation pathways. Metrics such as hierarchical purity or adjusted mutual information could be employed for more rigorous validation: To address this, we have utilized benchmark PBMC data set from 10x containing the hematopoietic lineages, and compared the MSC-inferred cell hierarchies to these lineages (Page 11, line 252 – Page 12, line 262). We have performed in-depth analyses to evaluate each clustering methods to detect different levels of ground-truth cellular hierarchy. Further, we have implemented normalized mutual information to evaluate the clustering results throughout the manuscript.

6) While MSC was applied to multiple datasets, its generalizability to independent scRNA-seq datasets from

different platforms (e.g., Smart-seq vs. 10x Genomics) was not explicitly discussed and compared with existing methods (e.g. PMID: 34158507): This is indeed a crucial aspect of performance evaluation, and we thank the reviewer for the constructive comment. We have utilized the suggested cross-platform PBMC scRNA-seq data sets from Ding et al. 2020 (PMID: 34158507), and applied MSC and the benchmark methods. These results revealed that MSC is one of the best performing methods across different platforms. (Page 15, line 349 – Page 17, line 384)

Reviewer #2: In this study, Song et al. developed a multi-scale clustering (MSC) approach to identify cell subpopulations, which is independent of k and automatically operates at multiple resolutions. MSC demonstrated improved performance in both simulated and real datasets. Below are my comments:

1) Numerous single-cell clustering methods have been developed, including SC3, RaceID3, CIDR, BackSPIN, SINCERA, SIMLR, GiniClust, DR-SC, and adaptive k-NN approaches like aKNNO, as well as multi-resolution clustering methods like MultiK. To provide a more comprehensive evaluation, MSC should be compared against a broader range of methods beyond SC3 and SNN: We have expanded the pool of benchmark clustering methods for comparative evaluations. In addition to the existing benchmark methods in the intial submission (SC3, CIDR, SNN-based Louvain clustering), we have added aKNNO-based clustering at different resolutions, RaceID3 and variational autoencoder based scCAN. We remark that, while we made our best attempts to add more methods per the reviewer's suggestions, some of the methods could not be all tested on the same ground due to bugs, scalability issues for data sets with size > 10000 cells, and near-impossible installation processes due to outdated dependencies. The updated results are reflected through the revised manuscript including simulation data study (Page 8, line 177 – Page 12, line 272), gold standard data study (Page 13, line 295 – Page 15, line 348) and cross-platform PBMC scRNA-seq study (Page 13, line 295 – Page 15, line 348).

2) To assess whether clustering leads to over- or under-clustering, various statistical approaches can be used to determine whether a subcluster represents true biological structure or merely statistical noise. Methods such as PhiClust and significance analysis of hierarchical clustering (Nature Methods, vol. 20, pp. 1196-1202, 2023) should be considered for evaluation: We have incorporated PhiClust to evaluate clustering structures captured by MSC in the simulated data. Specifically, we focused on PhiClust as a statistical measure of clusterability for a group of cells, and used this aspect to evaluate clusterability of parent clusters identified in MSC. This facilitated the evaluation of ground-truth parent cluster detection in the simulated data sets by searching for clusterable clusters in which MSC identified subcluster structures. Similarly, this also facilitated detection of the ground-truth child clusters in the simulated data sets by searching for unclusterable clusters by MSC with no further splits. The results are discussed in Page 10, line 229 – Page 11, line 242.

3) Adjusted Rand Index (ARI) was used as a performance metric. However, ARI is highly dependent on the number of clusters, which may introduce bias. For a fair comparison, it would be preferable to ensure that each method generates the same number of clusters: We thank the reviewer's constructive comments. However, we could not enforce the same number of clusters (k) for all clustering methods evaluated in this manuscript. This includes graph-theoretic clustering methods at different resolutions (SNN, aKNNO and MSC) where there is no direct known relationship between the resolution parameter ($\gamma$) and k. To mitigate this to the best of our ability, we have also utilized normalized mutual information (NMI), a information-theoretic evaluation metric that is independent of the number of clusters. NMI has been incorporated in Figures 3-5.

4) The resolution of all figures is low, making them difficult to interpret, even when zoomed in. Higher-resolution images should be provided: To ensure the high resolutions of the figures, we have replaced many figures into .pdf, which would facilitate the resolution issues (Figures 2-5).

5) The method's name should be used consistently throughout the manuscript. It is referred to as MSC in the text but labeled as "AdaptSplit" in some figures. Please unify the terminology: To address this, we have replaced the label from AdaptSplit to MSC1L to emphasize that AdaptSplit realizes the single-layer of clustering structure in the hierarchy throughout the manuscript.

6) Are there specific scenarios where one metric (correlation or Euclidean) is preferable over the other? As we discussed in the results, we find that Euclidean metric is preferred for most real scRNA-seq data (Page 15, lines 334-341; Page 16, lines 361-364).

Editorial Manager®

## Cookie Preference Center

We use cookies which are necessary to make our site work. We may also use additional cookies to analyze, improve and personalize our content and your digital experience. For more information, see our Cookie Policy.

You may choose not to allow some types of cookies. However, blocking some types may impact your experience of our site and the services we are able to offer. See the different category headings below to find out more or change your settings.

Allow all

## Manage Consent Preferences

### Strictly Necessary Cookies

**Always active**

These cookies are necessary for the website to function and cannot be switched off in our systems. They are usually only set in response to actions made by you which amount to a request for services, such as setting your privacy preferences, logging in or filling in forms. You can set your browser to block or alert you about these cookies, but some parts of the site will not then work.

### Functional Cookies

These cookies enable the website to provide enhanced functionality and personalisation. They may be set by us or by third party providers whose services we have added to our pages. If you do not allow these cookies then some or all of these services may not function properly.

#### Performance Cookies

These cookies allow us to count visits and traffic sources so we can measure and improve the performance of our site. They help us to know which pages are the most and least popular and see how visitors move around the site.

#### Targeting Cookies

These cookies may be set through our site by our advertising partners. They may be used by those companies to build a profile of your interests and show you relevant adverts on other sites. If you do not allow these cookies, you will experience less targeted advertising.

### Cookie List

Clear

Apply Cancel
Consent Leg.Interest

Confirm my choices

Powered by onetrust
