# Supplementary material for: Unsupervised multiscale clustering of single-cell transcriptomes to identify hierarchical structures of cell subtypes
Source: Gigascience. 2025 Oct 9;14:giaf111. doi: 10.1093/gigascience/giaf111 (PMC12509883; doi:10.1093/gigascience/giaf111)
Supplement: giaf111_Reviewer_1_Report_Original_Submission — Qianqian Song -- 2/8/2025 [file giaf111_reviewer_1_report_original_submission.pdf]

Reviewer Report

Title: Unsupervised multi-scale clustering of single-cell transcriptomes to identify hierarchical structures of cell subtypes

Version: Original Submission **Date:** 2/8/2025

Reviewer name: Qianqian Song

Reviewer Comments to Author:

MSC presents a well-structured approach to hierarchical clustering in scRNA-seq data, demonstrating strong performance across multiple datasets. Its ability to identify novel cell subpopulations and disease-relevant mechanisms makes it a useful tool for single-cell analysis. I have some comments as below.

1) While MSC is compared against traditional clustering methods, it would be valuable to assess its performance relative to emerging deep learning-based clustering techniques, such as variational autoencoders or graph neural networks, which have shown promise in single-cell clustering tasks.

2) Although LEN is designed to construct sparse networks, the study does not extensively discuss its sensitivity to data sparsity or noise in scRNA-seq datasets. A deeper analysis of how LEN performs under varying sequencing depths and dropout rates would be beneficial.

3) The AdaptSplit method dynamically selects the optimal resolution for clustering. However, further justification is needed on how the compactness and intra-cluster connectivity metrics influence the termination of iterative splits. The impact of different parameter settings should be systematically explored.

4) While MSC successfully identifies hierarchical cellular structures, the paper lacks a quantitative assessment of how well the detected hierarchies align with known biological differentiation pathways. Metrics such as hierarchical purity or adjusted mutual information could be employed for more rigorous validation.

5) While MSC was applied to multiple datasets, its generalizability to independent scRNA-seq datasets from different platforms (e.g., Smart-seq vs. 10x Genomics) was not explicitly discussed and compared with existing methods (e.g. PMID: 34158507).

Methods

Are the methods appropriate to the aims of the study, are they well described, and are necessary controls included? Choose an item.

Conclusions

Are the conclusions adequately supported by the data shown? Choose an item.

Reporting Standards

Does the manuscript adhere to the journal's guidelines on minimum standards of reporting? Choose an item.

Choose an item.

Statistics

Are you able to assess all statistics in the manuscript, including the appropriateness of statistical tests used? Choose an item.

Quality of Written English

Please indicate the quality of language in the manuscript: Choose an item.

Declaration of Competing Interests

Please complete a declaration of competing interests, considering the following questions:

- Have you in the past five years received reimbursements, fees, funding, or salary from an organisation that may in any way gain or lose financially from the publication of this manuscript, either now or in the future?
- Do you hold any stocks or shares in an organisation that may in any way gain or lose financially from the publication of this manuscript, either now or in the future?
- Do you hold or are you currently applying for any patents relating to the content of the manuscript?
- Have you received reimbursements, fees, funding, or salary from an organization that holds or has applied for patents relating to the content of the manuscript?
- Do you have any other financial competing interests?
- Do you have any non-financial competing interests in relation to this paper?

If you can answer no to all of the above, write 'I declare that I have no competing interests' below. If your reply is yes to any, please give details below.

I declare that I have no competing interests.

I agree to the open peer review policy of the journal. I understand that my name will be included on my report to the authors and, if the manuscript is accepted for publication, my named report including any attachments I upload will be posted on the website along with the authors' responses. I agree for my report to be made available under an Open Access Creative Commons CC-BY license (http://creativecommons.org/licenses/by/4.0/). I understand that any comments which I do not wish to be included in my named report can be included as confidential comments to the editors, which will not be published.

Choose an item.

To further support our reviewers, we have joined with Publons, where you can gain additional credit to further highlight your hard work (see: https://publons.com/journal/530/gigascience). On publication of this paper, your review will be automatically added to Publons, you can then choose whether or not to claim your Publons credit. I understand this statement.

Yes Choose an item.
